# Id genes are essential for early heart formation

**DOI:** 10.1101/gad.300400.117

**Published:** 2017-07-01

**Authors:** Thomas J. Cunningham, Michael S. Yu, Wesley L. McKeithan, Sean Spiering, Florent Carrette, Chun-Teng Huang, Paul J. Bushway, Matthew Tierney, Sonia Albini, Mauro Giacca, Miguel Mano, Pier Lorenzo Puri, Alessandra Sacco, Pilar Ruiz-Lozano, Jean-Francois Riou, Muriel Umbhauer, Gregg Duester, Mark Mercola, Alexandre R. Colas

**Affiliations:** 1 Sanford Burnham Prebys Medical Discovery Institute, La Jolla, California, 92037, USA;; 2 Department of Bioengineering, University of California at San Diego, La Jolla, California 92037, USA;; 3 Graduate School of Biomedical Sciences, Sanford Burnham Prebys Medical Discovery Institute, La Jolla, California 92037, USA;; 4 Department of Medicine and Cardiovascular Institute, Stanford University, Palo Alto, California 94305, USA;; 5 International Centre for Genetic Engineering and Biotechnology, 34149 Trieste, Italy;; 6 Center for Neuroscience and Cell Biology (CNC), University of Coimbra, 3004-504 Coimbra, Portugal;; 7 Istituti di Ricovero e Cura a Carattere Scientifico, Fondazione Santa Lucia, 00179 Rome, Italy;; 8 Regencor, Inc., Los Altos, California 94022, USA;; 9 UMR 7622 Developmental Biology, Sorbonne Universités, University Pierre and Marie Curie, F- 75005 Paris, France

**Keywords:** cardiac progenitors, cardiac mesoderm specification, heartless, Id proteins, CRISPR/Cas9-mediated quadruple knockout, platform for cardiac disease modeling and drug discovery

## Abstract

Deciphering the fundamental mechanisms controlling cardiac specification is critical for our understanding of how heart formation is initiated during embryonic development and for applying stem cell biology to regenerative medicine and disease modeling. Using systematic and unbiased functional screening approaches, we discovered that the Id family of helix–loop–helix proteins is both necessary and sufficient to direct cardiac mesoderm formation in frog embryos and human embryonic stem cells. Mechanistically, Id proteins specify cardiac cell fate by repressing two inhibitors of cardiogenic mesoderm formation—Tcf3 and Foxa2—and activating inducers Evx1, Grrp1, and Mesp1. Most importantly, CRISPR/Cas9-mediated ablation of the entire Id (Id1–4) family in mouse embryos leads to failure of anterior cardiac progenitor specification and the development of heartless embryos. Thus, Id proteins play a central and evolutionarily conserved role during heart formation and provide a novel means to efficiently produce cardiovascular progenitors for regenerative medicine and drug discovery applications.

## Abstract (precis)

Cunningham et al. demonstrate that the Id family of helix–loop–helix proteins is both necessary and sufficient to direct cardiogenic mesoderm formation from frog embryos to human embryonic stem cells.

Heart formation begins during gastrulation with the specification of cardiogenic mesoderm progenitors (CMPs) that migrate anteriorly to form the cardiac primordium that assembles into the fully formed heart (Buckingham et al. 2005; Kelly et al. 2014; Meilhac et al. 2015). Intense research over the past two decades has led to the identification of extracellular signals that initiate cardiogenesis (Schultheiss et al. 1997; Marvin et al. 2001; Schneider and Mercola 2001; Pandur et al. 2002; Collop et al. 2006; Kattman et al. 2006; Foley et al. 2007; Laflamme et al. 2007; Yang et al. 2008; Lian et al. 2013). In contrast, current knowledge of the intracellular mediators controlling this process is very fragmentary. Discovering such factors would have major implications (1) for appreciating how cardiogenesis is normally initiated, as embryos lacking cardiac progenitors fail to form a heart (Zhao et al. 2008), and (2) for informing the development of regenerative and disease modeling technologies (Mercola et al. 2013; Moretti et al. 2013).

Basic helix–loop–helix (bHLH) transcription factors *Mesp1* and *Mesp2* (Saga et al. 2000) under the control of T-box factor *Eomes* (Costello et al. 2011) regulate at least part of this process in mesoderm cells by directing the expression of genes involved in cardiac specification (*Hand2*, *Gata4*, *Nkx2.5*, and *Myocd*) and cellular migration (*Prickle1* and *RasGRP3*) while actively repressing genes regulating pluripotency (*Oct4*, *Nanog*, and *Sox2*) and early mesoderm (*T*) and endoderm (*Foxa2* and *Sox17*) fates (Bondue et al. 2008; Costello et al. 2011; Chiapparo et al. 2016). Although these observations suggest that *Mesp1/2* genes could act as master regulators of multipotent cardiovascular specification, retrospective lineage analysis (Saga et al. 2000; Yoshida et al. 2008) and in vitro differentiation studies (Chan et al. 2013) have shown that *Mesp1*-expressing cells also contribute to a wide range of noncardiac derivatives, including hematopoietic precursors, skeletal muscle cells, and head mesenchyme. Therefore, additional effectors responsible for specifying cardiac cell fate remain to be discovered.

We reported recently that attenuating Acvr1b signaling in mesendoderm segregates cardiogenic mesoderm from endoderm, whereas persistent Acvr1b signaling drives cells to form endoderm (Colas et al. 2012). Thus, we hypothesized that genes induced in response to Acvr1b signaling inhibition might be key determinants of cardiogenic mesoderm formation. We took a systematic and unbiased approach to functionally test the necessity and sufficiency of the genes modulated by Acvr1b signaling blockade. Unexpectedly, we first identified *Id1*, a HLH transcriptional regulator, as a single factor sufficient to control the emergence of Kdr^+^ CMPs both in mouse and human embryonic stem cells (mESCs and hESCs, respectively). Mechanistically, we discovered that Id proteins mediate their evolutionarily conserved role by activating the expression of agonists of cardiogenic mesoderm formation (*Evx1*, *Grrp1*, and *Mesp1)* while inhibiting antagonists’ activity (*Tcf3* and *Foxa2)*. Finally, CRISPR/Cas9-mediated deletion of all four *Id* family members in mouse embryos blocked early cardiac progenitor formation and yielded embryos without a heart. The heartless phenotype was unique to the quadruple knockout, indicating compensatory or redundant functions of the Id proteins in the formation of the cardiac mesoderm. These findings reveal an unexpected role for Id proteins as the earliest determinants of cardiac cell fate in vertebrates.

## Results

### Identification of new agonists of cardiogenic mesoderm formation

**Figure 1.**
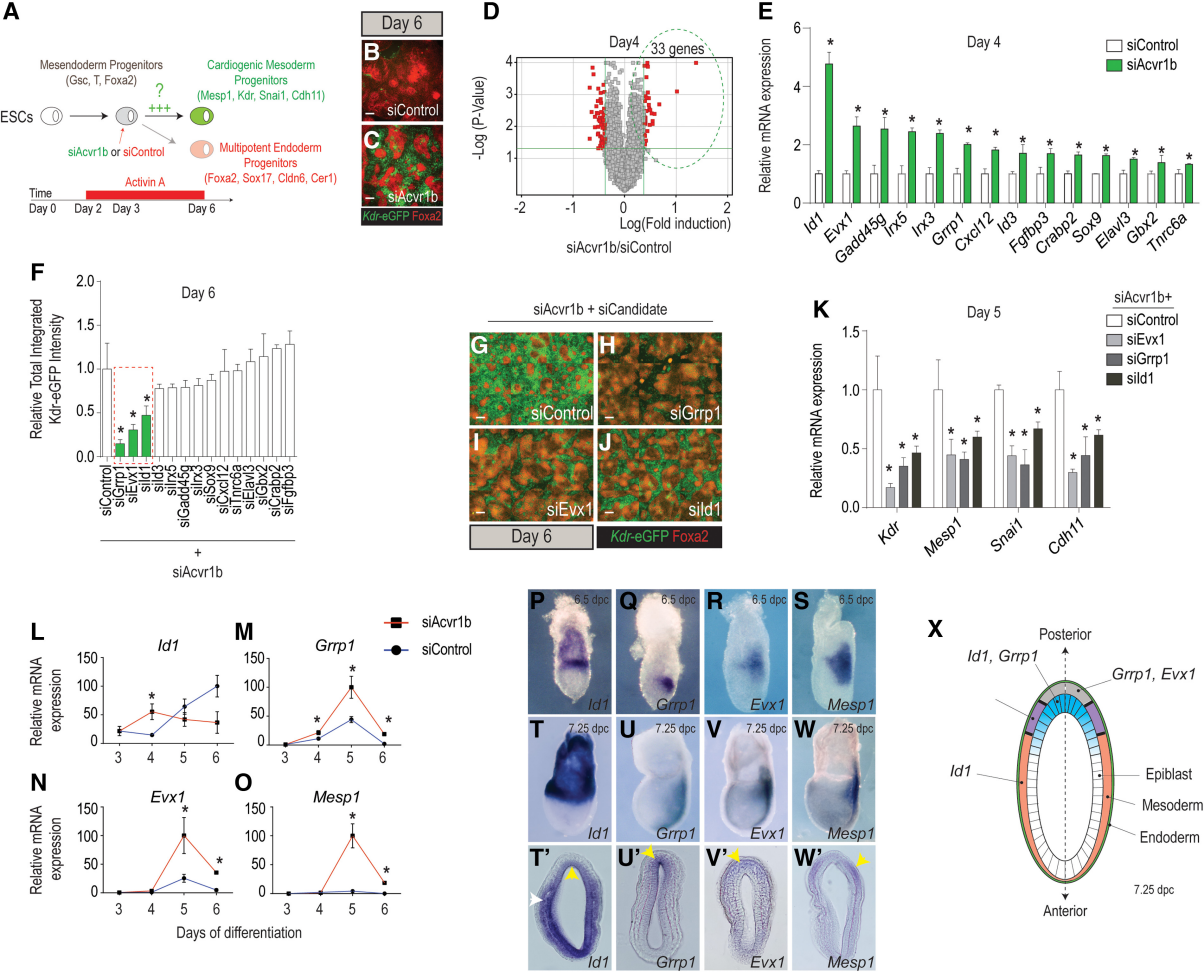
Positive regulators of CMP formation. (*A*) Schematic of screening strategy to identify new regulators of cardiogenic mesoderm differentiation. (*B*,*C*) Immunostaining of *Kdr*-eGFP (cardiogenic mesoderm) and Alexa fuor568-Foxa2 (endoderm) showing increased mesoderm differentiation in response to siAcvr1b as compared with siControl at day 6 of differentiation. Bar, 50 µm. (*D*) Microarray data reveal that 33 transcripts are up-regulated (*P* < 0.05) at day 4 in response to siAcvr1b as compared with siControl 24 h after transfection. (*E*) Quantitative RT–PCR (qRT–PCR) confirmation of the microarray results in *D*, showing that 14 genes are robustly up-regulated in response to siAcvr1b as compared with siControl. (*F*) siRNA screen of the 14 candidates from *E* to evaluate their requirement for cardiogenic mesoderm formation induced by siAcvr1b. Differentiation was quantified by induction of *Kdr*-eGFP reporter (total integrated intensity) (see the Materials and Methods for details). siGrrp1, siEvx1, and siId1 strongly repressed siAcvr1b-induced cardiogenic mesoderm. (*G*–*J*) Representative images of *Kdr*-eGFP and Alexa fluor 568-Foxa2 illustrating results presented in *F*. Bar, 50 µm. (*K*) qRT–PCR results showing that siGrrp1, siEvx1, and siId1 markedly repress cardiogenic mesoderm-specific marker (*Kdr*, *Mesp1*, *Snai1*, and *Cdh11*) expression. (*L*–*O*) Temporal expression profiles of *Id1*, *Grrp1*, *Evx1*, and *Mesp1* in response to siAcvr1b or siControl from day 3 to day 6 of differentiation. (*P*–*W*′) Endogenous expression of *Id1*, *Grrp1*, *Evx1*, and *Mesp*1 in embryonic day 6.5 (E6.5) and E7.25 mouse embryos by in situ hybridization. (*P*–*W*) Whole-mount view. Transverse histological section of the proximal region of E7 embryos indicating *Id1* (*T*′) expression in the gastrulating epiblast (yellow arrow) and migrating mesoderm (white arrow), *Grrp1* (*U*′) expression in the gastrulating epiblast (yellow arrow), and *Evx1* (*V*′) and *Mesp1* (*W*′) expression in the primitive streak (yellow arrow). (*X*) Schematic representation of an E7.25 embryo transverse section illustrating the different domains of expression of the three candidates. The gastrulating epiblast (blue) indicates the domain where *Id1* and *Grrp1* expression overlaps. In the primitive streak region (gray), high levels of *Evx1* expression are observed with decreased *Grrp1* expression. As cells exit the primitive streak and migrate laterally (purple), they start to express *Mesp1* along with *Evx1*. As mesoderm cells migrate more anteriorly (orange), they resume *Id1* expression. All qRT–PCR data were normalized to β*-actin* mRNA levels. Quantitative data are presented as means ± SD. (*) *P* < 0.05. All experiments were performed at least in biological quadruplicates.

mESCs form mesendodermal progenitors (Gsc^+^, Foxa2^+^, and T^+^) at days 3–4 of differentiation in response to Activin/Nodal signaling and subsequently differentiate into either Foxa2^+^ definitive endoderm or Kdr^+^ cardiogenic mesoderm (diagrammed in Fig. 1A; described previously in Colas et al. 2012). Attenuation of Acvr1b drives mesendodermal progenitors to form CMPs marked by Mesp1, Kdr, Cdh11, and Snai1 expression at days 5–6 rather than endoderm—a process robustly elicited by transfecting mesendodermal progenitors at day 3 with either *let-7* or *miR-18* mimics or siRNAs directed against their respective mRNA targets: *Acvr1b* or *Smad2* (day 3) (Fig. 1A–C; Supplemental Movies S1–2; Colas et al. 2012; McKeithan et al. 2012).

To identify the downstream effectors of cardiogenic mesoderm formation, we analyzed mRNA expression 24 h after *Acvr1b* siRNA (siAcvr1b) transfection (day 4). Microarray data revealed 33 genes that were up-regulated (Fig. 1D; Supplemental Table S1) in response to *Acvr1b* siRNA relative to a scrambled sequence siRNA control, of which 14 were confirmed by quantitative PCR (qPCR) (Fig. 1E). Consistent with a potential role as cell fate regulators, eight of the candidate genes are known regulators of gene transcription, including transcription factors (*Evx1*, *Gbx2*, *Irx3*, *Irx5*, and *Sox9*), inhibitors of bHLH transcription factors (*Id1* and *Id3*), and a mediator of DNA demethylation (*Gadd45g*). Of the six remaining candidates, three are signaling pathway modulators (*Fgfbp3*, *Crabp2*, and *Cxcl12*), two are involved in RNA processing (*Elavl3* and *Tnrc6a*), and one encodes a protein with two centrosome-associated domains but no known function (*Grrp1*). Interestingly, none of the 14 candidates were shown previously to directly control cardiogenic mesoderm formation, suggesting that we identified a novel molecular signature marking differentiating CMPs.

Next, we assessed whether siRNA against each of the 14 candidates would block cardiogenic mesoderm formation induced by siAcvr1b using a *Kdr*-eGFP reporter system (Colas et al. 2012). Of all of the up-regulated genes, only siRNAs against *Grrp1*, *Evx1*, and *Id1* significantly decreased the number of Kdr^+^-expressing cells (Fig. 1F–J) and blunted the induction of cardiogenic mesoderm marker genes, including *Kdr*, *Mesp1*, *Snai1*, and *Cdh11* (Fig. 1K). Thus, *Grrp1*, *Evx*1, and *Id1* are needed for normal cardiogenic mesoderm differentiation in mESCs.

### Spatiotemporal expression of *Id1*, *Grrp1*, and *Evx1* is consistent with involvement in cardiogenic mesoderm formation

In siAcvr1b conditions, maximal *Id1* expression occurs at day 4 of mESC differentiation, preceding the peaks of *Grrp1*, *Evx1*, and *Mesp1* expression (Fig. 1L-O). In mice, *Id1* is expressed throughout the epiblast in the most proximal region of gastrula stage embryos (embryonic days 6.5–7.25 [E6.5–E7.25]) and the anterior lateral mesoderm where specified cardiac precursors are located (Fig. 1P,T,T′; Devine et al. 2014). *Id1* transcripts are notably absent from the primitive streak, posterior mesoderm, and definitive endoderm. *Grrp1* transcripts are expressed throughout the primitive streak of the embryo (Fig. 1Q,U,U′). Transverse sections reveal that *Grrp1* mRNA is mostly localized in the gastrulating epiblast and rapidly declines as cells migrate away from the primitive streak. Like *Id1*, *Evx1* is expressed in the most proximal region of the embryo within the primitive streak and migrating mesoderm (Fig. 1R,V,V′). *Mesp1* expression marks the early differentiating multipotent mesoderm cells as they emerge from the primitive streak and start to migrate (Fig. 1S,W,W′). Thus, the spatiotemporal expression of candidate transcripts is consistent with their potential involvement in cardiogenic specification in mesendoderm progenitors that normally takes place between E6.5 and E7.5 (Bardot et al. 2017). The data also suggest that *Id1* in the gastrulating epiblast may function upstream of *Grrp1* and *Evx1* to ultimately direct *Mesp1* expression in cells that exit the primitive streak (Fig. 1X).

### Id1 is sufficient to direct Kdr^+^ cardiogenic mesoderm formation in mESCs and hESCs

**Figure 2.**
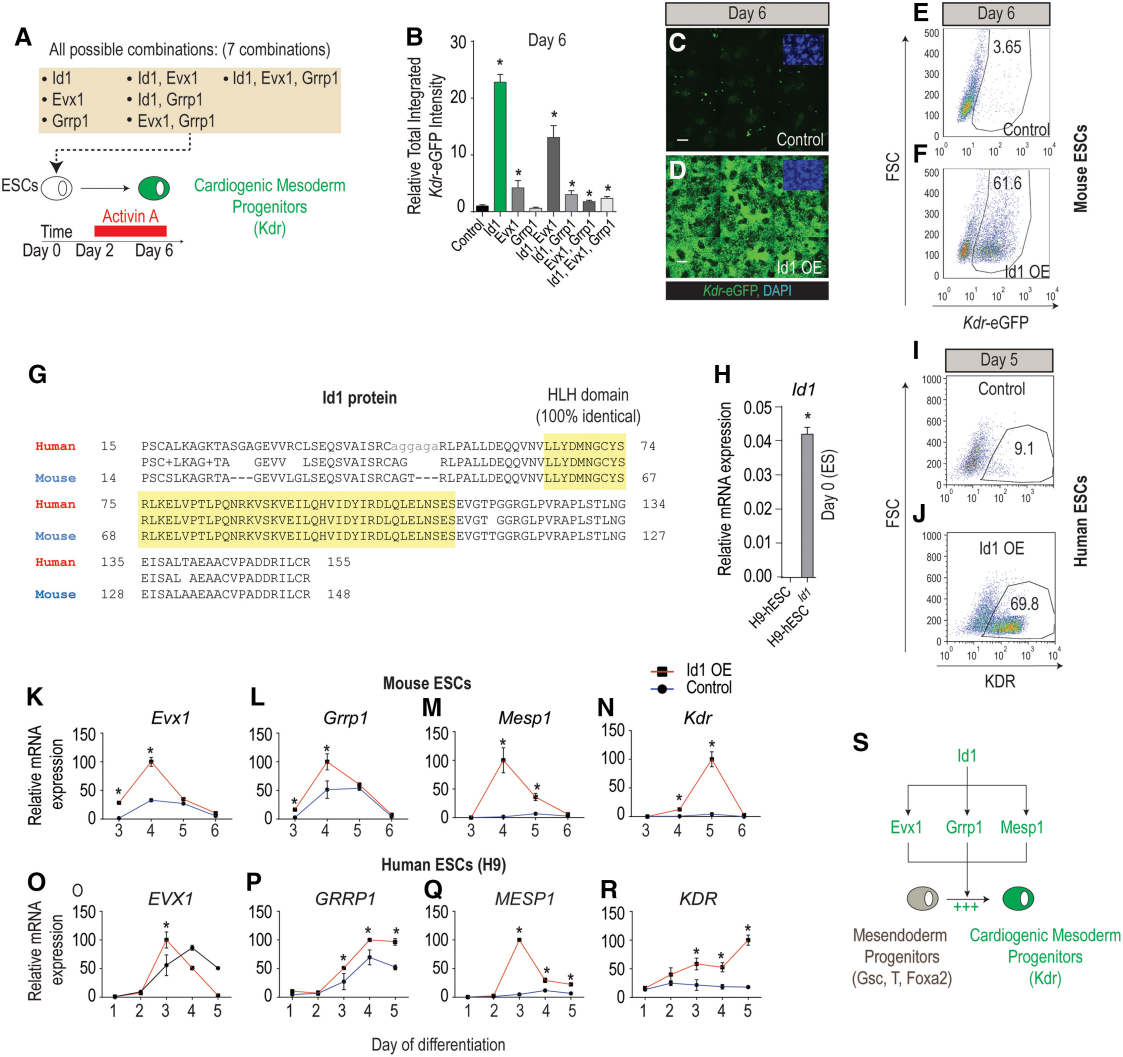
Id1 is sufficient to direct Kdr^+^ mesoderm formation in mESCs and hESCs. (*A*) Schematic of the strategy to evaluate the sufficiency (gain of function) of any of three candidates alone or in combination to promote mesoderm differentiation. (*B*) *Kdr*-eGFP fluorescence measurement at day 6 of differentiation in mESCs overexpressing all possible combinations of the three candidates plotted relative to uninfected control levels. (*C*,*D*) Representative images of *Kdr*-eGFP for Id1-overexpressing versus control mESCs illustrating the results presented in *B*. Bar, 50 µm. (*E*,*F*) Flow cytometry analysis reveals that 61.6% of *Id1*-overexpressing mESCs differentiate into *Kdr*-eGFP^+^ mesoderm as compared with 3.65% for control cells at day 6. (*G*) Alignment and comparison of the mouse (NP_034625.1) Id1 HLH domain and the human (NP_851998.1) Id1 HLH domain using the Protein Blast tool (https://blast.ncbi.nlm.nih.gov) reveals that the amino acid sequence is 100% identical. (*H*) qRT–PCR analysis for expression of *Id1* in control h9 hESCs versus h9 hESCs stably overexpressing Id1 measured at day 0 of differentiation. (*I*,*J*) Flow cytometry analysis reveals that 69.8% of Id1-overexpressing h9 hESCs differentiate into KDR^+^ mesoderm at day 5 of differentiation as compared with 9.1% for control h9 hESCs. (*K*–*N*) Temporal mRNA expression profile of procardiogenic mesoderm genes (*Evx1* [*K*], *Grrp1* [*L*], *Mesp1* [*M*], and *Kdr* [*N*]) in mESC lines overexpressing Id1 compared with control mESC lines illustrating that *Evx1*, *Grrp1*, and *Mesp1* mRNA expression peaks at day 4 of differentiation, while *Kdr* mRNA expression peaks at day 5 of differentiation. (*O*–*R*) Temporal mRNA expression profiles of *EVX1* (*O*), *GRRP1* (*P*), *MESP1* (*Q*), and *KDR* (*R*) in h9 hESCs stably overexpressing Id1 compared with control h9 hESCs. (*S*) Model summarizing the procardiogenic role of *Id1* by up-regulating the expression of *Evx1*, *Grrp1*, and *Mesp1* in bipotent mesendoderm progenitors. Quantitative data are presented as means ± SD. All experiments were performed at least in biological quadruplicates. The *insets* in the *top right* corners of all immunostaining images show corresponding DAPI staining.

To evaluate whether candidate genes alone or in combination are sufficient to promote cardiogenic mesoderm differentiation, we first generated mESC lines overexpressing all seven possible combinations of the three candidates (Fig. 2A; Supplemental Fig. S1A). The cell lines were treated with Activin A (but not with *Acvr1b* siRNA), and the resulting differentiation was assessed on day 6. Id1 was sufficient to massively direct ESCs to differentiate toward the Kdr^+^ mesoderm without attenuating Acvr1b expression (∼22-fold over parental mESCs), while the other genes had less potent effects (Fig. 2B–D; Supplemental Fig. S1B–G). Quantitatively, the conversion rate of Id1-overexpressing mESCs into *Kdr*-eGFP^+^ mesoderm was ∼60% as compared with only 3.65% for control ESCs (Fig. 2E,F).

Next, we asked whether Id1 functions similarly in hESCs by generating a WiCell (H9) hESC line that stably overexpresses mouse Id1, since the HLH domains are identical across these species (Fig. 2G,H). Consistent with the result with mESCs, Id1 greatly increased the incidence of KDR^+^ mesoderm in Activin A-treated cultures at day 5 from 9.1% in parental hESCs to 69.8% in hESC^Id1^ (Fig. 2I,J).

Remarkably, the formation of Id1-induced Kdr^+^/KDR^+^ mesoderm progenitors (iMPs) was consistently preceded by the up-regulation of *Evx1/EVX1* and *Grrp1/GRRP1* (days 3/4 in mESCs [Fig. 2K,L] and day 3 in hESCs [Fig. 2O,P]) followed by dramatic *Mesp1/MESP1* up-regulation (∼67-fold in mESCs at day 4 and ∼20-fold in hESCs at day 3) (Fig. 2M,Q) and subsequent *Kdr*/*KDR* up-regulation at day 4 and day 5, respectively (Fig. 2N,R). Together, these data show that Id1 initiates an evolutionarily conserved gene regulatory network (*Evx1/EVX1*, *Grrp1/GRRP1*, and *Mesp1/MESP1*) controlling the formation of Kdr^+^/KDR^+^ mesoderm (Fig. 2S).

**Figure 3.**
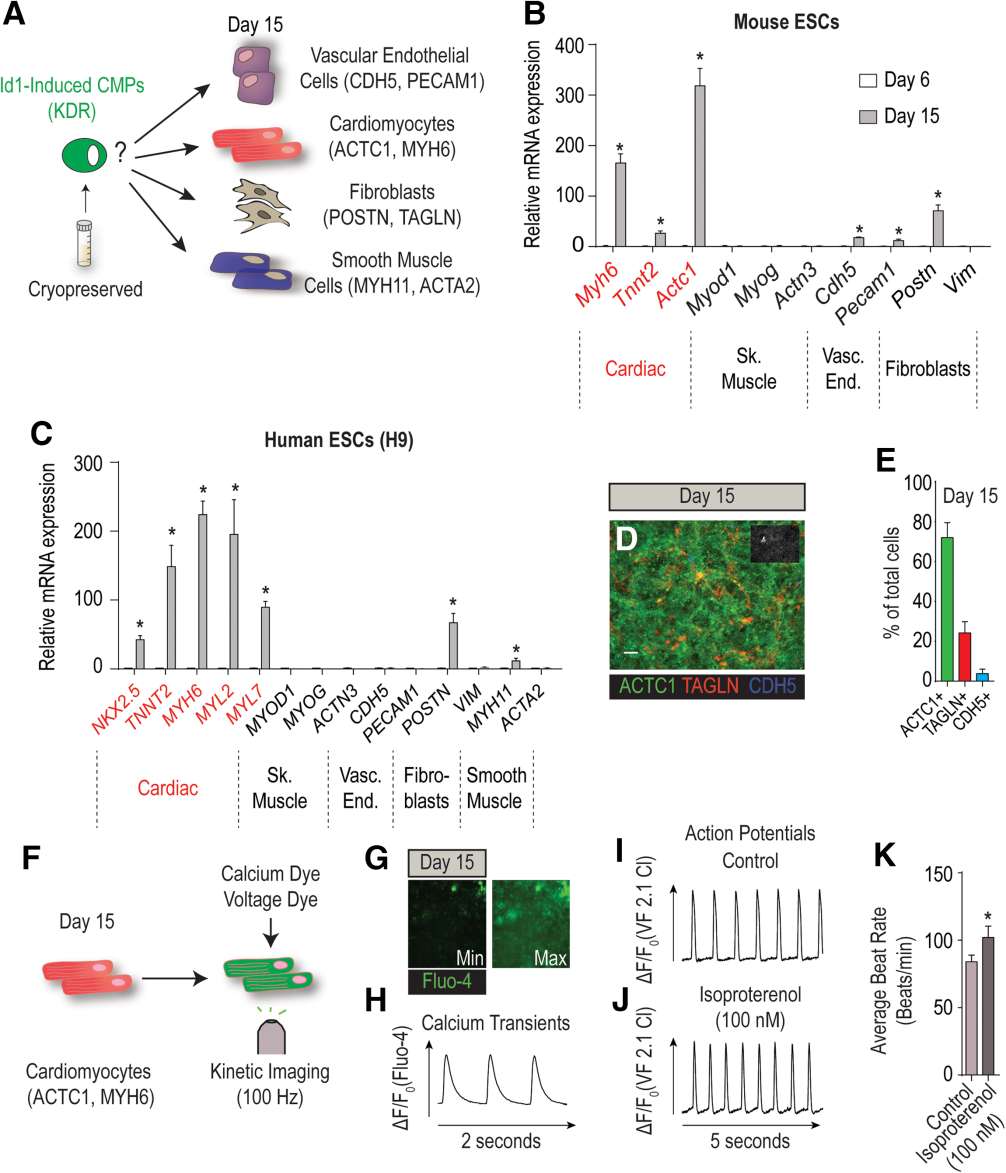
Id1-induced Kdr^+^ mesoderm is cardiogenic. (*A*) Schematic depicting the prospective differentiation potential of cryopreserved Id1-induced CMPs to multiple cardiovascular cell types. (*B*) mRNA expression profiling for the spontaneous differentiation potential of mESCs stably overexpressing *Id1* to cardiac (*Myh6*, *Tnnt2*, and *Actc1*), skeletal muscle (*Myod1*, Myog, and Actn3), vascular endothelial (*Cdh5* and *Pecam1*), and fibroblast (*Postn* and *Vim*) markers at days 6 and 15 of differentiation. (*C*) mRNA expression profiling for the spontaneous differentiation potential of h9 hESCs stably overexpressing *Id1* to cardiac (*NKX2.*5, *TNNT2*, *MYH6*, *MYL2*, and *MYL7*), skeletal muscle (*MYOD1*, *MYOG*, and *ACTN3*), vascular endothelial (*CDH5* and *PECAM1*), smooth muscle (*MYH11* and *ACTA2*), and fibroblast (*POSTN* and *VIM*) markers at days 5 and 15 of differentiation. (*D*) Representative immunofluorescence image of cardiomyocytes (ACTC1), vascular endothelial cells (CDH5), and fibroblasts (TAGLN) at day 15 of differentiation in h9 hESCs stably overexpressing *Id1*. Bar, 50 µm. (*E*) Diagram showing quantification of the percentage of ACTC1^+^ (cardiomyocytes), TAGLN^+^ (fibroblasts), and CDH5^+^ (vascular endothelial cells) at day 15 of differentiation in h9 hESCs stably overexpressing *Id1*. (*F*) Schematic of the work flow for the physiological assessment of cardiomyocytes derived from *Id1-*overexpressing h9 hESCs using the calcium-sensitive (Fluo-4) and voltage-sensitive (VF2.1 Cl) (Miller et al. 2012) dyes. (*G*) Representative images illustrating the minimum and maximum changes in fluorescence of Fluo-4 in cardiomyocytes derived from *Id1*-overexpressing h9 hESCs. (*H*) Representative calcium transient trace of day 15 cardiomyocytes derived from *Id1*-overexpressing h9 hESCs. (*I*,*J*) Representative action potential traces of cardiomyocytes derived from *Id1*-overexpressing h9 hESCs in control conditions (*I*) or in response to isoproterenol (*J*) measured optically with VF2.1 Cl. (*K*) Beat rate quantification of cardiomyocytes derived from *Id1*-overexpressing h9 hESCs indicating an increase in beating frequency in response to 100 nM isoproterenol treatment as compared with vehicle and measured with VF2.1 Cl. Quantitative data are presented as means ± SD. All experiments were performed at least in biological quadruplicates. The *insets* in the *top right* corners of all immunostaining images shows corresponding DAPI staining.

Next, we asked whether iMPs are bona fide CMPs and thus are able to differentiate into multiple cardiac lineages, including functional cardiomyocytes. To address this question, iMPs were first produced in bulk until day 6 of differentiation for mice or day 5 for humans. At this point, iMPs could be cryopreserved or used fresh. Spontaneous differentiation potential under basal medium conditions without cytokines (Fig. 3A) was assessed by RT-qPCR (Fig. 3B,C) and immunostaining (Fig. 3D; Supplemental Fig. S2A,B) at day 15 of differentiation. The results showed that iMPs spontaneously differentiate into at least four distinct cellular lineages normally present in the heart, including cardiomyocytes (Myh6, Tnnt2, and Actc1), vascular endothelial cells (Pecam1 and Cdh5), smooth muscle (Myh11), and fibroblasts (Postn and Tagln). Although iMPs are multipotent progenitors, the vast majority of the cells (∼70%) spontaneously differentiated into ACTC1^+^ cardiomyocytes in hESCs (Fig. 3E). Next, we assessed whether the resulting ACTC1^+^ cells show characteristics of functional cardiomyocytes, which include the rhythmic contractions, intracellular calcium oscillations and action potentials, and response to hormonal stimuli (Burridge et al. 2014; Birket et al. 2015). High-speed optical recording (100 frames per second) (Fig. 3F) revealed that day 15 iMPs contracted rhythmically (Supplemental Movie S3), displayed periodic calcium transients (Fig. 3G,H; Supplemental Movie S4) and action potentials (Fig. 3I; Supplemental Movie S5), and showed increased beat rate in response to the β-adrenergic agonist isoproterenol (Fig. 3J,K; Supplemental Movie S6). In summary, these observations demonstrate that iMPs represent a novel population of bona fide CMPs with a remarkable ability to spontaneously differentiate into functional cardiomyocytes.

### Id1 promotes cardiogenic mesoderm differentiation by inhibiting Tcf3 and Foxa2

**Figure 4.**
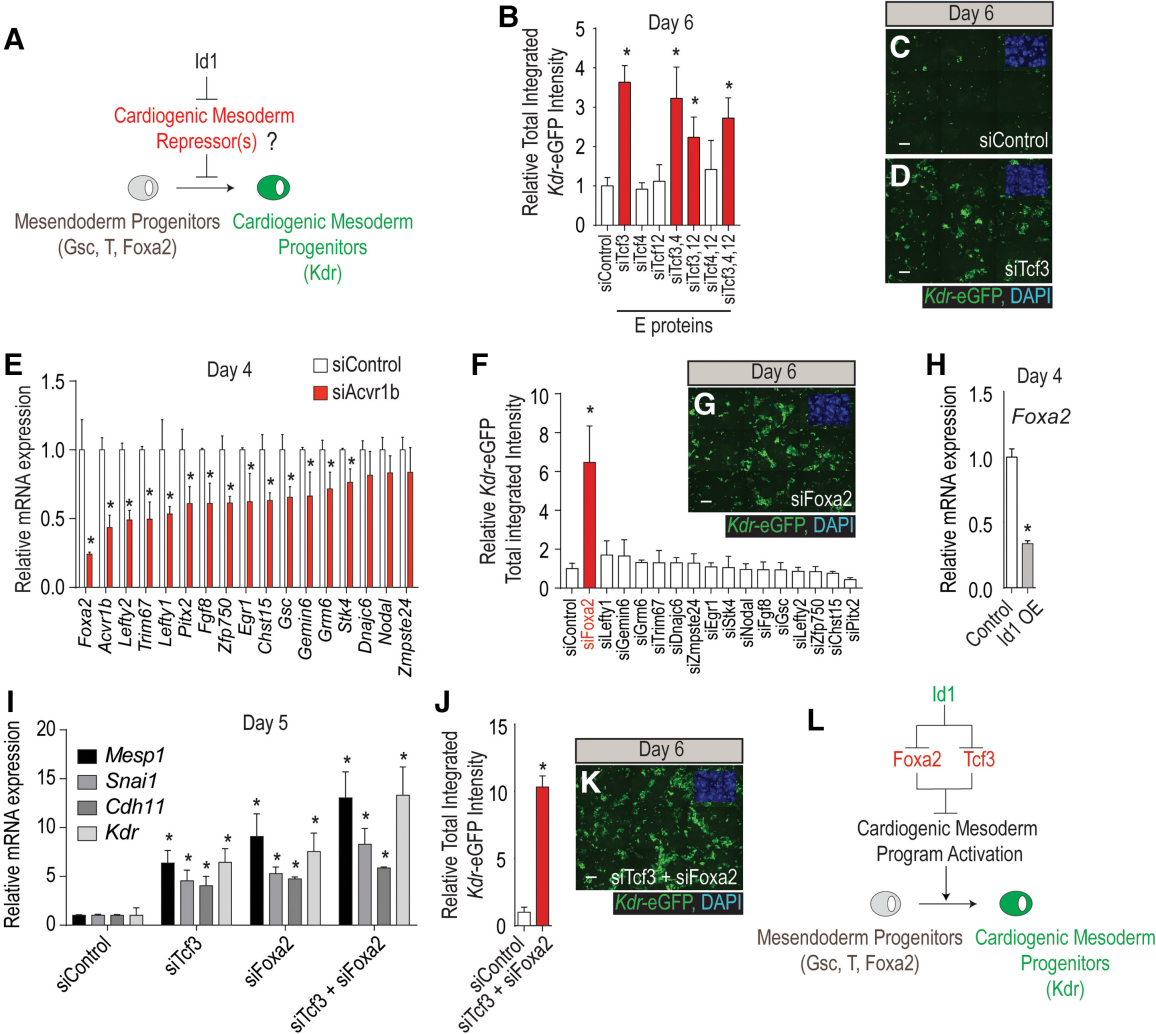
Id1 promotes cardiogenic mesoderm differentiation by inhibiting Tcf3 and Foxa2. (*A*) Schematic predicting that *Id1* mediates its procardiogenic effect by targeting and inhibiting repressors of cardiogenic mesoderm differentiation. (*B*) siRNA-mediated functional screen evaluating the role of E proteins (*Tcf3*, *Tcf4*, and *Tcf12*) in repressing cardiogenic mesoderm differentiation. The diagram shows the fluorescence quantification of *Kdr*-eGFP in response to all seven possible siRNA combinations and siControl. (*C*,*D*) Representative immunofluorescence images of *Kdr*-eGFP at day 6 of differentiation from mESCs transfected at day 3 with siControl (*C*) and siTcf3 (*D*). Bar, 50 µm. (*E*) qRT–PCR validation showing that 17 genes are down-regulated at day 4 in response to siAcvr1b as compared with siControl 24 h after transfection. (*F*,*G*) siRNA-mediated functional screen evaluating whether downstream targets of Acvr1b signaling are involved in the repression of cardiogenic mesoderm differentiation. (*F*) The diagram shows the fluorescence quantification of *Kdr*-eGFP, where only a siRNA directed against siFoxa2 is able to promote cardiogenic mesoderm differentiation. (*G*) Representative *Kdr*-eGFP immunofluorescence images of siFoxa2. Bar, 50 µm. (*H*) qRT–PCR shows that *Foxa2* expression is down-regulated in *Id1*-overexpressing mESCs as compared with control. (*I*–*K*) qRT–PCR for cardiogenic mesoderm markers (*Mesp1*, *Snai1, Cdh11*, and *Kdr*) showing that the cotransfection of siFoxa2 and siTcf3 further enhances cardiogenic mesoderm differentiation as compared with siTcf3 or siFoxa2 alone (shown in *I*). The diagram shows the fluorescence quantification of *Kdr*-eGFP (*J*) and a representative image (*K*) of the siTcf3 + siFoxa2 condition. Bar, 50 µm. (*L*) Model showing Id1's repressive role on Tcf3 and Foxa2 activity to promote cardiogenic mesoderm differentiation.

Id proteins do not directly bind DNA but regulate transcription by antagonizing the function of bHLH transcription factors through their HLH domains (Kee 2009). Their canonical partners are class I bHLH transcription factors (E proteins) Tcf3, Tcf4, and Tcf12 (Kee 2009; Yang et al. 2014). Therefore, to determine whether Id1 might initiate cardiogenic mesoderm formation by inhibiting E proteins (Fig. 4A), we tested whether siRNAs directed against the three E proteins either alone or in combination (seven combinations) would promote *Kdr*-eGFP fluorescence at day 6 of differentiation as above. All combinations of siRNAs that contained siTcf3 promoted cardiogenic mesoderm differentiation (approximately fourfold over siControl) (Fig. 4B–D). Although these studies implicate Tcf3 as a relevant target of Id1, siTcf3 was significantly less potent than either Id1 overexpression or siAcvr1b transfection, suggesting that additional targets are involved in inducing *Kdr-eGFP*^*+*^ cells. Therefore, we screened all 104 members of the class II family of bHLH transcription factors (e.g., MyoD, NeuroD, myogenin, etc.) by an analogous approach, but none had any effect on cardiogenic mesoderm formation (data not shown).

Next, we tested whether Id1 might mediate part of its procardiogenic mesoderm activity by down-regulating antagonists of cardiogenic mesoderm formation. Such genes should be among those down-regulated in response to the procardiogenic mesoderm actions of siAcvr1b at day 4 of differentiation. Of the 53 genes identified in the microarray (Supplemental Table S1), 17 were confirmed by RT-qPCR to be robustly down-regulated by siAcvr1b (Fig. 4E). Next, we tested whether siRNA-mediated knockdown of any of these 17 genes would be sufficient to promote *Kdr*-eGFP^+^ cardiogenic mesoderm formation. Strikingly, siRNA to only one gene, encoding the forkhead transcription factor *Foxa2*, was sufficient to induce *Kdr*-eGFP^+^ mesoderm (Fig. 4F,G). Although Id1 is not known to physically interact with forkhead transcription factors, overexpression of Id1 strongly decreased the abundance of *Foxa2* transcripts in the cells relative to controls (Fig. 4H), suggesting that Id1 indirectly inhibits *Foxa2* gene expression.

Consistent with our hypothesis, *Tcf3* and *Foxa2* knockdowns (Supplemental Fig. S3A,B) derepressed cardiogenic mesoderm gene expression (*Mesp1*, *Snai1*, *Cdh11,* and *Kdr)* (Fig. 4I), and the combined knockdown of *Tcf3* and *Foxa2* further enhanced cardiogenic mesoderm differentiation efficiency, suggesting that both genes might act in a nonredundant manner during this process (Fig. 4I–K). Finally and in accordance with our molecular model, *Tcf3* transcripts are ubiquitously expressed and overlap with *Id1* in the most proximal region of the embryo at E6.5, while, in contrast, *Foxa2* transcripts are localized in the anterior primitive streak in a domain that is more distal and nonoverlapping with *Id1* (Supplemental Fig. S3C,D). We conclude that Id1 activates the cardiogenic program by inhibiting Tcf3 protein activity while suppressing *Foxa2* transcription (Fig. 4L).

### Id proteins promote cardiogenic mesoderm formation in vivo

**Figure 5.**
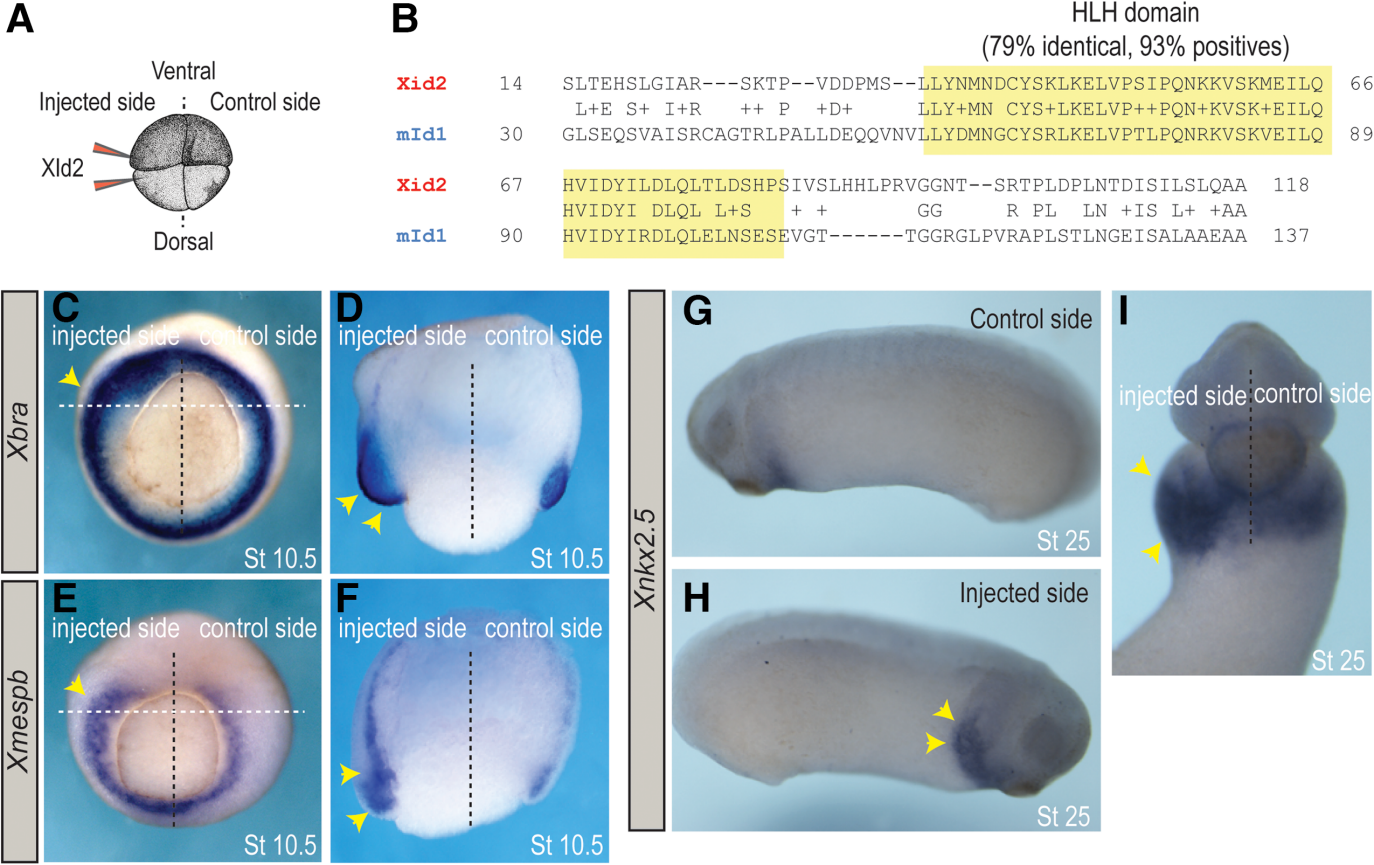
Id proteins promote cardiogenic mesoderm formation in vivo. (*A*) *Xid2* mRNA was injected equatorially into two blastomeres on one side of four-cell stage embryos. (*B*) The mouse HLH domain of Id1 (NP_034625.1) was aligned and compared with all *Xenopus laevis* HLH (yellow) domains of id proteins using the Protein Blast tool (https://blast.ncbi.nlm.nih.gov). With 79% of identical amino acids, Xid2 (NP_001081902.1) (*A*) is the closest ortholog to Id1. (*C*–*F*) Unilaterally injected embryos (as in *A*) cultured to gastrula stage (stage 10.5) in whole mount (*C*,*E*) or transversely bisected (*D*,*F*) and probed for mesoderm marker *Xbra* (*C*,*D*) and cardiogenic mesoderm *Xmespb* (*E*,*F*) expression. Yellow arrowheads indicate expansions of both the *Xbra* and *Xmespb* domains in the *Xid1*-injected side. (*G*–*I*) Unilaterally injected embryos cultured to early tail bud stage (stage 25) in whole mount and probed for *Xnkx2.5* expression. Yellow arrowheads indicate an expansion of the *Xnkx2.5* domain in the *Xid2*-injected side of the embryo.

Next, we used *Xenopus* embryos to test whether *Id* genes can promote cardiogenic mesoderm formation in vivo. Equatorial and hemilateral injection of *Xid2* mRNA (Fig. 5A), which is the closest ortholog to mouse *Id1* (Fig. 5B), caused a dramatic expansion of *Xbra* (74%, *n* = 105) (Fig. 5C,E) and *Xmespb* (78%, *n* = 132) *(*Fig. 5D,F) expression domains (marking mesoderm) in gastrula stage embryos (Nieuwkoop and Faber stage 10.5). To determine whether an expanded *Xbra*^+^, *Xmespb*^+^, domain marks an increase in cardiogenic mesoderm formation, *Xnkx2.5* expression was examined at tail bud stage (stage 25). Strikingly, *Xid2* overexpression caused an expansion of *Xnkx2.5* expression (68%, *n* = 88) (Fig. 5G–I), while, in contrast, noncardiac mesoderm such as skeletal muscle (*Xmlc*) was not expanded (66%, *n* = 30) (Supplemental Fig. S4A–C). Taken together, these data indicate that Xid2, like mammalian Id1 in ESCs, specifically promotes the formation of mesoderm progenitors that are primed to differentiate toward cardiac lineages rather than other mesoderm lineages.

### Id genes are essential for early mammalian heart formation in vivo

**Figure 6.**
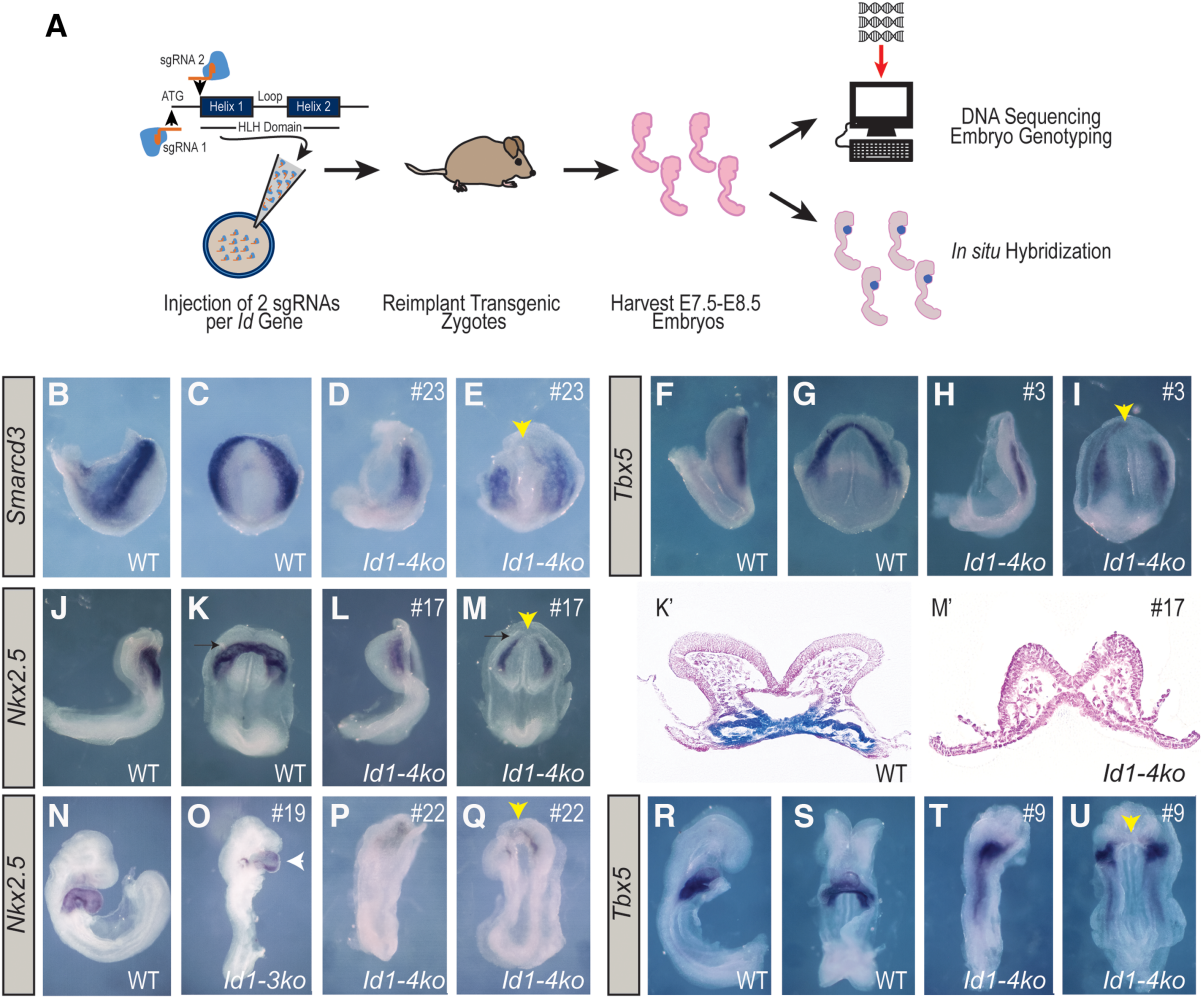
Id genes are essential for early heart formation. (*A*) Schematic illustrating the generation and analysis of *Id1–4* mutant embryos using CRISPR/Cas9 technology. Two sgRNAs per gene (targeting the translational start site and the HLH domain) were injected into single-cell mouse zygotes alongside *Cas9* mRNA. Zygotes were reimplanted and harvested at stages E7.5–E8.5. Resulting embryos were genotyped by DNA deep sequencing, and cardiac gene expression was assessed via whole-mount in situ hybridization. (*B*–*U*) In situ hybridization results from the most severe *Id1–4* mutants—compared with wild type (individual mutants are marked by #)—plus one less-affected mutant (*O*); analysis of *Smarcd3* at E7.75 (*B–E*), *Tbx5* at E8.0 (*F*–*I*), *Nkx2*.5 at E8.25 (*J*–*M*; plus transverse sections through the heart tube-forming region [*K*′, *M*′]), *Nkx2*.5 at E8.5 (*N*–*Q*), and *Tbx5* at E8.5 (*R*–*U*). (Yellow arrowheads) Missing heart tube (or missing heart tube-forming region at cardiac crescent stages) in *Id1–4* mutants; (white arrowhead) malformed heart tube; (black arrows) the plane of transverse sectioning through the heart tube-forming region; (black dashed arrows) posterior–lateral cardiac regions. See the Supplemental Material for detailed sequencing results of mutant embryos.

Our gain-of-function experiments show that Id proteins are sufficient to direct the formation of cardiac mesoderm both in vitro and in vivo, so we next asked whether Id proteins are normally required for this process. A previous study (Fraidenraich et al. 2004) found that deleting three out of the four *Id* genes (*Id1,2,3* triple gene knockout) caused complex cardiac defects but did not ablate the heart in these embryos, thereby indicating that earlier cardiac specification could still occur. Given the functional similarity of Id family members (Lyden et al. 1999; Fraidenraich et al. 2004; Kee and Bronner-Fraser 2005; Niola et al. 2012, 2013), we reasoned that either redundant or compensatory activity of Id4 might allow heart formation to occur in triple-knockout embryos. To test this hypothesis, we genetically ablated all four *Id* gene members using a CRISPR/Cas9 genome-editing strategy in mouse embryos. To increase the probability of null allele generation, each *Id* gene was targeted by two single-guide RNAs (sgRNAs) directed against the ATG and the beginning of the HLH domain, respectively. Twenty-four embryos (ranging from E7.75 to E8.75) collected from three independent zygote injection sessions were subjected to genotyping by DNA sequencing and cardiac phenotype assessment by in situ hybridization (Fig. 6A). DNA sequencing results show that despite widespread mosaicism, 320 (90.7%) of the 353 alleles detected were null (elimination of the HLH domain reading frame), 24 (6.8%) were in-frame mutations, and only nine (2.5%) were wild type. Only seven out of 24 embryos harbored one or more wild-type alleles, while 17 out of 24 embryos harbored no wild-type alleles (Supplemental Fig. S5). Importantly, no off-target mutagenesis was detected in the top eight predicted off-target sites (see the Supplemental Material; http://crispr.mit.edu).

The phenotypic assessment at E7.75 showed that two markers of early cardiac precursors, *Smarcd3* and *Tbx5*, were absent from the most anterior and medial region of the cardiac crescent that gives rise to the heart tube (*n* = 9/11; embryos #21, #23, and #24 for *Smarcd3* and #1–#4, #7, #8, #10, and #13 for *Tbx5*) (Fig. 6B–I; Supplemental Fig. S6A–D; see Supplemental Fig. S5 for genotype information). In contrast, expression of these markers was maintained in two lateral domains of the mesoderm posterior to the heart tube-forming region, suggesting that these posterior cardiac progenitors could differentiate and migrate appropriately. At E8.25, when the heart tube has normally formed, in situ hybridization for the cardiac marker *Nkx2.5* revealed an absence of heart tube formation in *Id1–4* mutants (Fig. 6J–M; Supplemental Fig. S6E,F). Histological sectioning confirmed the absence of anatomical heart tube formation (Fig. 6K′,M′). At E8.75, when the heart begins to loop, analysis of *Nkx2.5* (Fig. 6O–Q) and the first heart field marker *Tbx5* (Fig. 6R–U) confirmed the absence of hearts in quadruple-knockout embryos (*n* = 10/13; embryos #14, #15, #17, and #22 for *Nkx2.5* and #5, #6, #9, #11, #12, and #13 for *Tbx5*) (Fig. 6L,M,P,Q,T,U). Finally and consistent with our initial hypothesis of functional redundancy between Id family members, embryos harboring at least one *Id4* wild-type allele can still form a heart tube that loops, albeit abnormally, as compared with controls (*n* = 3/13; embryos #18–#20) (Fig. 6N,O). Collectively, these results demonstrate that the *Id* family of genes is required for the specification of heart tube-forming CMPs and their subsequent assembly.

## Discussion

### Molecular control of cardiogenic mesoderm specification

**Figure 7.**
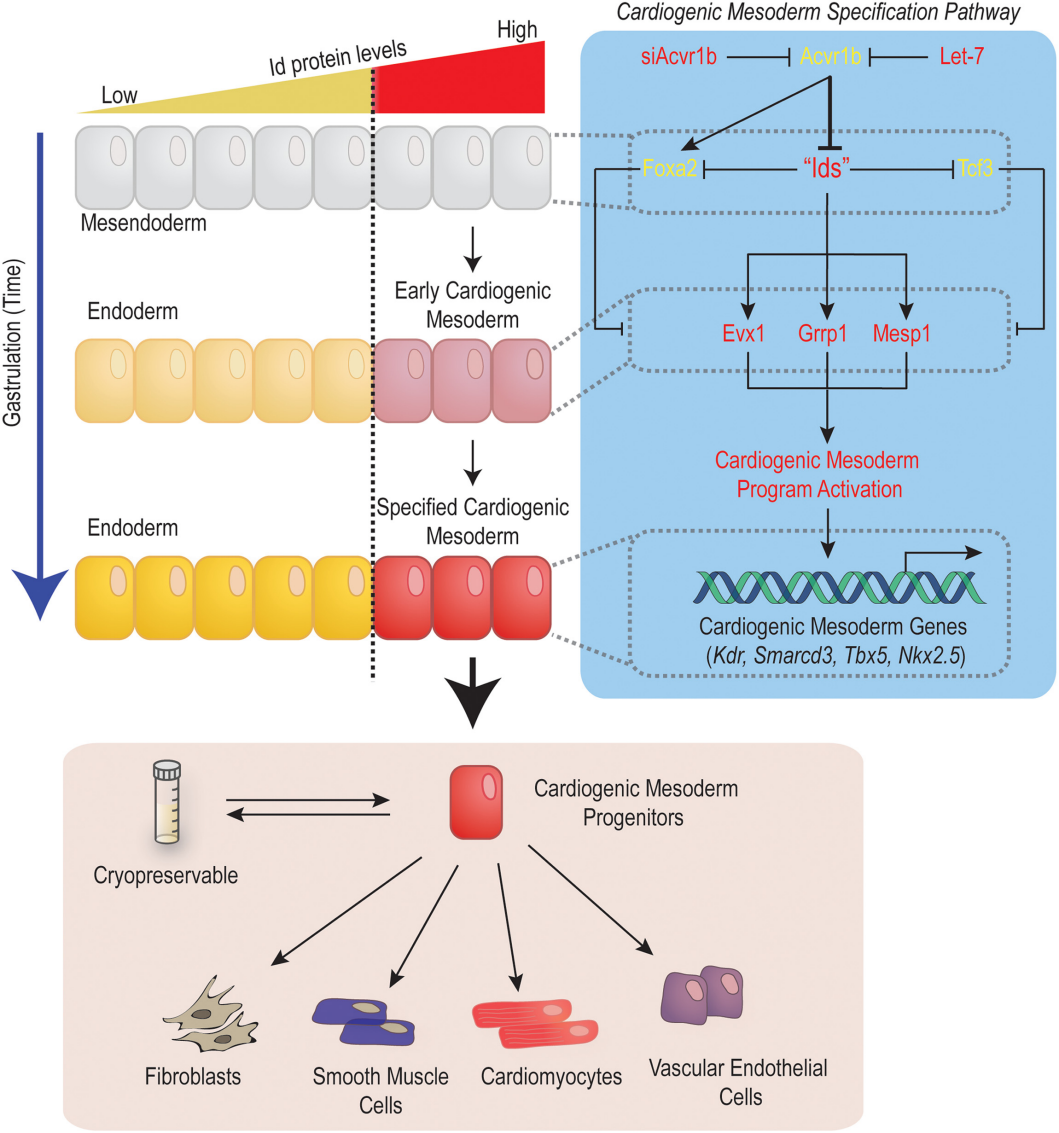
Id genes orchestrate cardiogenic mesoderm differentiation in vertebrates. *Id* genes control the activation of the cardiogenic mesoderm differentiation program in mesendoderm progenitors by inhibiting the activity of repressors (*Tcf3* and *Foxa2*) while promoting the expression of activators of cardiogenic mesoderm differentiation (*Evx1*, *Grrp1*, and *Mesp1*). The Id-controlled network induces cardiogenic mesoderm (*Mesp1* and *Kdr*) differentiation from pluripotent cells. Id1-induced CMPs generated from pluripotent stem cells are cryopreservable and spontaneously form contracting cardiomyocytes (∼70%) as well as vascular endothelial cells, smooth muscle, and fibroblasts.

Unraveling the molecular mechanisms controlling cardiogenic mesoderm specification is crucial for our understanding of how heart formation is normally initiated during embryonic development and for our ability to generate developmentally relevant cardiac progenitors for therapeutic and cardiac disease modeling purposes. This study reveals that cardiogenic mesoderm specification is tightly regulated in bipotent mesendoderm progenitors by an antagonistic interplay between Id proteins and the Acvr1b signaling pathway. High Acvr1b signaling represses the expression of *Id* genes and biases mesendoderm progenitors to differentiate toward endoderm. Conversely, attenuation of Acvr1b signaling in these cells derepresses *Id* gene transcription that in turn promotes cardiogenic mesoderm specification (Fig. 7).

A central finding of this study is the ability of Id proteins to override proendoderm cues (induced by high Acvr1b signaling) to promote cardiogenic mesoderm differentiation instead. The functional dominance of Id proteins over Acvr1b signaling also implies that molecules controlling the spatial and quantitative distribution of Id proteins are likely to be crucial regulators of cardiogenic mesoderm formation. Consistent with our model, BMP signaling directly activates *Id1* transcription (Hollnagel et al. 1999; Katagiri et al. 2002; Korchynskyi and ten Dijke 2002; Lopez-Rovira et al. 2002) and is both necessary and sufficient to induce cardiogenic mesoderm formation (Beppu et al. 2000; Yang et al. 2008; Paige et al. 2012). Conversely, the finding that Acvr1b signaling represses *Id1/3* gene expression is consistent with the ability of a small molecule inhibitor of the Nodal receptor (SB431542) to up-regulate *Id1* transcripts in mESCs (Galvin et al. 2010) and reinforces the role of Acvr1b signaling in opposing cardiac cell fate acquisition during gastrulation. In summary, high Id protein levels in mesendoderm progenitors constitute a dominant molecular cue that is sufficient to trigger and orchestrate cardiogenic mesoderm specification in vertebrates.

### Positioning *Id* genes in the context of *Mesp1* procardiogenic activity

Several transcription factors have been shown to be essential for cardiac development (Olson 2006; Bruneau 2013). Among them, *Mesp1* is the earliest expressed and is sufficient to directly promote cardiac specification in mesoderm progenitors (Saga et al. 1996; Bondue et al. 2008; David et al. 2008; Chan et al. 2013). Importantly, our gain-of-function experiments show that Id1/Xid2 is sufficient to direct *Mesp1/Xmespb* expression in both mESCs and hESCs as well as in *Xenopus* embryos and subsequently promote cardiogenic mesoderm differentiation. These observations suggest that Id proteins exert at least part of their procardiogenic effect by up-regulating *Mesp* genes. Since Id proteins do not directly bind DNA (Benezra et al. 1990; Roschger and Cabrele 2017), we propose that the up-regulation of *Mesp* genes is likely to involve the inhibition of proteins that repress *Mesp* transcription. Consistent with our hypothesis, siRNA-mediated knockdown of two transcription factors, *Tcf3* and *Foxa2,* is sufficient to up-regulate *Mesp1* expression and promote Kdr^+^ mesoderm formation. Mechanistically, Tcf3 is a well-characterized target of Id proteins (for review, see Yang et al. 2014) and is also known to interact with the Smad/Foxh1 complex to regulate Smad2/3-dependent transcription in mesendoderm progenitors (Yoon et al. 2011; Wills and Baker 2015). In turn, the potent endoderm determinant *Foxa2* (Stainier 2002; Viotti et al. 2014), is expressed in mesendoderm progenitors in a Smad2-dependent manner in mouse embryos (Vincent et al. 2003), suggesting that Tcf3 might cooperate with Smad2 to regulate Foxa2. Considering these functions, we suggest that the Id-driven blockade of Tcf3 and Foxa2 function drives two concomitant processes in mesendoderm progenitors: (1) the prevention of Acvr1b/Smad2-driven endoderm differentiation and (2) the activation of cardiac mesoderm specification via *Mesp* gene up-regulation.

### Id genes are essential for the specification of heart tube-forming progenitors

Heart formation in mammals involves a complex sequence of cell fate decisions and morphogenetic events that interdependently contribute to generate a four-chambered heart. Although we cannot completely rule out the possible contribution of endoderm morphogenetic defects to the absence of heart tube formation in *Id1–4* mutant embryos, several lines of evidence support that these defects directly result from impaired cardiac mesoderm specification due to the loss of Id protein activity in mesendoderm progenitors: (1) A closed foregut can be observed in the most anterior part of the embryo, where the heart tube normally forms (Supplemental Fig. S7D), suggesting that the endoderm could form and undergo morphogenesis. (2) Overall staining of cardiac mesoderm markers (*Smarcd3*, *Tbx5*, and *Nkx2.5*) is diminished in *Id1–4* mutant embryos as compared with controls (Fig. 6B–N), which suggests that fewer cardiac progenitors have formed. (3) siRNA-mediated loss of Id1 function impairs cardiac mesoderm formation in mESCs (Fig. 1J,K), a process that is de facto independent of endoderm morphogenesis in vitro. (4) Gain-of-function experiments show that Id proteins are sufficient to direct cardiac mesoderm specification in ESCs and *Xenopus* and expand the cardiac primordium in *Xenopus* embryos (Figs. 2, 3). Collectively, these observations suggest that the Id protein's requirement for anterior cardiac mesoderm specification is independent of endoderm differentiation and morphogenesis during early heart formation.

It is well described that most of the heart myocardium in mammals derives from two distinct populations of cardiac progenitors, referred to as heart fields (Kelly et al. 2001; Cai et al. 2003; Meilhac et al. 2004, 2015). However, it is not known whether similar or distinct molecular mechanisms regulate cardiac specification in these two cell populations. Our loss-of-function results showed that embryos lacking functional *Id1–4* genes fail to express cardiogenic mesoderm markers (*Smarcd3*, *Tbx5*, and *Nkx2.5*) in the most anterior region of the cardiac crescent at E7.75 and subsequently develop without forming a heart tube. In contrast, expression of these genes is maintained in the posterior region of the cardiac crescent within the splanchnic cardiac mesoderm (Supplemental Fig. S7A–E). Collectively, these observations suggest that only the most anterior subset of cardiac progenitors require Id1–4 activity for their specification. Consistent with our findings, Lescroart et al. (2014) have shown recently that early *Mesp1*-expressing mesoderm progenitors (around E6.5), which contribute to first heart field derivatives, express high levels of *Id1*. In contrast, late *Mesp1*-expressing cells (around E7.5), which contribute to second heart field derivatives, express low levels of *Id1* (see the Supplemental Material of Lescroart et al. 2014). Moreover, we show that human iMPs upregulate first heart field markers (*HCN4* and *TBX5*) during cardiac differentiation, while second heart field markers *(ISL1* and *SIX2*) are down-regulated (Supplemental Fig. S8A–F). Thus, we propose that *Id* genes normally specify first heart field progenitors that subsequently form the early heart tube. These findings also imply that cardiogenic mesoderm specification is not a singular process and can be initiated in an Id-dependent (first heart field progenitors) or Id-independent (posterior cardiac progenitors) manner during embryonic development.

### Id1-induced CMPs: a promising new technology for cardiac regenerative medicine and disease modeling

Recent studies have validated the concept of using ESC-derived cardiac progenitor transplantation to improve heart function post-injury in rodents, sheep, and nonhuman primate models (Menasche et al. 2015). Although promising, the use of these cells for therapeutic regeneration has not advanced substantially, largely because of the challenge of generating large numbers of defined cardiac progenitors from stem cells. This study demonstrates that simple overexpression of Id1 in hESCs (or human induced pluripotent stem cells [hiPSCs]) (data not shown) is sufficient to generate large amounts (>10^8^cells per batch) of cryopreservable and bona fide CMPs with remarkable abilities to spontaneously differentiate into beating cardiomyocytes (∼70% efficiency). These combined properties enable two major applications for Id1-programmed progenitors: (1) as a promising transplantable cell population to test for cardiac regenerative purposes after myocardial injury and (2) as a novel source of cells enabling large-scale production of hESC- or hiPSC-derived cardiomyocytes suitable for in vitro studies of cardiomyocyte physiology.

## Materials and methods

All animal handling and care followed the National Institutes of Health Guide for Care and Use of Laboratory Animals. The experimental protocols were approved by the Institutional Animal Care and Use Committees of the Sanford Burnham Prebys Medical Discovery Institute (SBP) (*Xenopus*) or the University of California at San Diego (mice).

### mESC culture

*Kdr*-eGFP mESCs (Ema et al. 2006) were maintained in DMEM high-glucose (HyClone) medium supplemented with 10% fetal bovine serum (FBS), 1 mM sodium pyruvate (Sigma), 1× MEM nNEAA (Gibco), 2 mM L-glutamine (Gibco), 100 U/mL penicillin–100 µg/mL streptomycin, (HyClone), 50 µM β-mercaptoethanol (Sigma), and 1000 U/mL LIF (Millipore). For differentiation, mESCs were seeded in 10-cm low-attachment tissue culture dishes at a density of 10^6^ cells per dish in a chemically defined medium (CDM) (Gadue et al. 2006), where they formed embryoid bodies (EBs) over a period of 2 d. At day 2, EBs were then dissociated using 0.25% Trypsin EDTA (GIBCO), washed in PBS, and replated in CDM supplemented with 50 ng/mL recombinant human Activin A (R&D Systems, 338-AC-050) in 10-cm low-attachment tissue culture dishes.

### *Kdr*-eGFP assay

On day 3 of differentiation, EBs were collected and dissociated using 0.25% Trypsin EDTA; 10^4^ cells per well were plated in 100 µL of CDM containing 50 ng/mL recombinant human Activin A in gelatin-coated 384-well optical tissue culture plates (Greiner Bio-One) and prespotted with 25 nM siRNAs in 0.2 µL of Lipofectamine RNAiMax + 14.8 µL of Opti-Mem I (Gibco). Fixation was performed at day 6 using 4% paraformaldehyde. Next, wells were imaged using an HT microscope (ImageXPress, Molecular Devices), and fluorescence was quantified using a custom method developed in MetaXpress analysis software (Molecular Devices) to determine the integrated pixel intensity of *Kdr*-eGFP.

### hESC culture

The H9 hESC line (WA09) was supplied by WiCell Research Institute. H9 cells were routinely maintained in mTeSR1 medium (Stem Cell Technologies, 05850) on growth factor-reduced Matrigel at 9 µg/cm^2^ and passaged every 4 d using ReLeSR (Stem Cell Technologies, 05872). hESCs were cultured for at least five passages before beginning differentiation. Cells were maintained with 2.5 mL of medium per 9.6 cm^2^ of surface area or equivalent. All pluripotent cultures were negative for mycoplasma contamination as routinely tested using a MycoAlert kit (Lonza).

### Lentivirus preparation

Large-scale lentivirus production was performed by the Viral Vector Core Facility at the SBP. Briefly, three plasmids, including lentivector, pCMVDR8.74, and pMD2.G, were cotransfected into HEK293T cells at a ratio of 3:2:1. UltraCulture serum-free medium (Lonza) supplemented with 1 mM L-glutamine (Life Technologies) was used to refeed transfected cells, and the supernatant was collected every 24 h from day 2 to day 4 after transfection. All viral supernatant was pooled and filtrated through 0.22-µm pores followed by concentration and purification using 20% sucrose gradient ultracentrifugation at 21,000 rpm for 2 h. The pellet containing concentrated viral particles was resuspended in PBS, aliquoted, and kept at −80°C for long-term storage.

### Generation of transgenic cell lines (mESCs and hESCs)

We applied the following modifications to pCDH-CMV vector (System Biosciences, CD511B-1): The CMV promoter driving the expression of the MCS was replaced by the *Ef1*α promoter to ensure robust expression in ESC stages, and the *Ef1*α*-*CopGFP cassette was replaced by a *pgk*-puro cassette to enrich for infected clones.mESCs with *Kdr*-eGFP (Ema et al. 2006) were infected with all possible combinations of high-titer lentiviruses (modified pCDH-CMV) (see above) overexpressing *Id1*, *Evx1*, or *Grrp1* and subsequently grown under continuous 2 µg/mL puromycin selection (Acros, 227420100).

Similarly, H9 hESCs were infected with *Id1*-overexpressing lentivirus and selected with 6 µg/mL puromycin.

### Generation of Id1-induced CMPs

#### Mouse Id1-induced CMPs

Id1-overexpressing mESCs were grown and differentiated as wild-type mESCs in the presence of 2 µg/mL puromycin. At day 3, cells were collected and dissociated with 0.25% Trypsin EDTA (Gibco), Trypsin was inactivated with 10% FBS-containing medium, and cells were washed in PBS and resuspended in CDM supplemented with 300 ng/mL recombinant human Activin A + 2 µg/mL puromycin. Cells (10^7^) were replated onto a 15-cm gelatin-coated tissue culture dish into 30 mL of CDM + 300 ng/mL recombinant human Activin A + 2 µg/mL puromycin and cultured for 3 d. At day 6, cells were collected and frozen in freezing medium (10% DMSO, 20% FBS, 70% DMEM high glucose [Hyclone]) at a density of 3 × 10^6^ to 5 × 10^6^ cells per vial and stored in liquid nitrogen.

#### Human Id1-induced CMPs

hESCs were dissociated using 0.5 mM EDTA (Life Technologies) in PBS without CaCl_2_ or MgCl_2_ (Corning, 21-040-CV) for 7 min at room temperature. Cells were plated at 3 × 10^5^ cells per well of a 12-well plate in mTeSR1 medium (Stem Cell Technologies) supplemented with 2 µM thiazovivin (Selleck Chemicals) for the first 24 h after passage. Cells were fed daily for 3–5 d until they reached ≥90% confluence, at which time they were washed with PBS, and the medium was changed to basal differentiation medium (BDM) consisting of RPMI 1640 medium (Life Technologies, 11875-093) and B27 supplement minus insulin (Life Technologies, A1895601). For the first 24-h differentiation period, the BDM was supplemented with 300 ng/mL recombinant human Activin A and 2 µg/mL puromycin (Acros, 227420100). After 24 h, this medium was replaced with basic BDM supplemented with 6 µg/mL puromycin. BDM + 6 µg/mL puromycin was replaced every 48 h. At day 5, cells were collected and frozen as described above.

### Differentiation of cryopreserved Id1-induced CMPs

To resume differentiation, CMPs (mouse or human) were thawed for 3 min in a 37°C water bath, washed, resuspended in 2 µM BDM^+^ hESC recovery supplement (Stemgent) for human Id1-induced CMPs or 2 µM CDM^+^ hESC recovery supplement for mouse Id1-induced MMPs, and plated onto Matrigel-coated 384-well culture plates (Greiner Bio-One) at a cell density of 25,000 cells per well. Medium (BDM or CDM) was replaced every other day until day 15 of differentiation.

### siRNAs

siRNAs from Figures 1F and 3F were cherry-picked at the International Centre for Genetic Engineering and Biotechnology (Trieste, Italy) from the mouse genome-wide siGENOME SMARTpool library from Dharmacon and transfected at a final concentration of 12.5 nM. All remaining siRNAs were purchased from Life Technologies (silencer select siRNAs) and transfected at a final concentration of 25 nM: siControl (AM4611), siEvx1 (s65742), siFoxa2 (s67627), siGrrp1 (s91214), siId1 (s68006), siTcf3 (s74856), siTcf4 (s74829), and siTcf12 (s74811).

### Immunostaining for cell culture and cardiovascular lineage quantification

Cells grown on gelatin-coated 384-well plates (Greiner Bio-One) were fixed using 4% paraformaldehyde and immunostained by incubating in block solution (10% horse serum, 0.5% Triton X-100, 0.01% gelatin in phosphate-buffered saline [PBS] [Cellgro]) for 30 min at room temperature followed by incubation with antibodies directed against Foxa2 (Santa Cruz Biotechnology, sc-6554), Pecam1 (Santa Cruz Biotechnology, sc-1506), Actc1 (Sigma, A7811), and CDH5 (R&D Systems, AF938) for 1 h at room temperature in the block solution. The cells were then washed three times with PBS and incubated with Alexa-conjugated secondary antibodies (Life Technologies) in the block solution for 1 h at room temperature. The cells were then washed three times with PBS and stored in 50% (v/v) glycerol in PBS. Next, wells were imaged using a HT microscope (ImageXPress, Molecular Devices), and fluorescence was quantified using a custom method developed in MetaXpress Analysis software (Molecular Devices) to determine the percentages of ACTC1-, TAGLN-, and CDH5-positive cells.

### RT-qPCR

Total RNA was extracted with a miRVana isolation kit (Ambion, AM1540) and reverse-transcribed to cDNA with a QuantiTect reverse transcription kit (Qiagen) according to the manufacturer's instructions. cDNA samples synthesized from 1 µg of total RNA were subjected to RT-qPCR with the 7900HT Fast real-time PCR system (Applied Biosystems) using the iTaq SYBR Green Supermix with ROX (Bio-Rad). Primer sequences are listed in Supplemental Table S2. The data were analyzed with the ΔΔCt method, applying β-Actin as a normalization control.

### Microarray experiment and analysis

siControl or siAcvr1b was transfected at day 3 in differentiating mESCs. Total RNA (500 ng) was collected at day 4 and hybridized on MouseRef-8 v2.0 Expression BeadChip (25,600 transcripts; Illumina). BeadChips were subsequently washed and developed with Fluorolink streptavidin-Cy3 (GE Healthcare). BeadChips were scanned with an Illumina BeadArray Reader, and hybridization efficiency was monitored using BeadStudio software (Illumina) to measure internal controls built into the Illumina system. Linear models were fitted for each gene using the Bioconductor limma package in R. Moderated *t* statistics and fold change and the associated *P*-values were calculated for each gene. To account for testing thousands of genes, we reported false discovery rate (FDR)-adjusted values, which were calculated using the Benjamini-Hochberg method.

### Flow cytometry

For live *Kdr*-eGFP cells, cells were dissociated using 0.25% Trypsin EDTA, blocked with 10% FBS-containing medium, and resuspended in PBS containing 0.5% FBS (washing buffer) for flow sorting using LSRFortessa or FACSAria flow cytometers (BD Biosciences). For hESCs, day 5 cells were dissociated using 1× TrypLE Express (Gibco), blocked, and washed with PBS containing 0.5% FBS (washing buffer). Cells were incubated for 20 min with PE anti-human CD309 (1:100 dilution; BioLegend, 359903) in PBS containing 0.5% FBS at 4°C. Next, cells were washed in washing buffer, fixed for 20 min in 1% PBS:formaldehyde at 4°C, washed in PBS, and resuspended in washing buffer and processed by flow sorting.

### *Xenopus laevis* embryo culture

Embryos were fertilized in vitro, dejellied in 2% cysteine–HCl (pH 7.8), and maintained in 0.1× MMR (Peng 1991). Embryos were staged according to Nieuwkoop and Faber (Nieuwkoop 1967). For gene expression analysis, whole embryos were fixed in MEMFA for in situ hybridization as below.

### mRNA injection in *X. laevis*

Synthetic capped mRNAs for *Xid2* injection were transcribed from pSP64T plasmid using SP6 mMessage kit (Ambion). mRNAs were injected at 125 ng per blastomere in four-cell stage embryos.

### In situ hybridization in *X. laevis* embryos

In situ hybridization for *Xbra* (Colas et al. 2008) and *Xmespb* was carried out as described previously (Djiane et al. 2000).

### Mouse embryos

Mouse embryos were dissected into DEPC-treated PBS, fixed overnight in 4% PFA, and dehydrated into MeOH. In situ hybridization used *Id1*, *Grrp1*, *Evx1* (cloned into pGEM), and *Mesp1* (Saga et al. 1996) probes (60°C hybridization) as described in Wilkinson and Nieto (1993). For histology, embryos were embedded in paraffin, H&E stained, and sectioned (5-µm thickness) following standard procedures. Sections were scanned at high magnification (40×) using a Leica Aperio AT2.

### CRISPR/Cas9 Id gene editing of mouse embryos

In order to generate Id1–4 mutant embryos, eight sgRNAs were designed to target sites near the ATG translation initiation site and near the beginning of the HLH domain for each Id gene (see the Supplemental Material) using the tool at http://crispr.mit.edu to ensure maximum specificity. DNA templates for sgRNAs were generated by PCR amplification (Phusion DNA polymerase, New England Biolabs) of ssDNA ultramer oligonucleotides (Integrated DNA Technologies) (see the Supplemental Material); sgRNAs were transcribed from these templates using HiScribe T7 high-yield RNA synthesis kit (New England Biolabs) and purified using Megaclear kit (Life Technologies). For mouse zygote injections, 50 ng/µL Cas9 mRNA (Life Technologies) and 20 ng/µL each sgRNA were combined in nuclease-free water. Fertilized oocytes were collected from 3- to 4-wk-old superovulated C57Bl6 females (prepared by injecting 5 IU each of pregnant mare serum gonadotropin and human chorionic gonadotropin [Sigma Aldrich]), transferred into M2 medium (Millipore), and injected with the Cas9 mRNA/sgRNA solution into the cytoplasm. Injected embryos were then reimplanted into recipient pseudopregnant ICR female mice. Implanted females were sacrificed 8–9 d after reimplantation; yolk sac DNA was collected for genotyping by PCR (Bioline MyTaq extract kit) (see the Supplemental Material for primer sequences) followed by DNA deep sequencing (Ilumina Nextera kit for library preparation and Illumina HiSeq 1500 for sequencing). Sequences were analyzed, and variant alleles were recorded using Integrative Genomics Viewer (IGV) genome browser (Broad Institute). For off-target analysis, the top eight off-target sites were identified using the tool at http://crispr.mit.edu; these regions were PCR-amplified (see the Supplemental Material for off-target sites and primer sequences) and Sanger-sequenced.

### Statistics

Each experiment represents at least quadruplicate biological replicates per condition. Statistical analysis was performed with unpaired Student's *t*-test (*P* < 0.05).

## Supplementary Material

Supplemental Material
