# Supplementary material for: Id genes are essential for early heart formation
Source: Genes Dev. 2017 Jul 1;31(13):1325–38. doi: 10.1101/gad.300400.117 (PMC5580654; doi:10.1101/gad.300400.117)
Supplement: Supplemental Material [file supp_31_13_1325__index.html]

Id genes are essential for early heart formation — Supplemental Material 

# Id genes are essential for early heart formation

## Supplemental Material

undefined

- Supplemental\_Movie\_S6.avi
- Supplemental\_Files.pdf
- Supplemental\_Movie\_S3.avi
- Supplemental\_Movie\_S1.wmv
- Supplemental\_Movie\_S4.avi
- Supplemental\_Movie\_S2.wmv
- Supplemental\_Movie\_S5.avi
