# Supplementary material for: Id genes are essential for early heart formation
Source: Genes Dev. 2017 Jul 1;31(13):1325–38. doi: 10.1101/gad.300400.117 (PMC5580654; doi:10.1101/gad.300400.117)
Supplement: Supplemental Material [file supp_gad.300400.117_Supplemental_Files.docx]

**Supplemental Figures**


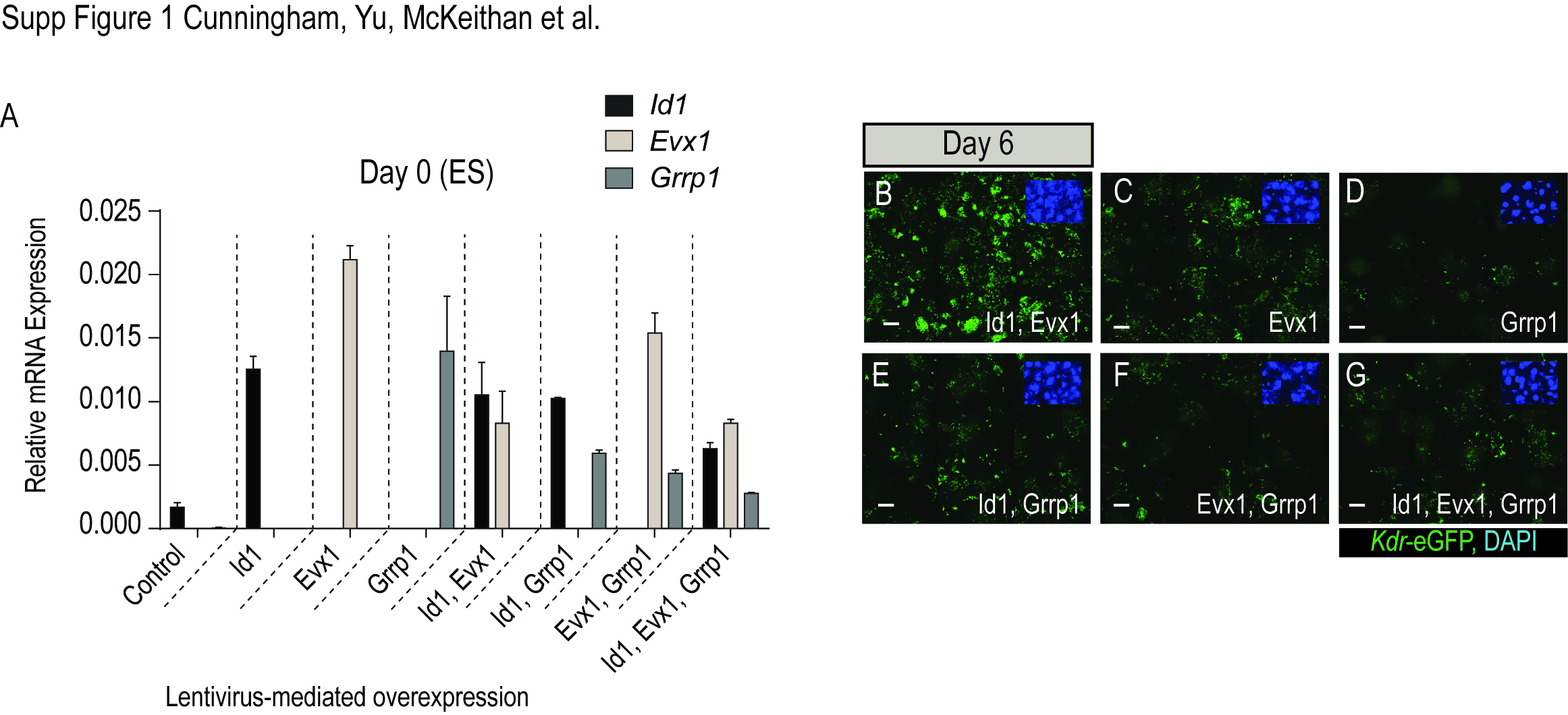


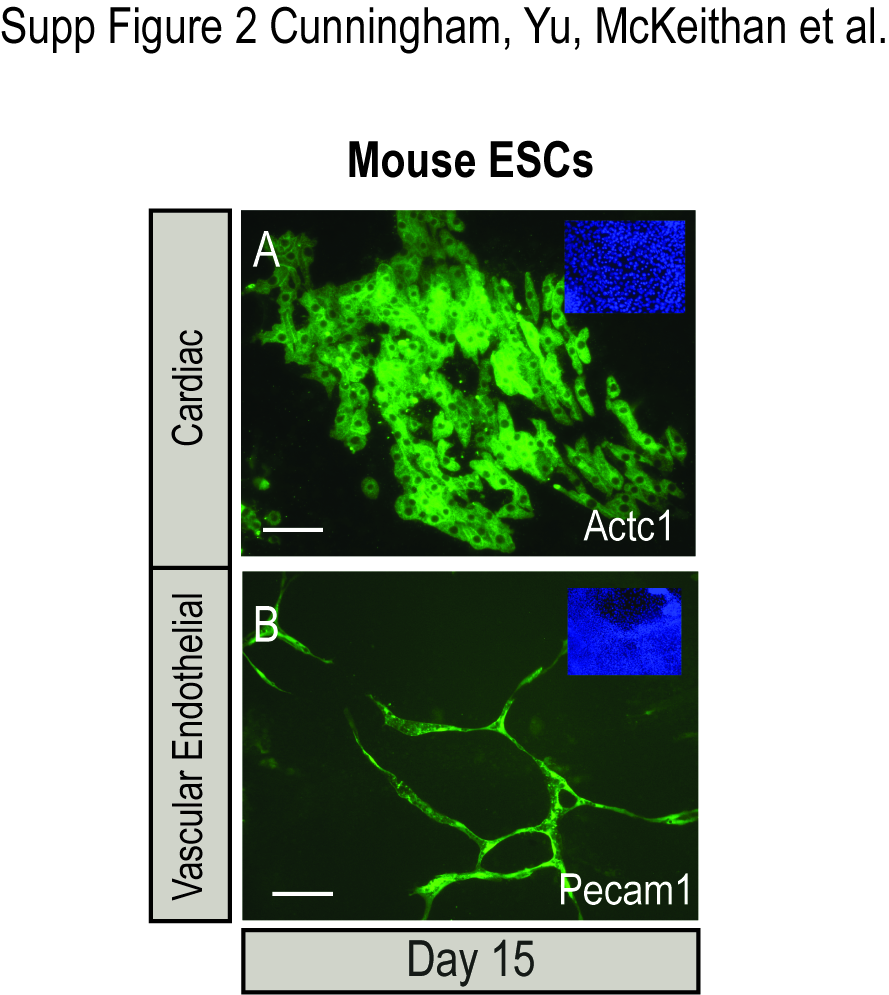


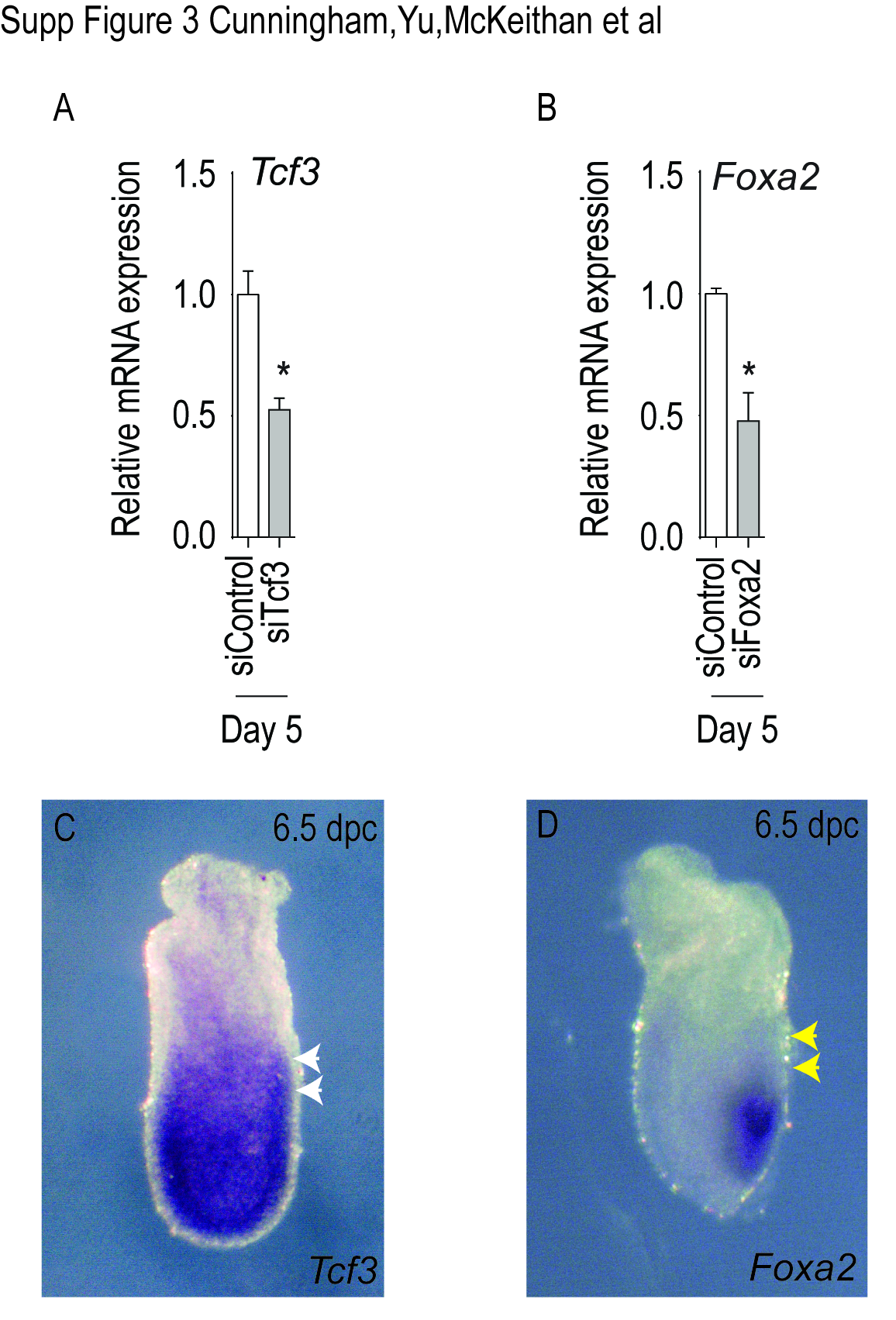


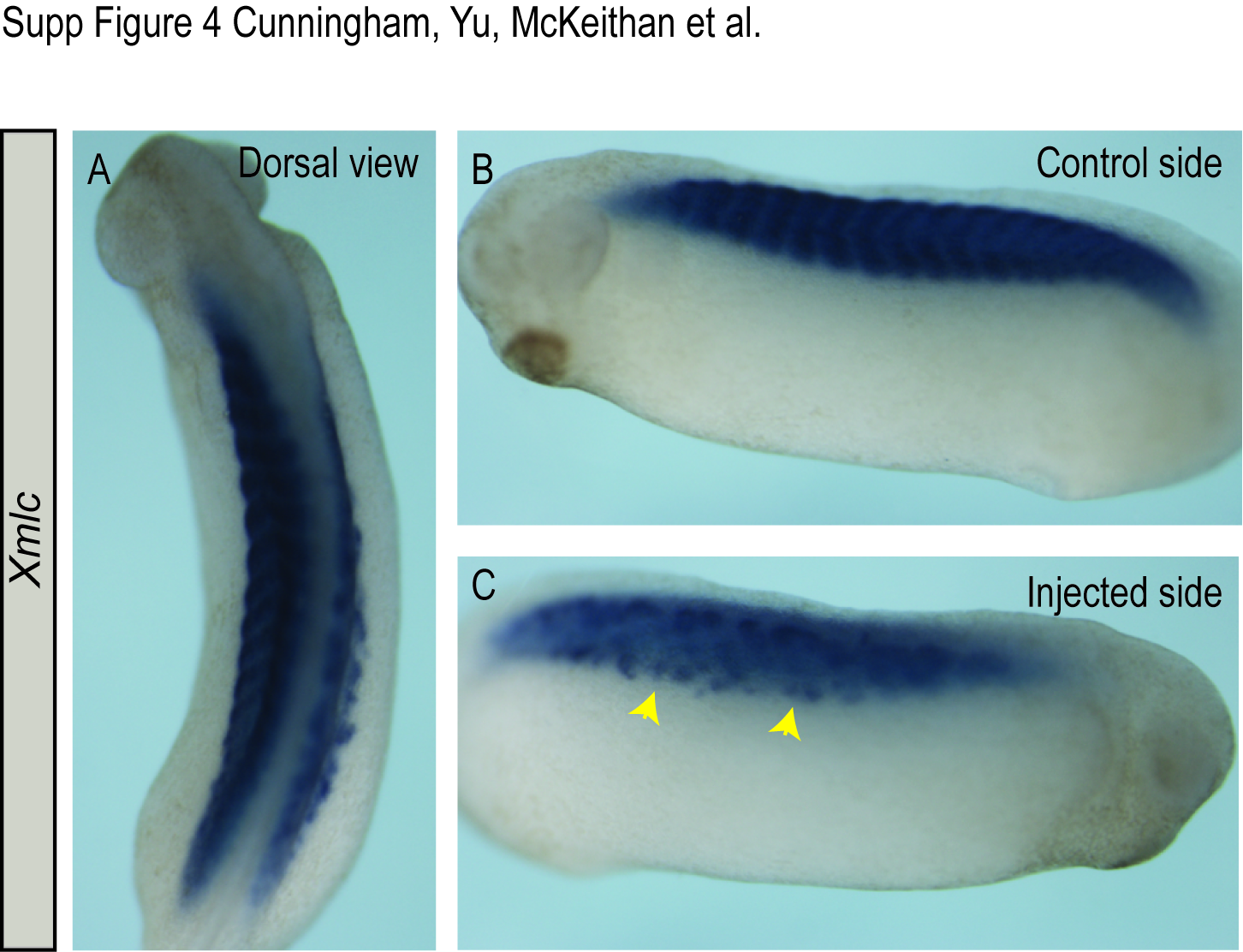


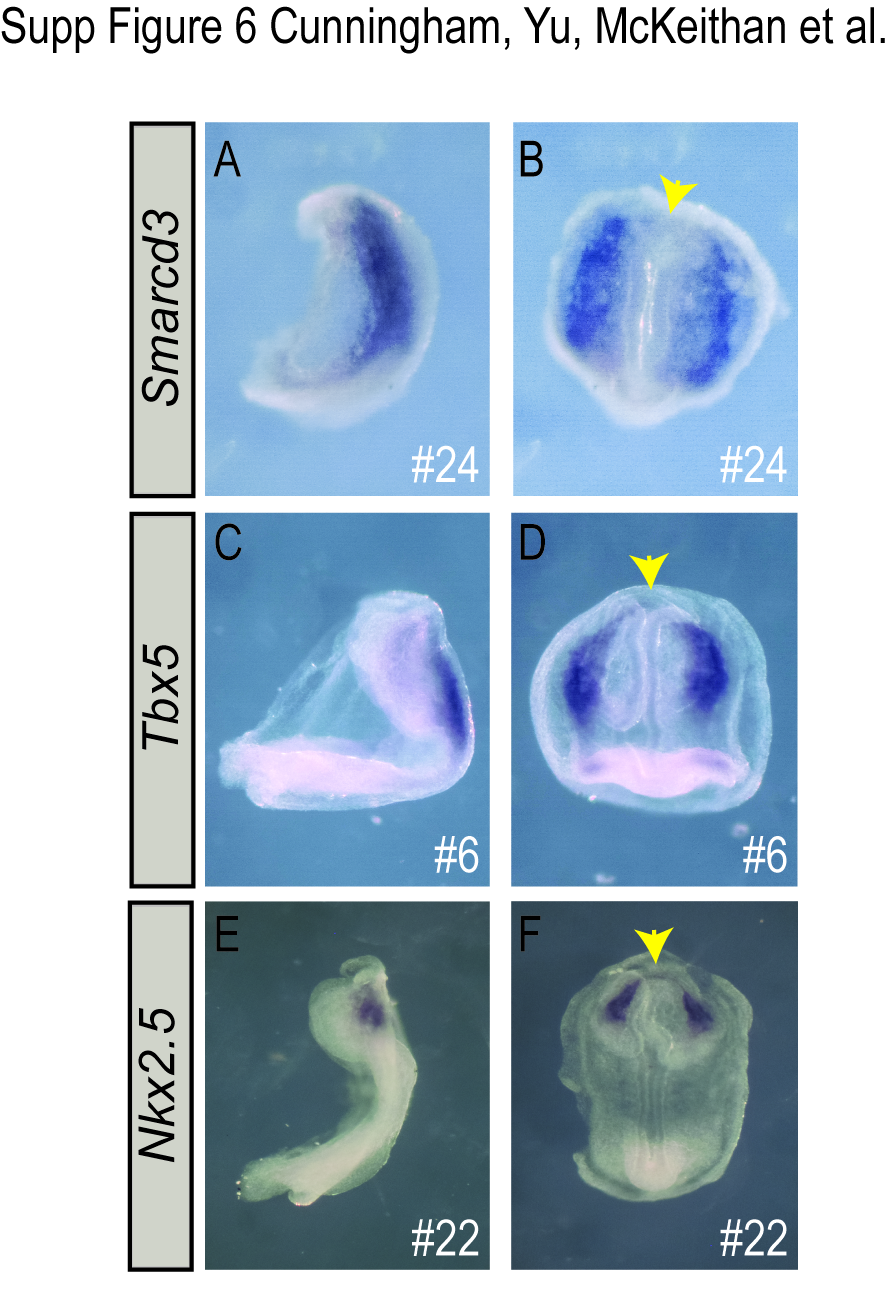


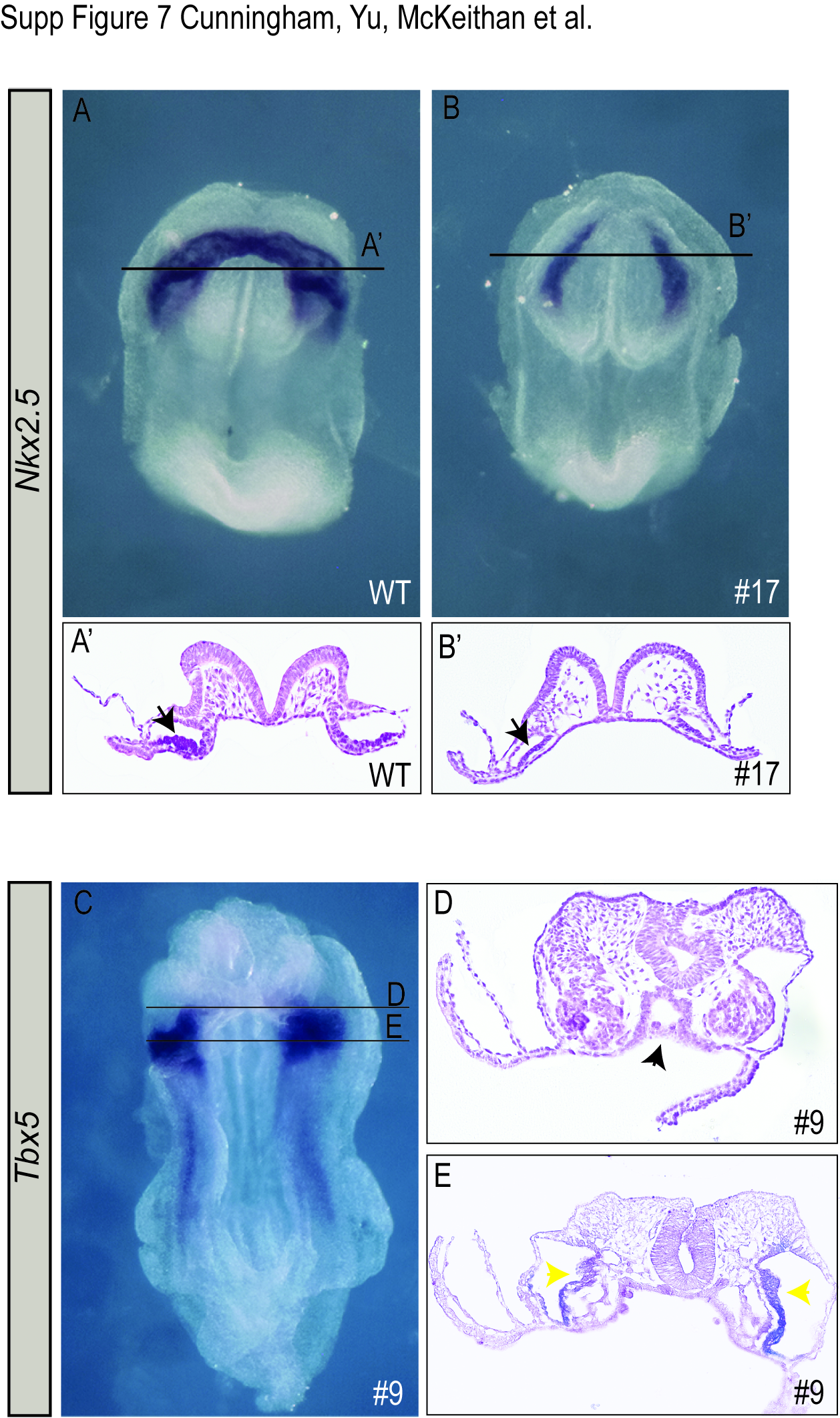


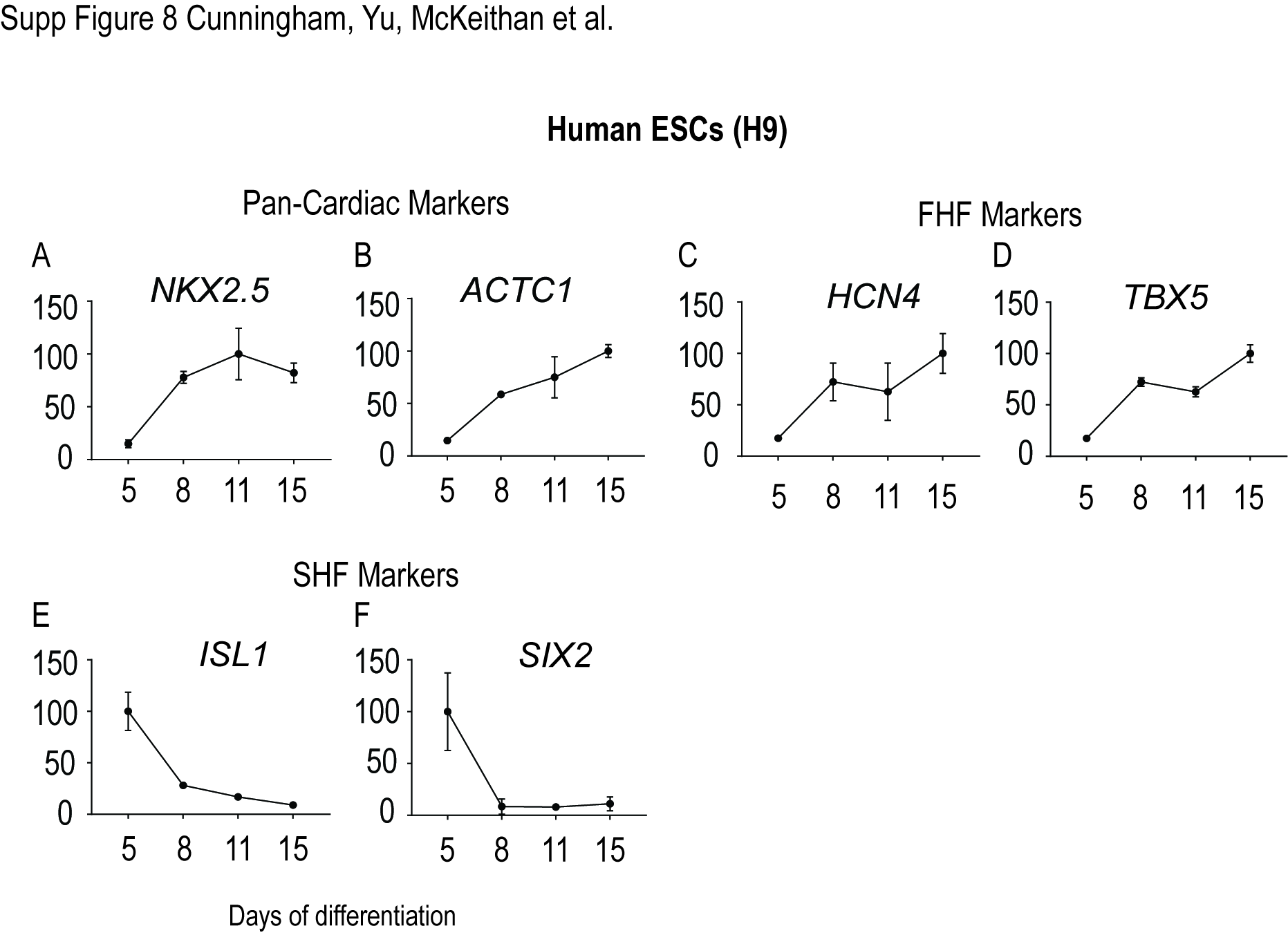


# Supplemental Tables

# Supplemental Table 1: Gene Candidates Regulated by siAcvr1b

|  | **Fold Upregulation** | **Gene ID** |  | **Fold Downregulation** | **Gene ID** |
| --- | --- | --- | --- | --- | --- |
|  | **P-Value <0.05** | |  | **P-Value <0.05** |  |
| 1 | 2.609411 | Id1 | 1 | 1.6015979 | Zmpste24 |
| 2 | 2.0134268 | Gadd45g | 2 | 1.6013767 | Cdc42 |
| 3 | 1.5968683 | Irx3 | 3 | 1.5671402 | Egr1 |
| 4 | 1.4899422 | Sox9 | 4 | 1.5383662 | Fgf8 |
| 5 | 1.4583107 | Evx1 | 5 | 1.525815 | Leftb |
| 6 | 1.4510411 | Cxcl12 | 6 | 1.515485 | Acvr1b |
| 7 | 1.4346725 | Rgma | 7 | 1.4910644 | Zic2 |
| 8 | 1.4037634 | Tnrc6a | 8 | 1.4896251 | Ppp4r4 |
| 9 | 1.3917822 | Gadd45g | 9 | 1.467095 | Gemin6 |
| 10 | 1.3839511 | Gbx2 | 10 | 1.4522598 | Trim67 |
| 11 | 1.3793361 | BC030476 | 11 | 1.4373533 | Notch3 |
| 12 | 1.3663218 | Fus | 12 | 1.4351145 | Srprb |
| 13 | 1.365738 | Irx5 | 13 | 1.4311734 | Gsc |
| 14 | 1.3544436 | Elavl3 | 14 | 1.4305644 | Zmpste24 |
| 15 | 1.3542395 | Crabp2 | 15 | 1.4290521 | Igfbp3 |
| 16 | 1.3431355 | 1500011K16Rik | 16 | 1.4245033 | Zmpste24 |
| 17 | 1.3422825 | Tnrc6 | 17 | 1.4184229 | Grm6 |
| 18 | 1.3389522 | Ccdc85b | 18 | 1.4032689 | Ube2q |
| 19 | 1.329755 | Grrp1 | 19 | 1.3981106 | Lbr |
| 20 | 1.3260252 | Fgfbp3 | 20 | 1.3852041 | Tmem63a |
| 21 | 1.3247453 | Bcl2l11 | 21 | 1.3845202 | Pitx2 |
| 22 | 1.3216742 | Slc1a3 | 22 | 1.382626 | Npm3 |
| 23 | 1.3198931 | Gtl2 | 23 | 1.3775356 | Ttc19 |
| 24 | 1.3195066 | Id3 | 24 | 1.3773962 | Stk4 |
| 25 | 1.3176388 | Bckdha | 25 | 1.3745593 | Sgk |
| 26 | 1.3164426 | Chka | 26 | 1.3733177 | Armcx2 |
| 27 | 1.3119096 | Chd4 | 27 | 1.365576 | Tmem63a |
| 28 | 1.3110358 | Rras | 28 | 1.3652078 | Wdr82 |
| 29 | 1.3094118 | Mrg1 | 29 | 1.3617791 | Gemin6 |
| 30 | 1.3044847 | Lsm12 | 30 | 1.3600438 | Ppp4r4 |
| 31 | 1.3036366 | Cbln1 | 31 | 1.359722 | Tcn2 |
| 32 | 1.3035693 | Zfp296 | 32 | 1.3578465 | Dnajc6 |
| 33 | 1.3024908 | Klf7 | 33 | 1.3522204 | Ncoa4 |
|  |  |  | 34 | 1.3519416 | Prpf8 |
|  |  |  | 35 | 1.3507187 | Hdlbp |
|  |  |  | 36 | 1.346875 | Rnf213 |
|  |  |  | 37 | 1.3434738 | Nodal |
|  |  |  | 38 | 1.3346514 | Slc19a2 |
|  |  |  | 39 | 1.3340039 | Rab1 |
|  |  |  | 40 | 1.332933 | Klhl22 |
|  |  |  | 41 | 1.3268061 | Foxa2 |
|  |  |  | 42 | 1.3243924 | Zfp750 |
|  |  |  | 43 | 1.323197 | Map2k4 |
|  |  |  | 44 | 1.3197291 | Eppk1 |
|  |  |  | 45 | 1.3165845 | Car2 |
|  |  |  | 46 | 1.3164321 | Smarca5-ps |
|  |  |  | 47 | 1.3136374 | Lefty1 |
|  |  |  | 48 | 1.3131495 | Cnn3 |
|  |  |  | 49 | 1.3128709 | Igfbp3 |
|  |  |  | 50 | 1.311379 | Ints5 |
|  |  |  | 51 | 1.30427 | Tgfbr3 |
|  |  |  | 52 | 1.3032453 | Chst15 |
|  |  |  | 53 | 1.3031058 | Atl2 |

# Supplemental Table 2: Quantitative PCR oligonucleotide primers

**Mouse primers**

| Gene Name | **Gene Bank Accession** | **Sequence** |
| --- | --- | --- |
| Acrv1b | NM_007395 | F: TTCTTCCCCCTTGTTGTCCTC R: ACAGGTGTAGTTGGTCTGTAGG |
| Actc1 | NM_009608 | F: CTGGATTCTGGCGATGGTGTA R: CGGACAATTTCACGTTCAGCA |
| Actn3 | NM_013456 | F: AACAGCAGCGGAAAACCTTCA R: GGCTTTATTGACATTGGCGATTT |
| Cdh11 | NM_009866 | F: CTGGGTCTGGAACCAATTCTTT R: GCCTGAGCCATCAGTGTGTA |
| Cdh5 | NM_009868 | F: CACTGCTTTGGGAGCCTTC R: GGGGCAGCGATTCATTTTTCT |
| Chst15 | NM_029935 | F: TTCCCCGAAGACACACACAAA R: CCCCAGTTTTCATTGCCCTCA |
| Crabp2 | NM_007759 | F: ATGCCTAACTTTTCTGGCAACT R: GCACAGTGGTGGAGGTTTTGA |
| Cxcl12 | NM_001012477 | F: TGCATCAGTGACGGTAAACCA R: TTCTTCAGCCGTGCAACAATC |
| Dnajc6 | NM_001164584 | F: TGAAAATAAAGGTGCCTCGTCTC R: TCAGGTTACTGAATAGCCTCCC |
| Egr1 | NM_007913 | F: TCGGCTCCTTTCCTCACTCA R: CTCATAGGGTTGTTCGCTCGG |
| Elavl3 | NM_010487 | F: TCCTATGCACGTCCCAGTTCT R: TCGATCCTCTTGTCAAAGCGG |
| Evx1 | NM_007966 | F: GAGAGCCGAAAGGACATGGTT R: CTGCCTGCTAGTCCATCGAC |
| Fgf8 | NM_001166361 | F: CCGAGGAGGGATCTAAGGAAC R: CTTCCAAAAGTATCGGTCTCCAC |
| Fgfbp3 | NM_028263 | F: GGTCGCTTCGTGAGTCCAG R: AGCAGCCGTCTCCAGTAGT |
| FoxA2 | NM_010446 | F: CCCTACGCCAACATGAACTCG R: GTTCTGCCGGTAGAAAGGGA |
| Gadd45g | NM_011817 | F: GGGAAAGCACTGCACGAACT R: AGCACGCAAAAGGTCACATTG |
| Gbx2 | NM_010262 | F: CAACTTCGACAAAGCCGAGG R: ACTCGTCTTTCCCTTGCCCT |
| Gemin6 | NM_026053 | F: GCCAACATTGTCCTCGTAAACT R: TGTGGTCCCCTTCACTTATGG |
| Grm6 | NM_173372 | F: GCAGAAACATCTGGTTTGCTG R: CCTCCTGTTCATAGGTGGAGTC |
| Grrp1 | NM_001099296 | F: AGGGACCACTGCAACTCAG R: CCATACACAGTTAAGGACGCAC |
| Gsc | NM_010351 | F: CAGATGCTGCCCTACATGAAC R: TCTGGGTACTTCGTCTCCTGG |
| Id1 | NM_010495 | F: CCTAGCTGTTCGCTGAAGGC R: CTCCGACAGACCAAGTACCAC |
| Id3 | NM_008321 | F: CGACCGAGGAGCCTCTTAG R: GGACGCGATAGGGAAGACC |
| Irx3 | NM_001253822 | F: TCTGGGTCCCTATCCAATGTG R: GGTCCCCGAACTGGTACTG |
| Irx5 | NM_018826 | F: TACAGCACCAGCGTCATTTCG R: GAGCCCACGTAAGAGAAGGC |
| Kdr | NM_010612 | F: TTTGGCAAATACAACCCTTCAGA R: GCAGAAGATACTGTCACCACC |
| Lefty1 | NM_010094 | F: CCAACCGCACTGCCCTTAT R: CGCGAAACGAACCAACTTGT |
| Lefty2 | NM_177099 | F: CAGCCAGAATTTTCGAGAGGT R: CAGTGCGATTGGAGCCATC |
| Mesp1 | NM_008588 | F: GTCACTCGGTCCTGGTTTAAG R: ACGATGGGTCCCACGATTCT |
| Myh6 | NM_010856 | F: GCCCAGTACCTCCGAAAGTC R: GCCTTAACATACTCCTCCTTGTC |
| Myog | NM_031189 | F: GAGACATCCCCCTATTTCTACCA R: GCTCAGTCCGCTCATAGCC |
| Nodal | NM_013611 | F: TTCAAGCCTGTTGGGCTCTAC R: TCCGGTCACGTCCACATCTT |
| Pecam1 | NM_001032378 | F: ACGCTGGTGCTCTATGCAAG R: TCAGTTGCTGCCCATTCATCA |
| Pitx2 | NM_011098 | F: GCAGCCGTTGAATGTCTCTTC R: GTCCGTGAACTCGACCTTTTT |
| Snai1 |  | F: CACACGCTGCCTTGTGTCT R: GGTCAGCAAAAGCACGGTT |
| Sox9 | NM_011448 | F: GAGCCGGATCTGAAGAGGGA R: GCTTGACGTGTGGCTTGTTC |
| Stk4 | NM_021420 | F: TCATTCGGCTACGGAACAAGA R: GACCTGCGACTCCAAAGTCTG |
| Tnnt2 | NM_001130181 | F: CAGAGGAGGCCAACGTAGAAG R: CTCCATCGGGGATCTTGGGT |
| Tnrc6a | NM_144925 | F: ATGCTCCTGAAAGCAAACCAG R: CCTTTTAGGGCAAGTCCATTGT |
| Trim67 | NM_198632 | F: CCACTCTCTGCGAGCAATG R: GGTGGCTGAACTAGCCGAT |
| Zfp750 | NM_178763 | F: ATGAGTCTCCTAAAGGAACGGA R: GGGAATACGATCTTGCTCTGAC |
| Zmpste24 | NM_172700 | F: GCATCGGTGGACGCTATGT R: TGTGCTAGGAAGGTCTCCCAA |

**Human primers**

| Gene Name | **Gene Bank Accession** | **Sequence** |
| --- | --- | --- |
| ACTA2 | NM_001613 | F: CTATGAGGGCTATGCCTTGCC R: GCTCAGCAGTAGTAACGAAGGA |
| ACTN3 | NM_001104 | F: GATGACCCCATCGGAAACCTG R: CTTGCAGATCCTGTTGGCAG |
| CDH11 | NM_001797 | F: GTATCCTCGAAGGACAACCCT R: GACATCGGTCAGTGTGATCGT |
| CDH5 | NM_001795 | F: AAGCGTGAGTCGCAAGAATG R: TCTCCAGGTTTTCGCCAGTG |
| EVX1 | NM_001989 | F: GACCAGATGCGTCGTTACCG R: GTGGTTTCCGGCAGGTTTAG |
| GRRP1 | NM_024869 | F: TCAAGACGCACCAGGTGATAG R: CGGTAGAAGATGAGGGAATCAGG |
| HCN4 | NM_005477 | F: TGGACACCGCTATCAAAGTGG R: CTGCCGAACATCCTTAGGGA |
| ID1 | NM_181353 | F: CTGCTCTACGACATGAACGG R: GAAGGTCCCTGATGTAGTCGAT |
| ISL1 | NM_002202 | F: GCGGAGTGTAATCAGTATTTGGA R: GCATTTGATCCCGTACAACCT |
| KDR | NM_002253 | F: GTGATCGGAAATGACACTGGAG R: CATGTTGGTCACTAACAGAAGCA |
| MEF2C | NM_001193349 | F: CCAACTTCGAGATGCCAGTCT R: GTCGATGTGTTACACCAGGAG |
| MESP1 |  | F: CCACCGTCCCCGCTCCTTCC R: CGGTGCTCACAGAGACGGCG |
| MYH6 | NM_002471 | F: GCTGGTCACCAACAATCCCTA R: CGTCAAAGGCACTATCGGTGG |
| MYH11 | NM_022844 | F: CATCTACTCGGAGAAGATCGTCG R: CGCCTGTGCATAGAATGGACT |
| MYOG | NM_002479 | F: GGGGAAAACTACCTGCCTGTC R: AGGCGCTCGATGTACTGGAT |
| PECAM1 | NM_000442 | F: CCAAGGTGGGATCGTGAGG R: TCGGAAGGATAAAACGCGGTC |
| SIX2 | NM_016932 | F: AAGGCACACTACATCGAGGC R: CACGCTGCGACTCTTTTCC |
| SNAI1 | NM_005985 | F: TCGGAAGCCTAACTACAGCGA R: AGATGAGCATTGGCAGCGAG |
| TBX5 | NM_000192 | F: CTGTGGCTAAAATTCCACGAAGT R: GTGATCGTCGGCAGGTACAAT |
| TNNT2 | NM_001001431 | F: ACAGAGCGGAAAAGTGGGAAG  R: TCGTTGATCCTGTTTCGGAGA |

# Supplemental Information

## Supplemental Figure 1: Generation of mESC lines stably overexpressing all possible combinations of Id1, Evx1 and Grrp1

A) RT-qPCR analysis shows the establishment of mESC cell-line overexpressing all possible combinations of Id1, Evx1 and Grrp1 as compared to control mESCs.

B-G) Representative images of *Kdr*-eGFP fluorescence at day 6 of differentiation in mESCs overexpressing all possible combinations of the three candidate genes. Scale bar is 50 µm

Quantitative data are presented as means +/- SD. All experiments were performed at least in biological quadruplicates.

## Supplemental Figure 2: Differentiation potential of Id-1 induced mouse cardiogenic mesoderm progenitors

A-B) Representative immunofluorescence images for cardiomyocytes (*Actc1*) and vascular endothelial cells (*Pecam1*) at day 15 of differentiation. Scale bar is 50 µm

## Supplemental Figure 3: Knock-down efficiency for Tcf3 and Foxa2 and endogenous expression of *Tcf3 and Foxa2* in E6.5 embryos

A-B) qRT-PCR analysis showing siTcf3 (A) and siFoxa2 (B)-mediated knock-down efficiency as compared to siControl at day 5 of differentiation. Quantitative data are presented as means +/- SD. All experiments were performed at least in biological quadruplicates.

C-D) Endogenous expression of *Tcf3 and Foxa2* in E6.5 mouse embryos by *in situ* hybridization. *Tcf3* expression is ubiquitous throughout the embryo (C) and overlaps with Id1 expression in the most proximal region of the embryo (white arrows). In contrast at E6.5**,** *Foxa2* transcripts are expressed in the anterior primitive streak (D), however in a domain that is more distal and non-overlapping with *Id1* (yellow arrows).

## Supplemental Figure 4: Skeletal muscle differentiation is impaired in response to Xid2 overexpression in *Xenopus laevis*

A-C) Embryos were hemilaterally injected at 4-cell stage as in Figure 5 and cultured to early tailbud stage (stage 25). Whole mount *in situ* hybridization probes for skeletal muscle marker *Xmlc* expression. Yellow arrows indicate decreased and disorganized pattern of expression in the injected side as compared to control side.

## Supplemental Figure 5: Summary of genotype information of mouse embryos analyzed in loss of function study

DNA sequences of 24 mutant embryos were analyzed, and variant alleles were recorded using IGV genome browser (Broad Institute). Blue box marks wild type allele, red box marks null alleles, orange box marks large (>50bp) in-frame deletion, yellow box marks small (<25bp) in-frame deletion. Phenotypes are annotated as follows: N: no cardiac defect, S: severe loss (or absence) of medial staining in cardiac crescent, A: absence of heart tube, CB: cardiac bifida, LF: looping failure. Two embryo stages are reported: CC: cardiac crescent stage and HT: heart tube stage. Four cardiac markers were tested: *Smarcd3*, T*bx5,* *Nkx2.5,* and *Fgf8*.

## Supplemental Figure 6: Id genes are essential for early heart formation

## A-F) Additional *in situ* hybridization results from Id1-4 mutants (individual mutants are marked by a #). *Smarcd3* at E7.75 (A-B), *Tbx5* at E8.0 (C-D), *Nkx2.5* at E8.25 (E-F). Yellow arrowheads point to missing heart tube forming region at cardiac crescent stages or the missing heart tube. A,C,E) Lateral view B,D,F) Ventral View

## Supplemental Figure 7: Cardiac mesoderm is present posterior to heart tube location in Id1-4 mutant embryos

A-B) Ventral view of WT and embryo #17 after *Nkx2.5* *in situ* hybridization. A’-B’) Transverse section posterior to heart tube location showing expression of *Nkx2.5* expression in cardiac splanchnic mesoderm marked by yellow arrows.

C) Ventral view of embryo #9 after *Tbx5* *in situ* hybridization. D) Transverse section at heart tube level confirms the absence of anatomical heart tube and mesoderm between neural tube and foregut. Black arrow shows the presence of a closed foregut in the region of the embryo were the primitive heart tube normally forms E) More posterior transverse section showing expression of *Tbx5* expression in cardiac splanchnic mesoderm marked by yellow arrows.

## Supplemental Figure 8: Human iMPs express first heart field markers during cardiac differentiation

A-F) Temporal expression profiles of pan-cardiac markers *NKX2.5* (A), *ACTC1* (B); first heart field markers *HCN4* (C), *TBX5* (D); and second heart field markers *ISL1*(E), *SIX2* (F) during cardiac differentiation of h9-hESCs stably overexpressing *Id1* from day 5 to day 15. Quantitative data are presented as means +/- SD. Experiments were performed in biological quadruplicates.

**Supplemental Methods**

**sgRNA target sequences**

**ATG translational start site**

**TGA** or **TAA translational stop site**

**NNNNNNNNNNNNNNNNNNNNNNN sgRNA target site + PAM**

**NNNNNNNNNN HLH domain**

nnnnnnnnnn intronic sequence

**Mouse Id1 (Chromosome 2)**

CTCTGTTCTCAGCCTCCTCCGCTCCCCTCC**GCCTGTTCTCAGGATCATGAAGG**TCGCCAGTGGCAGTGCCGCAGCCGCTGCAGGCCCTAGCTGTTCGCTGAAGGCGGGCAGGACAGCGGGCGAGGTGGTACTTGGTCTGTCGGAGCAAAGCGTGGCCATCTCGCGCTGCGCTGGGACGCGCCTGCCCGCCTTGCTGGACGAGCAGCAGGTGAAC**GTCCTGCTCTACGACATGAACGG**CTGCTACTCACGCCTCAAGGAGCTGGTGCCCACCCTGCCCCAGAACCGCAAAGTGAGCAAGGTGGAGATCCTGCAGCATGTAATCGACTACATCAGGGACCTGCAGCTGGAGCTGAACTCGGAGTCTGAAGTCGGGACCACCGGAGGCCGGGGACTGCCTGTCCGCGCCCCGCTCAGCACCCTGAACGGCGAGATCAGTGCCTTGGCGGCCGAGgtgaggtccgaggcagagtattacattattcttcagtgggaaaccgaggccacgggaggcgggtgtcccccttccttacttttcaggcgcatagctatttaggggcgactaataggaaaaagctcgcattttcatcgtgcctcctggagtagagaaatgggaacgcctctcccctccttgtcctttccagtgggtctcatcccttatctcgctctggtgttcacagGCGGCATGTGTTCCAGCCGACGATCGCATCTTGTGTCGCTGAGGCGGCGCAC**TGA**GGGACCAGATGGACTCCAGCCCTTCAGGAGGCAAGAGGAAAAAAGTGCTCTCGGTTCCCC

Id1 sgRNA oligonucleotide templates (t7 promoter-**20bp guide**-tracrRNA):

Id1_ATG

gcgtaatacgactcactatag**GCCTGTTCTCAGGATCATGA**gttttagagctagaaatagcaagttaaaataaggctagtccgttatcaacttgaaaaagtggcaccgagtcggtgctttt

Id1_HLH

gcgtaatacgactcactatag**GTCCTGCTCTACGACATGAA**gttttagagctagaaatagcaagttaaaataaggctagtccgttatcaacttgaaaaagtggcaccgagtcggtgctttt

**Mouse Id2 (Chromosome 12)**

CTCAGCCCCCTGTGGCGGCTCCCTCCCGGTCTTCCTCCTACGAGCAGC**ATGAAAGCCTTCAGTCCGGTGAGG**TCCGTTAGGAAAAACAGCCTGTCGGACCACAGCTTGGGCATCTCCCGGAGCAAAACCCCGGTGGACGACCCGATGAGTCTGCTCTACAACATGAACGACTG**CTACTCCAAGCTCAAGGAACTGG**TGCCCAGCATCCCCCAGAACAAGAAGGTGACCAAGATGGAAATCCTGCAGCACGTCATCGATTACATCTTGGACCTGCAGATCGCCCTGGACTCGCATCCCACTATCGTCAGCCTGCATCACCAGAGACCTGGACAGAACCAGGCGTCCAGGACGCCGCTGACCACCCTGAACACGGACATCAGCATCCTGTCCTTGCAGgtgagactagcttgcaagtacgccactgcccagacgctccgggtctcccgagctgtcactcttaaagcccatcgtagagacaggttcattaactttatttttgaggaaactgtatattgagcgtcatgtgaaatcgctacttataagttctgtgtgggttgcatctggatctgcgctgtagcatgatcctgtttcatgggacttgttggcacttttgtgaaaggaggaggggggctaccttcctttaagattacctaatatcctgccttttatcctctttctccccagGCATCTGAATTCCCTTCTGAGCTTATGTCGAATGATAGCAAAGTACTCTGTGGC**TAA**ATAAATGGTGAGTGTTGCGGGTGCCTCCTGTGTGCGCGTTTCGGTAATGTG

Id2 sgRNA oligonucleotide templates:

Id2_ATG

gcgtaatacgactcactatagg**TGAAAGCCTTCAGTCCGGTG**gttttagagctagaaatagcaagttaaaataaggctagtccgttatcaacttgaaaaagtggcaccgagtcggtgctttt

Id2_HLH

gcgtaatacgactcactatagg**CTACTCCAAGCTCAAGGAAC**gttttagagctagaaatagcaagttaaaataaggctagtccgttatcaacttgaaaaagtggcaccgagtcggtgctttt

**Mouse Id3 (Chromosome 4)**

GGTGTCTCTTTTCCTCCCTCTCT**ATCTCTACTCTCCAACATGAAGG**CGCTGAGCCCGGTGCGCGGCTGCTACGAGGCGGTGTGCTGCCTGTCGGAACGTAGCCTGGCCATTGCGCGAGGCCGCGGTAAGAGCCCGTCGACCGAGGAGCCTCTTAGCCTCTTGGACGACATGA**ACCACTGCTACTCGCGCCTGCGG**GAACTGGTGCCGGGAGTCCCGCGAGGCACTCAGCTTAGCCAGGTGGAAATCCTGCAGCGTGTCATAGACTACATCCTCGACCTTCAGGTGGTCCTGGCAGAGCCGGCGCCTGGACCCCCGGACGGTCCGCATCTCCCGATCCAGgtgcgagagggagccagaccaggctgctctgagcgtgcgggcagggatgctgcgggtcttccctatcgcgtccccgagtcccttggctaactcgtctcctaaccttctttcacagACAGCTGAGCTCACTCCGGAACTTGTGATCTCCAAGGACAAGAGGAGCTTTTGCCAC**TGA**CCCGGTCGTCCTGGCACCTCCCGGTAAGCTTTCTCCTGGCGCGGGCGAGGAGGGAGGCTTGCATGGGAAATCCTGCCTTT

Id3 sgRNA oligonucleotide templates:

Id3_ATG

gcgtaatacgactcactatagg**ATCTCTACTCTCCAACATGA**gttttagagctagaaatagcaagttaaaataaggctagtccgttatcaacttgaaaaagtggcaccgagtcggtgctttt

Id3_HLH

gcgtaatacgactcactatagg**ACCACTGCTACTCGCGCCTG**gttttagagctagaaatagcaagttaaaataaggctagtccgttatcaacttgaaaaagtggcaccgagtcggtgctttt

**Mouse Id4 (Chromosome 13)**

CTCGCTCTACCGCTTGTCGCGGTCCTCTCGCG**CAGGAAGCGCGCGATGAAGGCGG**TGAGCCCGGTGCGCCCCTCGGGCCGCAAGGCGCCGTCGGGCTGCGGCGGCGGGGAGCTGGCGCTACGCTGCCTGGCGGAGCACGGCCACAGCCTGGGTGGCTCGGCAGCCGCCGCCGCCGCTGCGGCGGCCGCGCGCTGCAAGGCGGCCGAGGCGGCGGCCGATGAGCCGGCGCTGTG**CCTGCAGTGCGATATGAACGACT**GCTACAGTCGCCTGCGGAGGCTCGTGCCTACCATCCCGCCCAACAAGAAAGTCAGCAAAGTGGAGATCCTGCAGCACGTTATCGACTACATCCTGGACCTGCAGCTGGCGCTGGAGACTCACCCTGCTTTGCTGAGACAGCCGCCACCGCCCGCGCCACCTCTCCACCCGGCCGGGGCTTGTCCGGTCGCGCCGCCGCGGACCCCACTCACCGCGCTCAACACTGACCCGgtgagaagccttggcggggcaccctgggcatcgcgggaaaggtggcggggcggcgagatacgggtggtcttgctcctctcagggaatgacagccgcttctcccgtctccaccgagagccgcctgctgggcttggtgatccactggtccctgagccgagggcggttgggacttggagccctgcgtctccggagtgtcccttgcatcacaggaggcttccccagcttcgggctcgggtggggactctgctcgcctgcctagttttccaggacgtctcctgggtggtggcgacactgtgatatgcgcactctaaccgtttttccccttggtgttgggttgctgttccagGCCGGCGCCGTGAACAAGCAGGG**TGA**CAGCATTCTCTGCCGCTGAGCTGCGATGGATGG

Id4 sgRNA oligonucleotide templates:

Id4_ATG

gcgtaatacgactcactatagg**CAGGAAGCGCGCGATGAAGG**gttttagagctagaaatagcaagttaaaataaggctagtccgttatcaacttgaaaaagtggcaccgagtcggtgctttt

Id4_HLH

gcgtaatacgactcactatagg**AGTCGTTCATATCGCACTGC**gttttagagctagaaatagcaagttaaaataaggctagtccgttatcaacttgaaaaagtggcaccgagtcggtgctttt

**Id1-4 Genotyping primers**

Id1-F CCCTCCCACCCTTTCAGTTC

Id1-R ATACTCTGCCTCGGACCTCA

Id2-F TGGAATAAGGCGGGCACAAT

Id2-R ACCTGTCTCTACGATGGGCT

Id3-F ATCAGCGCTTCCTCATTCCT

Id3-R CCCTAAACCGACTGAACCCT

Id4-F CGGTCGATTTCTGGAGCTCG

Id4-R GCAAAGCAGGGTGAGTCTCC

**Top off-targets (mismatches in red)**

1. Guide ID1 ATG CCTCC**GCCTGTTCTCAGGATCATGAAGG**TC

chr10:-41716857

CTTGGGAACTGTCAACAGTCTGGTGATGCCCAGCTCCCAGACACTAGTGCTGCGAGTAGGTACCCTACCCCCTAAATCAGAGGACCATGAGGAACTTTCATAGTCCGTGTCCCATAAAGCTTTAAGCGGTCCTGAAGGAAGACACATTAGATAGCATCCTCACCCCATGGTCAAAATCCTTCAGAGGACCTTTGCGCCTCCCAGAGTGATGGCCACACTGAGTTCTAGAAAGTTCCAGACGATGCTGCTGGCAGGATGTTCATAGCTGCTCTTTGGAAGTGGGTTTAGAGTTCAGGCAGGTCAGGGAGGCACTGCTCGTTGCTGGCCCTGGAG**GCCTGTGCTCAGGATCATGCTAG**AAAGCTATGGACAAGGCCAGGACAGCTCAGGATCGTGCTAGAAAGCTATGGACAAGGCCAGGACAGAAAGATGCCCAGGGTTCATTCAGTCCCAGTTCCCAACTTGCCCTGGCTGTGGAATCTCTCAGGAAACCAAGTGGTCTGTTCTAAAAGGCAGGACCTCCTCTGTAAAGGTCTGGACAGAAAATATTTTAGGCCCAGTGGTCCATTCCATCTCTAATATATTCAGCCCTGCCATTGGAGTGTGGGAGTAACCATTGCTGATTTATGGCTCCTGAAATTTGAACTTCGTGTGATTTTCATTTGCCCTAATGTATTATTGTTCACTTTATTT

2. Guide ID1 HLH TGAAC**GTCCTGCTCTACGACATGAACGG**CT

chr9:-109339144

TTGTCTCTTCAGACTAGCATGCCATGGAAGGAGGGATATGCTTTTTAGTAAGCATTAAGAATATTTCCCATATTTAAGTGACTGAATAAATTGCCTCTCTGTTTTCGGTTTGTTTGGAACAGGACATTTGGTAGCAAGTTTCTCTTTGCAAGACTACAAGGAGCGTGCAGAGTGGATGGGAGTCAGTGAAAAGGACACGATTGTTTGTTCAACTGGATCCTCT**CTCTTGCTCTTCGACATGAATGG**TCTCCGTCTGCAGACATTTCAGTACTGCCCAGAACAGATCTTTAGACTATGGGTGGTATGTTTGGAATGTTTTCCCCTCAGTGGTTAAAAAAAAATAGAATTAAATTTTTTAAACTTCATACTGTTGGGGTGTGCACATATGCATTTGTCTGTGTGTGAGCACTTACAGAGTTCACAGGCCAACTTCCTGGTGTTAGTCTCTTTCTTTTTCCCTTTGCACTGGCTCAGAGCACCAGGCTTGCTCAGCAAGTGTCT

3. Guide ID2 ATG CAGC**ATGAAAGCCTTCAGTCCGGTGAGG**TC

chr13:-35875834

TGCTCGGACCATGAGAGTTTGGCAGCCACTGGGGTGAACAGGTGAGAGAGAGATTAATAGTGCTGAGGCGAGATGGGATTCTACCTCACATCATTAAGATCAATATAATTTAGTGCGCAACTAGAATACACAAAAAAAAGCTGACCCCGCGGCTACTGACATCCAAGACTGTGTGTGCCAAGAGTGGAGTAAGATTCGGGAGCATTCTGACACTCTAGATTTCACTCTGGGTGTGAGGCAAAGCCTTGTTA**CTATAGCATTCAGTCCGGTGTGG**GCATGGTACTGTATCTCACCACCTACTTACTATGTTCTTACGGTTGCAGGATCTGAAACGCCTGCGGACATCATTTTGAACCACTTCCCAATGCACTCACTCACCCAGCACACTGATCCTGTCTCCCCTCACACAACTTCCTGTGTCCGTGTCTATCATGTCTCTTCTTATGAGGAACTGAAACTGGTAGAGACCCATTTCCCACTGAACAAGTGGCTGGTGTTACATACTGTTAAACTGCTTTAAAACAT

4. Guide ID2 HLH GACTG**CTACTCCAAGCTCAAGGAACTGG**TG

chr3:-154695617

ATTTGTCAAGAAAGTAATAAAAGATTCCTTAAAGAAGAAGGAAAAAAAATCCCATTTGGGGGAAGAAATTCTTAACCAAAATTTCCAGTAATGATGACTTTTGAAAACAGTGTTAATAATAGGCCCTTGTGTTAATTAAGCATTTATGCTCATAAATTGATATCATAATTAACTTACAAACAGCATCATCTCTCAGTCCTTACATCAGACAGCTTTACTTCCTGGAGTACCTTCTTTTCCAAGCCTGAATTAGCTGAGCCTCCAAAGCAGTCTTCCATGGTCACGATATGAATGCTCAGTGCATTTCCAGACTTTTTCCATCTAAGGGTTCAC**AGACTCCAAGCTTAAGGAACAAG**ATAAATGATAAATAAATGAAAGTTAGATAATAAGGCAAACAATGATGGGACCCACACTGAGACTTGGTGCTTCAGTGAAGGGAACACTTCCCTGTTCAGCCCCAGTTCAGATATCTTTTTATGCGAGTCTCATTACAGTTCACCACAACATAAGGAGTTGGGACTCAGGAACTAGGGGTAGCTTGGTTTGTTAAGAAAATCATATTGTTCACAGCTAGTAGACCTAAGTTCAACTCCCAGCCCTGGCTCAATCTAAACTTACGAAGGAGACTCACTTTATGATCCAGAATGCCCGCAGGGATCGTTGAGAAGCAGAGTCTGAGGATGGAAAG

5. Guide ID3 ATG TCTCT**ATCTCTACTCTCCAACATGAAGG**CG

chr17:-63610997

GCCCTTTGGATGCACAGACCAGGAGCAGTTCAGAGATGCGCTGTCTTATCTAGTTGCTGACTAAGGGTTGTGCTGCCGTTCCTTGAACACACGGCAGGGTCTACAGTGGAGGGAGTAGACAGCTCAGGTCTGTGGAAGCTCAGCTCAGCACTGGGAAGTTGCTAGCTACCTGTTAGGTTATTCTTGGTTACTCCACAACATTTTGTAGTAATGGAAGTACAGGGCTAAAATCTTCAGAACGTGACCTGGCGAAGGCAGACCTTCCGGATGGTCACTGGCACTGTGTCCTTTCCTTCCCGGGTCCCACAGAACCTCCTCTGTGTACATTAAGAC**ATGTCTACTCTCCACCATGAGAG**GCGAAAGCAGTGCTCAGAGAGAAAAATAAAAATAACTTTCCATGAACATGCACCCTTTAAAAAATACTCTTCACATAACTGAAAAGAGCATGCCAGAAGATTGTGTCTTTATAATATAACGATAATTGAGATGTAAAAAGAGGAAAAGCAAAACACAAAAGAAAGTGAAATAAAAAAGAGTATATGTAGGTGGCAGCAATTGCAGTAAACTTGCGGTGTCAGTTCTCTGCGTTGTTTTGTTTGTTTTGTTGTTTTGCTTTGTTTTTTCATTTTTCTTAATTCTGTGTTTCCTTTTATGAGCCTGTTCTGTTTATACGCGAGGTGAAAGGGAAAC

6. Guide ID3 HLH CATGA**ACCACTGCTACTCGCGCCTGCGG**GA

chr10:+117895989

GCTAGAAACTCTGGTTTAGGAGTCAGGAAATCCCCACAGTTAGTTTCATTAGGGCTAAAGCATTGAGATGGATGGAATTTATCTCGGGGGGTCAGCCTCCAAGGCCACCTTCAGGTGTGACCCTGTCTACATGAGATCAAGACCATAAGATGAGATCAATCTATAAGCTAATCTAGTGAAGCCAGTTTGTCATCATGCTGTTGAAAGAAATGGCCGTTCCCATGAATTCTAGTTTAATTCGTGACTCAGGGGTACCTGAGGTGAATCAGGAAAGCACAAAAGACAAACTCAGAGAAAGCAAGAGCAAAGAGGATGCCCCGTGAGTCACTTACAA**ACCACAGCTCCTCGCCCCTGAAG**TTCCTGTGACTTTCAACCAGTGCTCTCAATGCCTGAGACTTGGTGACTTGTCTATCCAGAGCATGAAGCTGTTGGACTCTTAACTGATGTGTGTGTATTAAAGAGATATGGACAACCTCATTTCATTTTGGTCTGGATAAAAGTCACAGAAACTTACAACCAATCAAAGTACAGAGAGTAAATGCCAGTGGACTCCTCAGCCATACAGGCTACATCTGCATCACCCACCCACTGAGGCTCAGGGACTACCTCATAGGAGGGGCAGAAAGAGTTAGAAATCTGGAAGAACTAGAGAGAAACATTGCTTTCTAACATGACTGGGTTACTACAGTT

7. Guide ID4 ATG TCGCG**CAGGAAGCGCGCGATGAAGGCGG**TG

chr14:+118800057

CAGCGCCAGATGAGCTAACATGAAATAAGTTCAGTTAGGTAAAAAGAATTTTAAAAATTTAAAAGCCTGAGTCAGGTCAGTGCTTAGCAATGCCTCTATAGCCAGCATCTGTTAAACCCCAACCATTGGTAATGGCATTTTGTGTTGTGTGAATTTGGCCAAGGTTGTCATCAGCACACTGCCTATGTTCAGTAACATCTGGGAAGATGGAGGAAGAACATCACATTCCGAGGCAAGCGACACTTTATATTTGAAAAGCTACTCATGAATTGCCCTCAGTGCCCTTTTAATGATCCACTTCTCCATAGAAGCATGGGCTCGTTCTCCCTCAGGC**CAGGAAGGGAGGGATGAAGGCAG**CCACCATGTTCTTCACTAATGGCTCCAGGGTCTGGCCTTGTCATAATTTACCGTCACTTCAGGACAGATGCCACTATGTCATTCCAAGGCAGATGATTAAGGTAGTTGTCCACACAATTTAGAAAAGACAAAACTATTCTTCCTCAATTATCTGCATATTATAGCTTGTCTTTTCAAATAGCTAAGTGCCGGGCAATTCTCTTTTTAGCATTTTCTTCTCTCTGAAAGTAACTGTGGGAAGGCACTAGGCCTCTGATTTAGTGCCCACACTAATAACATCTCAGCCCAGGATCTCATGTTCTGACATCCAGAAAACATATTCAACGAGGATTA

8. Guide ID4 HLH GTAGC**AGTCGTTCATATCGCACTGCAGG**CA

chr16:+89316753

CAAATTGTAAGATTGAAAATTAGAGTTCTGGGGCTGGAGATGGCTCTGCAGTTAAGAGCATGTGTTGTTCTTCCTGGTTGTGAGTTCAATTCCCAGCAACCACATCATGGGTTGCAGCCATATATAAGAGGAACAAATGCCCTCTCTCTCAAGCTGACATGCATAAAGACAGTGTACTCATATACATTAAATAAAAAAAGAAAAGTTGGAGTCCTATACTTTTGCATACCCAATCTTGATGGTATAGATAGCCCATGCATATAGTAACAATAAACTTAGGACATCTGGTATTAACTTTATACCCTATATTCATACCTCAGGACTTTTCCATAGC**AGTCATTGATATTGCACTGCAGG**TCATTGTGATAGCTGCTACAGCTATGAACATCCAGGCTATGAAACGGTGAAAAGCCTGGAACAAGTTAAAGAACAGTTACAACCGAGCTGATTTTTTTTTCTGACACACACATGTTACTGTGATTGGACTTTCCTGTGGCTTCTATTAAAGATCTCAAGGTAAAACAGCTACCTGAGGATTCATTCATCAGATCACTTGCAAAAGATAAGGGAGCCTGGTTGGTTTTTTTGTTTGTTTTTTTGTTTTTTTGTTTTTGTTTTTTTTTTTTTTTTGCATGCACTGTTCACAGCCATAGTTCCCTCTCTGCTGCTCTGTAGCCTCTCTGTGCTGGT

**Off-target genotyping primers**

ID1 ATG OT1 F AGCTGCTCTTTGGAAGTGGG

ID1 ATG OT1 R GCATCTTTCTGTCCTGGCCT

ID1 HLH OT1 F CAAGACTACAAGGAGCGTGC

ID1 HLH OT1 R ACATTCCAAACATACCACCCA

ID2 ATG OT1 F TTCGGGAGCATTCTGACACT

ID2 ATG OT1 R AGTTGTGTGAGGGGAGACAG

ID2 HLH OT1 F AGCCTGAATTAGCTGAGCCT

ID2 HLH OT1 R ATCTGAACTGGGGCTGAACA

ID3 ATG OT1 F GCACTGTGTCCTTTCCTTCC

ID3 ATG OT1 R GACACCGCAAGTTTACTGCA

ID3 HLH OT1 F GCTGTTGAAAGAAATGGCCGT

ID3 HLH OT1 R TCAGGCATTGAGAGCACTGG

ID4 ATG OT1 F CATCACATTCCGAGGCAAGC

ID4 ATG OT1 R GACCCTGGAGCCATTAGTGA

ID4 HLH OT1 F GCCCTCTCTCTCAAGCTGAC

ID4 HLH OT1 R CCAGGCTTTTCACCGTTTCA

**Detailed genotype information of embryos presented in Figure 6**

# = mutant number

**[target site 1 indel; target site 2 indel]** = 1 allele

- = deletion, + = insertion

* = **ATG** translational start site disrupted

fs = frame-shift causing indel

nc = non-coding indel

inf = in frame indel

sd = splice donor site deletion

STOP = early stop codon generated (indicated when 2^nd^ target site puts sequence back in frame, or if present in inserts)

wt = wild type reference

**A1** = allele 1

**A**/**T**/**C**/**G** = inserted bases

xxx = deleted bases

**NGG** / **CCN** = PAM sequence

TGA = early stop codon

**#3 ID1 [-203*] [-248*]**

wtTGTTCTCAGCCTCCTCCGCTCCCCTCCGCCTGTTCTCAGGATC**ATG**A**AGG**TCGCCAGTGGCAGTGCCGCAGCCGCTGCAGGCCCTAGCTGTTCGCTGAAGGCGGGCAGGACAGCGGGCGAGGTGGTACTTGGTCTGTCGGAGCAAAGCGTGGCCATCTCGCGCTGCGCTGGGACGCGCCTGCCCGCCTTGCTGGACGAGCAGCAGGTGAACGTCCTGCTCTACGACATGAA**CGG**CTGCTACTCACGCCTCAAGGAGCTGGTGCCCACCCTGCCCCAGAACCGCAAAGT

**A1**TGTTCTCAGCCTCCTCCGCTCCCCTCCGCCTGTxxxxxxxxxxxxxxxxxxxxxxxxxxxxxxxxxxxxxxxxxxxxx203xxxxxxxxxxxxxxxxxxxxxxxxxxxxxxxxxxxxxxxxxxxxxxxxxxxxxxxxxxxxxxxxxxxxxxxxxxxxxxxxxxxxxxxxxxxxxxxxxxxxxxxxxxxxxxxxxxxxxxxxxxxxxxxxxxxxxxxxxxxxxxxxxxxxxxxxxxxGCTACTCACGCCTCAAGGAGCTGGTGCCCACCCTGCCCCAGAACCGCAAAGT

**A2**CTCAAAGCCACGCCTCTAGCAGCCTATTGGTTGCTTTTGAACGTTCTGAACCCGCCCTTGGTCTGCCCTTATAAAAGACTGGCTCCAGCGCTCTGCCCTCTCATTGTACAACCTTTCCTCAACTTCTTGTTxxxxxxxxxxxxxxxxxxxxxxxxxxxxxxxxxxxxxxxxxxxxxxxxxxxxxxxxxxxxxxxxxxxxxxxx248xxxxxxxxxxxxxxxxxxxxxxxxxxxxxxxxxxxxxxxxxxxxxxxxxxxxxxxxxxxxxxxxxxxxxxxxxxxxxxxxxxxxxxxxxxxxxxxxxxxxxxxxxxxxxxxxxxxxxxxxxxxxxxxxxxxxxxxxxxxxxxxxxxxxxxxxxxxxxxxxxxxxxxxxxxxxxCTGCTACTCACGCCTCAAGGAGCTGGTGCCCACCCTGCCCCAGAACCGCAAAGT

wtCTCAAAGCCACGCCTCTAGCAGCCTATTGGTTGCTTTTGAACGTTCTGAACCCGCCCTTGGTCTGCCCTTATAAAAGACTGGCTCCAGCGCTCTGCCCTCTCATTGTACAACCTTTCTTCAACTTCTTGTTCTCTTCCCACACTCTGTTCTCAGCCTCCTCCGCTCCCCTCCGCCTGTTCTCAGGATC**ATG**A**AGG**TCGCCAGTGGCAGTGCCGCAGCCGCTGCAGGCCCTAGCTGTTCGCTGAAGGCGGGCAGGACAGCGGGCGAGGTGGTACTTGGTCTGTCGGAGCAAAGCGTGGCCATCTCGCGCTGCGCTGGGACGCGCCTGCCCGCCTTGCTGGACGAGCAGCAGGTGAACGTCCTGCTCTACGACATGAA**CGG**CTGCTACTCACGCCTCAAGGAGCTGGTGCCCACCCTGCCCCAGAACCGCAAAGT

**#3 ID2 [-135inf] [-10fs; -1] [-10fs; -4]**

wtCTCAGCCCCCTGTGGCGGCTCCCTCCCGGTCTTCCTCCTACGAGCAGC**ATG**AAAGCCTTCAGTCCGGTG**AGG**TCCGTTAGGAAAAACAGCCTGTCGGACCACAGCTTGGGCATCTCCCGGAGCAAAACCCCGGTGGACGACCCGATGAGTCTGCTCTACAACATGAACGACTGCTACTCCAAGCTCAAGGAAC**TGG**TGCCCAGCATCCCCCAGAACAAGAAGGTGACCAAGATGGAAATCCTGCAGCACGTCATCGATTACATCTTGGACCTGCAGATCGCCCTGGAC

**A1**CTCAGCCCCCTGTGGCGGCTCCCTCCCGGTCTTCCTCCTACGAGCAGCATGAAAGCCTTCAGTxxxxxxxxxxxxxxxxxxxxxxxxxxxxxxxxxxx135xxxxxxxxxxxxxxxxxxxxxxxxxxxxxxxxxxxxxxxxxxxxxxxxxxxxxxxxxxxxxxxxxxxnxxxxxxxxxxxxxxxxxxxxxxxxxxxxxCCCAGCATCCCCCAGAACAAGAAGGTGACCAAGATGGAAATCCTGCAGCACGTCATCGATTACATCTTGGACCTGCAGATCGCCCTGGAC

**A2**CTCAGCCCCCTGTGGCGGCTCCCTCCCGGTCTTCCTCCTACGAGCAGC**ATG**AAAGCCTTCAxxxx10xxxx**G**TCCGTTAGGAAAAACAGCCTGTCGGACCACAGCTTGGGCATCTCCCGGAGCAAAACCCCGGTGGACGACCCGATGAGTCTGCTCTACAACATGAACGACTGCTACTCCAAGCTCAAxGAAC**TGG**TGCCCAGCATCCCCCAGAACAAGAAGGTGACCAAGATGGAAATCCTGCAGCACGTCATCGATTACATCTTGGACCTGCAGATCGCCCTGGAC

**A3**CTCAGCCCCCTGTGGCGGCTCCCTCCCGGTCTTCCTCCTACGAGCAGC**ATG**AAAGCCTTCAxxxx10xxxx**G**TCCGTTAGGAAAAACAGCCTGTCGGACCACAGCTTGGGCATCTCCCGGAGCAAAACCCCGGTGGACGACCCGATGAGTCTGCTCTACAACATGAACGACTGCTACTCCAAGCTCxxxxAAC**TGG**TGCCCAGCATCCCCCAGAACAAGAAGGTGACCAAGATGGAAATCCTGCAGCACGTCATCGATTACATCTTGGACCTGCAGATCGCCCTGGAC

**#3 Id3 [-371*] [+1*; -6] [+1nc; +397-108 STOP]**

wtTCTATAAGAGTCGGCCGCTGCAGGCGTGCGCGCACTGTTTGCTGCTTTAGGTGTCTCTTTTCCTCCCTCTCTATCTCTACTCTCCAAC**ATG**A**AGG**CGCTGAGCCCGGTGCGCGGCTGCTACGAGGCGGTGTGCTGCCTGTCGGAACGTAGCCTGGCCATTGCGCGAGGCCGCGGTAAGAGCCCGTCGACCGAGGAGCCTCTTAGCCTCTTGGACGACATGAACCACTGCTACTCGCGCCTG**CGG**GAACTGGTGCCGGGAGTCCCGCGAGGCACTCAGCTTAGCCAGGT

**A1**CCTCGGCGTCAGACCAGCCTAAGGAAGCCxxxxxxxxxxxxxxxxxxxxxxxxxxxxx371xxxxxxxxxxxxxxxxxxxxxxxxxxxxxxxxxxxxxxxxxxxxxxxxxxxxxxxxxxxxxxxxxxxxxxxxxxxxxxxxxxxxxxxxxxxxxxxxxxxxxxxxxxxxxxxxxxxxxxxxxxxxxxxxxxxxxxxxxxxxxxxxxxxxxxxxxxxxxxxxxxxxxxxxxxxxxxxxxxxxxxxxxxxxxxxxxxxxxxxxxxxxxxxxxxxxxxxxxxxxxxxxxxxxxxxxxxxxxxxxxxxxxxxxxxxxxxxxxxxxxxxxxxxxxxxxxxxxxxxxxxxxxxxxxxxxxxxxxxxxxxxxxxxxxxxxxxxxxxxxxxxxxxxxxxxxxxxxxxxGGGAGTCCCGCGAGGCACTCAGCTTAGCCAGGT

**↓G**

**A2**TCTATAAGAGTCGGCCGCTGCAGGCGTGCGCGCACTGTTTGCTGCTTTAGGTGTCTCTTTTCCTCCCTCTCTATCTCTACTCTCCAAC**ATG**A**AGG**CGCTGAGCCCGGTGCGCGGCTGCTACGAGGCGGTGTGCTGCCTGTCGGAACGTAGCCTGGCCATTGCGCGAGGCCGCGGTAAGAGCCCGTCGACCGAGGAGCCTCTTAGCCTCTTGGACGACATGAACCACTGCTACTCGxx6xxx**CGG**GAACTGGTGCCGGGAGTCCCGCGAGGCACTCAGCTTAGCCAGGT

**↓A**

**A3**TCTATAAGAGTCGGCCACTGCAGGCGTGCGCGCACTGTTTGCTGCTTTAGGTGTCTCTTTTCCTCCCTCTCTATCTCTACTCTCCAAC**ATG**A**AGG**CGCTGAGCCCGGTGCGCGGCTGCTACGAGGCGGTGTGCTGCCTGTCGGAACGTAGCCTGGCCATTGCGCGAGGCCGCGGTAAGAGCCCGTCGACCGAGGAGCCTCTTAGCCTCTTGGACGACATGAACCACTGCxxxxxxxxxxxxxxxxxxxxx108xxxxxxxxxxxxxxxxxxxxxxxxxxxxxxxxxxxxxxxxxxxxxxxxxxxxxxxxxxxxxxxxxxxxxxxxxxxxxxxxxxxxCTG

**↑**

**CAGGACTGAAGGGGTCATGCAGTGTTTTGGAGATGCCAGTACTATGAGATGACCACCAAGAGCAGCAGCAGCAGTGGAGTACAGGCATCTGGAGCCTAGAGGATGACCTGTGTGCTACAAAGGGCCTGGCTGGAGAAGTGACCCAAGCCCTTGGAGGAGCCCAGAAGATCGTGAGTTGGATCCCAGACATTGGACGGTTAGAGATTGACTTTTGCTTTTGATTGTGACTGTGCCCTGATATTTTCCCTCTTGAAGGAAGAAACTGTTTTAGTGGAGCCCACAGTTAAGAGACTTTTAATTGTAAAAAGACTTTGGATTTTAAAAGAGATGGACATTTTAAAGAGTTTGAAATTTTAAGAATATGTAAAGACTGTGGGACTTTTAAAGTTATTTAGAT**

**#3 Id4 [-30*; 0] [+1-5*; 0]**

wtCGCTCTACCGCTTGTCGCGGTCCTCTCGCGCAGGAAGCGCGCG**ATG**AAGG**CGG**TGAGCCCGGTGCGCCCCTCGGGCCGCAAGGCGCCGTCGGGCTGCGGCGGCGGGGAGCTGGCGCTACGCTGCCTGGCGGAGCACGGCCACAGCCTGGGTGGCTCGGCAGCCGCCGCCGCCGCTGCGGCGGCCGCGCGCTGCAAGGCGGCCGAGGCGGCGGCCGATGAGCCGGCGCTGTG**CCT**GCAGTGCGATATGAACGACTGCTACAGTCGCCTGCGGAGGCTCGTGCCTACCAT

**A1**CGCTCTACCGCTTGTCGCGGTCCTCTCGCGCAGGAAGCGxxxxx**TG**xxxx30xxxxxxxxxxxxxxxxxxxCGGGCCGCAAGGCGCCGTCGGGCTGCGGCGGCGGGGAGCTGGCGCTACGCTGCCTGGCGGAGCACGGCCACAGCCTGGGTGGCTCGGCAGCCGCCGCCGCCGCTGCGGCGGCCGCGCGCTGCAAGGCGGCCGAGGCGGCGGCCGATGAGCCGGCGCTGTG**CCT**GCAGTGCGATATGAACGACTGCTACAGTCGCCTGCGGAGGCTCGTGCCTACCAT

**G↓**

**A2**CGCTCTACCGCTTGTCGCGGTCCTCTCGCGCAGGAAGCGCxxx**A**x**G**xAGG**CGG**TGAGCCCGGTGCGCCCCTCGGGCCGCAAGGCGCCGTCGGGCTGCGGCGGCGGGGAGCTGGCGCTACGCTGCCTGGCGGAGCACGGCCACAGCCTGGGTGGCTCGGCAGCCGCCGCCGCCGCTGCGGCGGCCGCGCGCTGCAAGGCGGCCGAGGCGGCGGCCGATGAGCCGGCGCTGTG**CCT**GCAGTGCGATATGAACGACTGCTACAGTCGCCTGCGGAGGCTCGTGCCTACCAT

**-------------------------------------------------------------------------------------------------------------------------------------------------**

**#9 ID1 [-3*; -21] [-194*]**

wtTGTTCTCAGCCTCCTCCGCTCCCCTCCGCCTGTTCTCAGGATC**ATG**A**AGG**TCGCCAGTGGCAGTGCCGCAGCCGCTGCAGGCCCTAGCTGTTCGCTGAAGGCGGGCAGGACAGCGGGCGAGGTGGTACTTGGTCTGTCGGAGCAAAGCGTGGCCATCTCGCGCTGCGCTGGGACGCGCCTGCCCGCCTTGCTGGACGAGCAGCAGGTGAACGTCCTGCTCTACGACATGAA**CGG**CTGCTACTCACGCCTCAAGGAGCTGGTGCCCACCCTGCCCCAGAACCGCAAAGT

**A1**TGTTCTCAGCCTCCTCCGCTCCCCTCCGCCTGTTCTCAGGATC**A**xxx**AGG**TCGCCAGTGGCAGTGCCGCAGCCGCTGCAGGCCCTAGCTGTTCGCTGAAGGCGGGCAGGACAGCGGGCGAGGTGGTACTTGGTCTGTCGGAGCAAAGCGTGGCCATCTCGCGCTGCGCTGGGACGCGCCTGCCCGCCTTGCTGGACGAGCAGCAGGxxxxxxxx21xxxxxxxxxxxTGAA**CGG**CTGCTACTCACGCCTCAAGGAGCTGGTGCCCACCCTGCCCTAGAAC

**A2**TGTTCTCAGCCTCCTCCGCTCCCCTCCGCCTGTTCTCAGGxxxxxxxxxxxxxxxxxxxxxxxxxxxxxxxxxxx194xxxxxxxxxxxxxxxxxxxxxxxxxxxxxxxxxxxxxxxxxxxxxxxxxxxxxxxxxxxxxxxxxxxxxxxxxxxxxxxxxxxxxxxxxxxxxxxxxxxxxxxxxxxxxxxxxxxxxxxxxxxxxxxxxxxxxxxxxxxxxxxxxxxxxxxxxxxxCTGCTACTCACGCCTCAAGGAGCTGGTGCCCACCCTGCCCCAGAACCGCAAAGT

**#9 ID2 [-5inf STOP; -55bif] [0; -55fs] [-5fs; -15] [0; -15inf]**

wtCTCAGCCCCCTGTGGCGGCTCCCTCCCGGTCTTCCTCCTACGAGCAGC**ATG**AAAGCCTTCAGTCCGGTG**AGG**TCCGTTAGGAAAAACAGCCTGTCGGACCACAGCTTGGGCATCTCCCGGAGCAAAACCCCGGTGGACGACCCGATGAGTCTGCTCTACAACATGAACGACTGCTACTCCAAGCTCAAGGAAC**TGG**TGCCCAGCATCCCCCAGAACAAGAAGGTGACCAAGATGGAAATCCTGCAGCACGTCATCGATTACATCTTGGACCTGCAGATCGCCCTGGAC

**A1**CTCAGCCCCCTGTGGCGGCTCCCTCCCGGTCTTCCTCCTACGAGCAGC**ATG**AAAGCCTTCAxxxxxGTG**AGG**TCCGTTAGGAAAAACAGCCTGTCGGACCACAGCTTGGGCATCTCCCGGAGCAAAACCCCGGTGxxxxxxxxxxxxxxxxxxxxxxxxxxx55xxxxxxxxxxxxxxxxxxxxxxxxxxAAC**TGG**TGCCCAGCATCCCCCAGAACAAGAAGGTGACCAAGATGGAAATCCTGCAGCACGTCATCGATTACATCTTGGACCTGCAGATCGCCCTGGAC

**A2**CTCAGCCCCCTGTGGCGGCTCCCTCCCGGTCTTCCTCCTACGAGCAGC**ATG**AAAGCCTTCAGTCCGGTG**AGG**TCCGTTAGGAAAAACAGCCTGTCGGACCACAGCTTGGGCATCTCCCGGAGCAAAACCCCGGTGxxxxxxxxxxxxxxxxxxxxxxxxxxx55xxxxxxxxxxxxxxxxxxxxxxxxxxAAC**TGG**TGCCCAGCATCCCCCAGAACAAGAAGGTGACCAAGATGGAAATCCTGCAGCACGTCATCGATTACATCTTGGACCTGCAGATCGCCCTGGAC

**A3**CTCAGCCCCCTGTGGCGGCTCCCTCCCGGTCTTCCTCCTACGAGCAGC**ATG**AAAGCCTTCAxxxxxGTG**AGG**TCCGTTAGGAAAAACAGCCTGTCGGACCACAGCTTGGGCATCTCCCGGAGCAAAACCCCGGTGGACGACCCGATGAGTCTGCTCTACAACATGAACGACTGCTACTCCAAGCTCAxxxxxxx15xxxxxxGCATCCCCCAGAACAAGAAGGTGACCAAGATGGAAATCCTGCAGCACGTCATCGATTACATCTTGGACCTGCAGATCGCCCTGGAC

**A4**CTCAGCCCCCTGTGGCGGCTCCCTCCCGGTCTTCCTCCTACGAGCAGC**ATG**AAAGCCTTCAGTCCGGTG**AGG**TCCGTTAGGAAAAACAGCCTGTCGGACCACAGCTTGGGCATCTCCCGGAGCAAAACCCCGGTGGACGACCCGATGAGTCTGCTCTACAACATGAACGACTGCAACTCCAAGCTCAxxxxxxx15xxxxxxGCATCCCACAGAACAAGAAGGTGACCAAGATGGAAATCCTGCAGCACGTCATCGATTACATCTTGGACCTGCAGATCGCCCTGGAC

**#9 ID3 [-10*; +1] [-10*; 0] [-6*; 0] [-151*] [-148*] [-6*; +1]**

wtTCTATAAGAGTCGGCCGCTGCAGGCGTGCGCGCACTGTTTGCTGCTTTAGGTGTCTCTTTTCCTCCCTCTCTATCTCTACTCTCCAAC**ATG**A**AGG**CGCTGAGCCCGGTGCGCGGCTGCTACGAGGCGGTGTGCTGCCTGTCGGAACGTAGCCTGGCCATTGCGCGAGGCCGCGGTAAGAGCCCGTCGACCGAGGAGCCTCTTAGCCTCTTGGACGACATGAACCACTGCTACTCGCGCCTG**CGG**GAACTGGTGCCGGGAGTCCCGCGAGGCACTCAGCTTAGCCAGGT

**A1**TCTATAAGAGTCGGCCGCTGCAGGCGTGCGCGCACTGTTTGCTGCTTTAGGTGTCTCTTTTCCTCCCTCTCTATCTCTACTCTCCAACxxxx10xxxxTGAGCCCGGTGCGCGGCTGCTACGAGGCGGTGTGCTGCCTGTCGGAACGTAGCCTGGCCATTGCGCGAGGCCGCGGTAAGAGCCCGTCGACCGAGGAGCCTCTTAGCCTCTTGGACGACATGAACCACTGCTACTCGCGCCTG**CGG**GAACTGGTGCCGGGAGTCCCGCGAGGCACTCAGCTTAGCCAGGT

**T↑**

**A2**TCTATAAGAGTCGGCCGCTGCAGGCGTGCGCGCACTGTTTGCTGCTTTAGGTGTCTCTTTTCCTCCCTCTCTATCTCTACTCTCCAACxxxx10xxxxTGAGCCCGGTGCGCGGCTGCTACGAGGCGGTGTGCTGCCTGTCGGAACGTAGCCTGGCCATTGCGCGAGGCCGCGGTAAGAGCCCGTCGACCGAGGAGCCTCTTAGCCTCTTGGACGACATGAACCACTGCTACTCGCGCCTG**CGG**GAACTGGTGCCGGGAGTCCCGCGAGGCACTCAGCTTAGCCAGGT

**A3**TCTATAAGAGTCGGCCGCCGCAGGCGTGCGCGCACTGTTTGCTGCTTTAGGTGTCTCTTTTCCTCCCTTTCTATCTCTACTCTCCAxxx6xx**AGG**CGCTGAGCCCGGTGCGCGGCTGCTACGAGGCGGTGTGCTGCCTGTCGGAACGTAGCCTGGCCATTGCGCGAGGCCGCGGTAAGAGCCCGTCGACCGAGGAGCCTCTTAGCCTCTTGGACGACATGAACCACTGCTACTCGCGCCTG**CGG**GAACTGGTGCCGGGAGTCCCGCGAGGCACTCAGCTTAGCCAGGT

**A4**TCTATAAGAGTCGGCCGCTGCAGGCGTGCGCGCACTGTTTGCTGCTTTAGGTGTCTCTTTTCCTCCCTCTCTATCTCTACTCTCCAACxxxxxxxxxxxxxxxxxxxxxxxx151xxxxxxxxxxxxxxxxxxxxxxxxxxxxxxxxxxxxxxxxxxxxxxxxxxxxxxxxxxxxxxxxxxxxxxxxxxxxxxxxxxxxxxxxxxxxxxxxxxxxxxxxxxxxxxxxxxxxxxxxxxxxTG**CGG**GAACTGGTGCCGGGAGTCCCGCGAGGCACTCAGCTTAGCCAGGT

**A5**TCTATAAGAGTCGGCCGCTGCAGGCGTGCGCGCACTGTTTGCTGCTTTAGGTGTCTCTTTTCCTCCCTCTCTATCTCTACTCTCCAAC**A**xxxxxxxxxxxxxxxxxxxxxxxx148xxxxxxxxxxxxxxxxxxxxxxxxxxxxxxxxxxxxxxxxxxxxxxxxxxxxxxxxxxxxxxxxxxxxxxxxxxxxxxxxxxxxxxxxxxxxxxxxxxxxxxxxxxxxxxxxxxxxxxxxxCCTG**CGG**GAACTGGTGCCGGGAGTCCCGCGAGGCACTCAGCTTAGCCAGGT

**A6**TCTATAAGAGTCGGCCGCTGCAGGCGTGCGCGCACTGTTTGCTGCTTTAGGTGTCTCTTTTCCTCCCTCTCTATCTCTACTCTCCAxxx6xx**AGG**CGCTGAGCCCGGTGCGCGGCTGCTACGAGGCGGTGTGCTGCCTGTCGGAACGTAGCCTGGCCATTGCGCGAGGCCGCGGTAAGAGCCCGTCGACCGAGGAGCCTCTTAGCCTCTTGGACGACATGAACCACTGCTACTCGCGCCTG**CGG**GAACTGGTGCCGGGAGTCCCGCGAGGCACTCAGCTTAGCCAGGT

**T↑**

**#9 ID4 [-1fs; 0]**

wtCGCTCTACCGCTTGTCGCGGTCCTCTCGCGCAGGAAGCGCGCG**ATG**AAGG**CGG**TGAGCCCGGTGCGCCCCTCGGGCCGCAAGGCGCCGTCGGGCTGCGGCGGCGGGGAGCTGGCGCTACGCTGCCTGGCGGAGCACGGCCACAGCCTGGGTGGCTCGGCAGCCGCCGCCGCCGCTGCGGCGGCCGCGCGCTGCAAGGCGGCCGAGGCGGCGGCCGATGAGCCGGCGCTGTG**CCT**GCAGTGCGATATGAACGACTGCTACAGTCGCCTGCGGAGGCTCGTGCCTACCAT

**A1**CGCTCTACCGCTTGTCGCGGTCCTCTCGCGCAGGAAGCGCGCG**ATG**xAGG**CGG**TGAGCCCGGTGCGCCCCTCGGGCCGCAAGGCGCCGTCGGGCTGCGGCGGCGGGGAGCTGGCGCTACGCTGCCTGGCGGAGCACGGCCACAGCCTGGGTGGCTCGGCAGCCGCCGCCGCCGCTGCGGCGGCCGCGCGCTGCAAGGCGGCCGAGGCGGCGGCCGATGAGCCGGCGCTGTG**CCT**GCAGTGCGATATGAACGACTGCTACAGTCGCCTGCGGAGGCTCGTGCCTACCAT

**-------------------------------------------------------------------------------------------------------------------------------------------------**

**#17 ID1 [-1*; -20] [+9-107*; -9] [+9-107*; -20]**

wtTGTTCTCAGCCTCCTCCGCTCCCCTCCGCCTGTTCTCAGGATC**ATG**A**AGG**TCGCCAGTGGCAGTGCCGCAGCCGCTGCAGGCCCTAGCTGTTCGCTGAAGGCGGGCAGGACAGCGGGCGAGGTGGTACTTGGTCTGTCGGAGCAAAGCGTGGCCATCTCGCGCTGCGCTGGGACGCGCCTGCCCGCCTTGCTGGACGAGCAGCAGGTGAACGTCCTGCTCTACGACATGAA**CGG**CTGCTACTCACGCCTCAAGGAGCTGGTGCCCACCCTGCCCCAGAACCGCAAAGT

**A1**TGTTCTCAGCCTCCTCCGCTCCCCTCCGCCTGTTCTCAGGATC**A**x**G**A**AGG**TCGCCAGTGGCAGTGCCGCAGCCGCTGCAGGCCCTAGCTGTTCGCTGAAGGCGGGCAGGACAGCGGGCGAGGTGGTACTTGGTCTGTCGGAGCAAAGCGTGGCCATCTCGCGCTGCGCTGGGACGCGCCTGCCCGCCTTGCTGGACGAGCAGCAGGTGAACGTCCTGCTxxxxxxxx20xxxxxxxxxxACTCACGCCTCAAGGAGCTGGTGCCCACCCTGCCCCAGAACCGCAAAGT

**↓TGGTATCTC**

**A2**TGTTCTCAGCCTCCTCCGCTCCCCTCCGCCTGTTCTCAGxxxxxxxxxxxxxxxxxxxxxxxxxxxxxxxxxxxxxxxxx9x107xxxxxxxxxxxxxxxxxxxxxxxxxxxxxxxxxxxxxxxxxxxxxxxxxxxxxxxxxxxxxGCGTGGCCATCTCGCGCTGCGCTGGGACGCGCCTGCCTGCCTTGCTGGACGAGCAGCAGGTGAACGTCCTGCTCTxx9xxxxxxA**CGG**CTGCTACTCACGCCTCAAGGAGCTGGTGCCCACCCTGCCCCAGAACCGCAAAGT

**↓TGGTATCTC**

**A3**TGTTCTCAGCCTCCTCCGCTCCCCTCCGCCTGTTCTCAGxxxxxxxxxxxxxxxxxxxxxxxxxxxxxxxxxxxxxxxxx9x107xxxxxxxxxxxxxxxxxxxxxxxxxxxxxxxxxxxxxxxxxxxxxxxxxxxxxxxxxxxxxGCGTGGCCATCTCGCGCTGCGCTGGGACGCGCCTGCCCGCCTTGCTGGACGAGCAGCAGGTGAACGTCCTGCTxxxxxxxx20xxxxxxxxxxACTCACGCCTCAAGGAGCTGGTGCCCACCCTGCCCCAGAACCGCAAAGT

**#17 ID2 [-10fs; -81] [-10fs; -81] [+1-2fs; -81] [-5fs; -3]**

wtCTCAGCCCCCTGTGGCGGCTCCCTCCCGGTCTTCCTCCTACGAGCAGC**ATG**AAAGCCTTCAGTCCGGTG**AGG**TCCGTTAGGAAAAACAGCCTGTCGGACCACAGCTTGGGCATCTCCCGGAGCAAAACCCCGGTGGACGACCCGATGAGTCTGCTCTACAACATGAACGACTGCTACTCCAAGCTCAAGGAAC**TGG**TGCCCAGCATCCCCCAGAACAAGAAGGTGACCAAGATGGAAATCCTGCAGCACGTCATCGATTACATCTTGGACCTGCAGATCGCCCTGGAC

**A1**CTCAGCCCCCTGTGGCGGCTCCCTCCCGGTCTTCCTCCTACGAGCAGC**ATG**AAAGCCTTCAGxxxx10xxxxTCCGTTAGGAAAAACAGCCTGTCGGACCACAGCTTGGGCATCTCCCGGAGCAAAACCCxxxxxxxxxxxxxxxxxxxxxxxxxxxxxxxxxxxxxxxxxxxxxxxxxx81xxxxxxxxxxxxxxxxxxxxxxxxxxxxxAGAACAAGAAGGTGACCAAGATGGAAATCCTGCAGCACGTCATCGATTACATCTTGGACCTGCAGATCGCCCTGGAC

**A2**CTCAGCCCCCTGTGGCGGCTCCCTCCCGGTCTTCCTCCTACGAGCAGC**ATG**AAAGCCTxxxxxCCxxxxx**GG**TCCGTTAGGAAAAACAGCCGATCGGACCACAGCTTGGGCATCTCCCGGAGCACAACCCxxxxxxxxxxxxxxxxxxxxxxxxxxxxxxxxxxxxxxxxxxxxxxxxxx81xxxxxxxxxxxxxxxxxxxxxxxxxxxxxAGAACAAGAAGGTGACCAAGATGGAAATCCTGCAGCACGTCATCGATTACATCTTGGACCTGCAGATCGCCCTGG

**↓A**

**A3**CTCAGCCCCCTGTGGCGGCTCCCTCCCGGTCTTCCTCCTACGAGCAGC**ATG**AAAGCCTTCAGTCxxGTG**AGG**TCCGTTAGGAAAAACAGCCTGTCGGACCACAGCTTGGGCATCTCCCGGAGCAAAACCCxxxxxxxxxxxxxxxxxxxxxxxxxxxxxxxxxxxxxxxxxxxxxxxxxx81xxxxxxxxxxxxxxxxxxxxxxxxxxxxxAGAACAAGAAGGTGACCAAGATGGAAATCCTGCAGCACGTCATCGATTACATCTTGGACCTGCAGATCGCCCTGGA

**A4**CTCAGCCCCCTGTGGCGGCTCCCTCCCGGTCTTCCTCCTACGAGCAGC**ATG**AAAGCCTTCAxxxxxGTG**AGG**TCCGTTAGGAAAAACAGCCTGTCGGACCACAGCTTGGGCATCTCCCGGAGCAAAACCCCGGTGGACGACCCGATGAGTCTGCTCTACAACATGAACGACTGCTACTCCAAGCTCAAxxxAC**TGG**TGCCCAGCATCCCCCAGAACAAGAAGGTGACCAAGATGGAAATCCTGCAGCACGTCATCGATTACATCTTGGACCTGCAGATCGCCCTGGAC

**#17 ID3 [-9*; -43] [-149*] [-75*; +2] [-75*; 0] [-2*; 0] [-97*; -7]**

wtTCTATAAGAGTCGGCCGCTGCAGGCGTGCGCGCACTGTTTGCTGCTTTAGGTGTCTCTTTTCCTCCCTCTCTATCTCTACTCTCCAAC**ATG**A**AGG**CGCTGAGCCCGGTGCGCGGCTGCTACGAGGCGGTGTGCTGCCTGTCGGAACGTAGCCTGGCCATTGCGCGAGGCCGCGGTAAGAGCCCGTCGACCGAGGAGCCTCTTAGCCTCTTGGACGACATGAACCACTGCTACTCGCGCCTG**CGG**GAACTGGTGCCGGGAGTCCCGCGAGGCACTCAGCTTAGCCAGGT

**A1**TCTATAAGAGTCGGCCGCTGCAGGCGTGCGCGCACTGTTTGCTGCTTTAGGTGTCTCTTTTCCTCCCTCTCTATCTCTACTCTCxAxx9xxxxx**G**CGCTGAGCCCGGTGCGCGGCTGCTACGAGGCGGTGTGCTGCCTGTCGGAACGTAGCCTGGCCATTGCGCGAGGCCGCGGTAAGAGCCCGTCGACCGAGGAGCCTCTTAGCxxxxxxxxxxxxxxxxxxxxx43xxxxxxxxxxxxxxxxxxxxTGGTGCCGGGAGTCCCGCGAGGCACTCAGCTTAGCCAGGT

**A2**TCTATAAGAGTCGGCCGCTGCAGGCGTGCGCGCACTGTTTGCTGCTTTAGGTGTCTCTTTTCCTCCCTCTCTATCTCTACTCTCCAAC**A**xxxxxxxxxxxxxxxxxxxxxxxxxxxxxxxxxx149xxxxxxxxxxxxxxxxxxxxxxxxxxxxxxxxxxxxxxxxxxxxxxxxxxxxxxxxxxxxxxxxxxxxxxxxxxxxxxxxxxxxxxxxxxxxxxxxxxxxxxxxxxxxxxxxCTG**CGG**GAACTGGTGCCGGGAGTCCCGCGAGGCACTCAGCTTAGCCAGGT

**A3**TCTATAAGAGTCGGCCGCTGCAGGCGTGCGCGCACTGTTTGCTGCTTTAGGTGTCTCTTTTCCTCCCTCTCTATCTCTACTCTCCxxxxxxxxxxxxxxxxxxxxxxxxxxxxxxxxxxx75xxxxxxxxxxxxxxxxxxxxxxxxxxxxxxxxxxxxxxGCGCGAGGCCGCGGTAAGAGCCCGTCGACCGAGGAGCCTCTTAGCCTCTTGGACGACATGAACCACTGCTACTCGCGCCTG**CGG**GAACTGGTGCCGGGAGTCCCGCGAGGCACTCAGCTTAGCCAGGT

**TA↑**

**A4**TCTATAAGAGTCGGCCGCTGCAGGCGTGCGCGCACTGTTTGCTGCTTTAGGTGTCTCTTTTCCTCCCTCTCTATCTCTACTCTCCxxxxxxxxxxxxxxxxxxxxxxxxxxxxxxxxxxx75xxxxxxxxxxxxxxxxxxxxxxxxxxxxxxxxxxxxxxGCGCGAGGCCGCGGTAAGAGCCCGTCGACCGAGGAGCCTCTTAGCCTCTTGGACGACATGAACCACTGCTACTCGCGCCTG**CGG**GAACTGGTGCCGGGAGTCCCGCGAGGCACTCAGCTTAGCCAGGT

**A5**TCTATAAGAGTCGGCCGCTGCAGGCGTGCGCGCACTGTTTGCTGCTTTAGGTGTCTCTTTTCCTCCCTCTCTATCTCTACTCTCCAAC**A**xxA**AGG**CGCTGAGCCCGGTGCGCAGCTGCTACGAGGCGGTGTGCTGCCTGTCGGAACGTAGCCTGGCCATTGCGCGGGGCCGCGGTAAGAGCCCGTCGACCGAGGAGCCTCTTAGCCTCTTGGACGACATGAACCACTGCTACTCGCGCCTG**CGG**GAACTGGTGCCGGGAGTCCCGCGAGGCACTCAGCTTAGCCAGGT

**A6**CCTCGGCGTCAGACCAGCCTAAGGAAGCCTGTTAGCAATTTAAACCCACTGTGAACGCCCAGGACCGGGGAGGCGGTGCCCAGGGCGGGCGGGGGTGGACCCTTGGCGGTCTGTTTTGAATAAGGGGGTGTGTCCTAGAGAGGxxxxxxxxxxxxxxxxxxxxxxxxxxxxxxxxxxxxxxxxxxxxxxxx97xxxxxxxxxxxxxxxxxxxxxxxxxxxxxxxxxxxxxxxxxxxxxxxCGCTGAGCACGGTGCGCGGCTGCTACGAGGCGGTGTGCTGCCTGTCGGAACGTAGCCTGGCCATTGCGCGAGGCCGCGGTAAGAGCCCGTCGACCGAGGAGCCTCTTAGCCTCTTGGACGACATGAACCACTGCTACTxxx7xxxG**CGG**GAACTGGAGCCGTGAGTCCCGCGAGGCACTCAGCTTAGCCAGGT

wtCCTCGGCGTCAGACCAGCCTAAGGAAGCCTGTTAGCAATTTAAACCCACTGTGAACGCCCAGGACCGGGGAGGCGGTGCCCAGGGCGGGCGGGGGTGGACCCTTGGCGGTCTGTTTTGAATAAGGGGGTGTGTCCTAGAGAGGACTCTATAAGAGTCGGCCGCTGCAGGCGTGCGCGCACTGTTTGCTGCTTTAGGTGTCTCTTTTCCTCCCTCTCTATCTCTACTCTCCAAC**ATG**A**AGG**CGCTGAGCCCGGTGCGCGGCTGCTACGAGGCGGTGTGCTGCCTGTCGGAACGTAGCCTGGCCATTGCGCGAGGCCGCGGTAAGAGCCCGTCGACCGAGGAGCCTCTTAGCCTCTTGGACGACATGAACCACTGCTACTCGCGCCTG**CGG**GAACTGGTGCCGGGAGTCCCGCGAGGCACTCAGCTTAGCCAGGT

**#17 ID4 [+1fs; 0] [+1fs; 0] [-188*] [-9inf; 0]**

wtCGCTCTACCGCTTGTCGCGGTCCTCTCGCGCAGGAAGCGCGCG**ATG**AAGG**CGG**TGAGCCCGGTGCGCCCCTCGGGCCGCAAGGCGCCGTCGGGCTGCGGCGGCGGGGAGCTGGCGCTACGCTGCCTGGCGGAGCACGGCCACAGCCTGGGTGGCTCGGCAGCCGCCGCCGCCGCTGCGGCGGCCGCGCGCTGCAAGGCGGCCGAGGCGGCGGCCGATGAGCCGGCGCTGTG**CCT**GCAGTGCGATATGAACGACTGCTACAGTCGCCTGCGGAGGCTCGTGCCTACCAT

**↓A**

**A1**CGCTCTACCGCTTGTCGCGGTCCTCTCGCGCAGGAAGCGCGCG**ATG**AAGG**CGG**TGAGCCCGGTGCGCCCCTCGGGCCGCAAGGCGCCGTCGGGCTGCGGCGGCGGGGAGCTGGCGCTACGCTGCCTGGCGGAGCACGGCCACAGCCTGGGTGGCTCGGCAGCCGCCGCCGCCGCTGCGGCGGCCGCGCGCTGCAAGGCGGCCGAGGCGGCGGCCGATGAGCCGGCGCTGTG**CCT**GCAGTGCGATATGAACGACTGCTACAGTCGCCTGCGGAGGCTCGTGCCTACCAT

**↓G**

**A2**CGCTCTACCGCTTGTCGCGGTCCTCTCGCGCAGGAAGCGCGCG**ATG**AAGG**CGG**TGAGCCCGGTGCGCCCCTCGGGCCGCAAGGCGCCGTCGGGCTGCGGCGGCGGGGAGCTGGCGCTACGCTGCCTGGCGGAGCACGGCCACAGCCTGGGTGGCTCGGCAGCCGCCGCCGCCGCTGCGGCGGCCGCGCGCTGCAAGGCGGCCGAGGCGGCGGCCGATGAGCCGGCGCTGTG**CCT**GCAGTGCGATATGAACGACTGCTACAGTCGCCTGCGGAGGCTCGTGCCTACCAT

**A3**CGCTCTACCGCTTGTCGCGGTCCTCTCGCGCAGGAAGCGCGCG**ATG**AAGGxxxxxxxxxxxxxxxxx188xxxxxxxxxxxxxxxxxxxxxxxxxxxxxxxxxxxxxxxxxxxxxxxxxxxxxxxxxxxxxxxxxxxxxxxxxxxxxxxxxxxxxxxxxxxxxxxxxxxxxxxxxxxxxxxxxxxxxxxxxxxxxxxxxxxxxxxxxxxxxxxxxxxxxxxxxxxxxxxxxxxxxxxxTGCGATATGAACGACTGCTACAGTCGCCTGCGGAGGCTCGTGCCTACCAT

**A4**CGCTCTACCGCTTGTCGCGGTCCTCTCGCGCAGGAAGCGCGCG**ATG**Axxxx9xxxxGCCCGGTGCGCCCCTCGGGCCGCAAGGCGCCGTCGGGCTGCGGCGGCGGGGAGCTGGCGCCACGCTGCCTGGCGGAGCACGGCCATAGCCTGGGTGGCTCGGCAGCCGCCGCCGCCGCTGCGGCGGCCGCGCGCTGCAAGGCGGCCGAGGCGGCGGCCGATGAGCCGTCGCTGTG**CCT**GCAGTGCGATATGAACGACTGCTACAGTCGCCTGCGGAGGCTCGTGCCTACCAT

**-------------------------------------------------------------------------------------------------------------------------------------------------**

**#19 ID1 [+11-2*; -22] [-25*; -139] [-25*; -9]**

wtTGTTCTCAGCCTCCTCCGCTCCCCTCCGCCTGTTCTCAGGATC**ATG**A**AGG**TCGCCAGTGGCAGTGCCGCAGCCGCTGCAGGCCCTAGCTGTTCGCTGAAGGCGGGCAGGACAGCGGGCGAGGTGGTACTTGGTCTGTCGGAGCAAAGCGTGGCCATCTCGCGCTGCGCTGGGACGCGCCTGCCCGCCTTGCTGGACGAGCAGCAGGTGAACGTCCTGCTCTACGACATGAA**CGG**CTGCTACTCACGCCTCAAGGAGCTGGTGCCCACCCTGCCCCAGAACCGCAAAGT

**↓TCAGGGCCTGA**

**A1**TGTTCTCAGCCTCCTCCGCTCCCCTCCGCCTGTTCTCAGGATCxx**G**A**AGG**TCGCCAGTGGCAGTGCCGCAGCCGCTGCAGGCCCTAGCTGTTCGCTGAAGGCGGGCAGGACAGCGGGCGAGGTGGTACTTGGTCTGTCGGAGCAAAGCGTGGCCATCTCGCGCTGCGCTGGGACGCGCCTGCCCGCCTTGCTGGACGAGCAGCAGGTGAACGTCCTGCTCTACGxxxxxxxxxx22xxxxxxxxxxCCTCAAGGAGCTGGTGCCCACCCTGCCACAGAACCGCAAAGT

**A2**TGTTCTCAGCCTCCTCCGCTCCCCTCCGCCTGTxxxxxxxxxx25xxxxxxxxxxxxxGGCAGTGCCGCAGCCGCTGCAGGCCCTAGCTGxxxxxxx139xxxxxxxxxxxxxxxxxxxxxxxxxxxxxxxxxxxxxxxxxxxxxxxxxxxxxxxxxxxxxxxxxxxxxxxxxxxxxxxxxxxxxxxxxxxxxxxxxxxxxxxxxxxxxxxxxxxxxxxxxxxxxxxxxAA**CGG**CTGCTACTCACGCCTCAAGGAGCTGGTGCCCACCCTGCCACAGAACCGCAAAGT

**A3**TGTTCTCAGCCTCCTCCGCTCCCCTCCGCCTGTxxxxxxxxxx25xxxxxxxxxxxxxGGCAGTGCCGCAGCCGCTGCAGGCCCTAGCTGTTCGCTGAAGGCGGGCAGGACAGCGGGCGAGGTGGTACTTGGTCTGTCGGAGCAAAGCGTGGCCATCTCGCGCTGCGCTGGGACGCGCCTGCCCGCCTTGCTGGACGAGCAGCAGGTGAACGTCCTGCTCTAxxxx9xxxx**CGG**CTGCTACTCACGCCTCTGGGAGCTGGTGCCCACCCTGCCACAGAACCGCAAAGT

**#19 ID2 [0; -25fs] [+9-121fs] [0; +1fs]**

wtCTCAGCCCCCTGTGGCGGCTCCCTCCCGGTCTTCCTCCTACGAGCAGC**ATG**AAAGCCTTCAGTCCGGTG**AGG**TCCGTTAGGAAAAACAGCCTGTCGGACCACAGCTTGGGCATCTCCCGGAGCAAAACCCCGGTGGACGACCCGATGAGTCTGCTCTACAACATGAACGACTGCTACTCCAAGCTCAAGGAAC**TGG**TGCCCAGCATCCCCCAGAACAAGAAGGTGACCAAGATGGAAATCCTGCAGCACGTCATCGATTACATCTTGGACCTGCAGATCGCCCTGGAC

**A1**CTCAGCCCCCTGTGGCGGCTCCCTCCCGGTCTTCCTCCTACGAGCAGC**ATG**AAAGCCTTCAGTCCGGTG**AGG**TCCGTTAGGAAAAACAGCCTGTCGGACCACAGCTTGGGCATCTCCCGGAGCAAAACCCCGGTGGACGACCCGATGAGTCTGCTCTACAACATxxxxxxxxxxx25xxxxxxxxxxxxGAAC**TGG**TGCCCAGCATCCCCCAGAACAAGAAGGTGACCAAGATGGAAATCCTGCAGCACGTCATCGATTACATCTTGGACCTGCAGATCGCCCTGGAC

**↓GAACTTCTG**

**A2**CTCAGCCCCCTGTGGCGGCTCCCTCCCGGTCTTCCTCCTACGAGCAGC**ATG**AAAGCCTTCAGTCCGGxxxxxxxx9x121xxxxxxxxxxxxxxxxxxxxxxxxxxxxxxxxxxxxxxxxxxxxxxxxxxxxxxxxxxxxxxxxxxxxxxxxxxxxxxxxxxxxxxxxxxxxxxxxxxxxxxxxxxxxGGAAC**TGG**TGCCCAGCATCCCCCAGAACAAGAAGGTGACCAAGATGGAAATCCTGCAGCACGTCATCGATTACATCTTGGACCTGCAGATCGCCCTGGAC

**A3**CTCAGCCCCCTGTGGCGGCTCCCTCCCGGTCTTCCTCCTACGAGCAGC**ATG**AAAGCCTTCAGTCCGGTG**AGG**TCCGTTAGGAAAAACAGCCTGTCGGACCACAGCTTGGGCATCTCCCGGAGCAAAACCCCGGTGGACGACCCGATGAGTCTGCTCTACAACATGAACGACTGCTACTCCAAGCTCAAGGAAC**TGG**TGCCCAGCATCCCCCAGAACAAGAAGGTGACCAAGATGGAAATCCTGCAGCACGTCATCGATTACATCTTGGACCTGCAGATCGCCCTGGA

**T↑**

**#19 ID3 [-34*; -6] [-34*; 0] [-16*; 0] [-16*; -6] [-2*; 0] [-2*; -6]**

wtTCTATAAGAGTCGGCCGCTGCAGGCGTGCGCGCACTGTTTGCTGCTTTAGGTGTCTCTTTTCCTCCCTCTCTATCTCTACTCTCCAAC**ATG**A**AGG**CGCTGAGCCCGGTGCGCGGCTGCTACGAGGCGGTGTGCTGCCTGTCGGAACGTAGCCTGGCCATTGCGCGAGGCCGCGGTAAGAGCCCGTCGACCGAGGAGCCTCTTAGCCTCTTGGACGACATGAACCACTGCTACTCGCGCCTG**CGG**GAACTGGTGCCGGGAGTCCCGCGAGGCACTCAGCTTAGCCAGGT

**A1**TCTATAAGAGTCGGCCGCTGCAGGCGTGCGCGCACTGTTTGCTGCTTTAGGTGTCTCTTTTCCTCCCTCTCTATCxxxxxxxxxxxxxxxxxxxx34xxxxxxxxxxxGxGCGGCTGCTACGAGGCGGTGTGCTGCCTGTCGGAACGTAGCCTGGCCATTGCGCGAGGCCGCGGTAAGAGCCCGTCGACCGAGGAGCCTCTTAGCCTCTTGGACGACATGAACCACTGCTACTCGxx6xxx**CGG**GAACTGGTGCCGGGAGTCCCGCGAGGCACTCAGCTTAGCCAGGT

**A2**TCTATAAGAGTCGGCCGCTGCAGGCGTGCGCGCACTGTTTGCTGCTTTAGGTGTCTCTTTTCCTCCCTCTCTATCxxxxxxxxxxxxxxxxxxxx34xxxxxxxxxxxGxGCGGCTGCTACGAGGCGGTGTGCTGCCTGTCGGAACGTAGCCTGGCCATTGCGCGAGGCCGCGGTAAGAGCCCGTCGACCGAGGAGCCTCTTAGCCTCTTGGACGACATGAACCACTGCTACTCGCGCCTG**CGG**GAACTGGTGCCGGGAGTCCCGCGAGGCACTCAGCTTAGCCAGGT

**A3**TCTATAAGAGTCGGCCGCTGCAGGCGTGCGCGCACTGTTTGCTGCTTTAGGTGTCTCTTTTCCTCCCTCTCTATCTCTACTCTxxxxxxx16xxxxxxxGAGCCCGGTGCGCGGCTGCTACGAGGCGGTGTGCTGCCTGTCGGAACGTAGCCTGGCCATTGCGCGAGGCCGCGGTAAGAGCCCGTCGACCGAGGAGCCTCTTAGCCTCTTGGACGACATGAACCACTGCTACTCGCGCCTG**CGG**GAACTGGTGCCGGGAGTCCCGCGAGGCACTCAGCTTAGCCAGGT

**A4**TCTATAAGAGTCGGCCGCTGCAGGCGTGCGCGCACTGTTTGCTGCTTTAGGTGTCTCTTTTCCTCCCTCTCTATCTCTACTCTxxxxxxx16xxxxxxxGAGCCCGGTGCGCGGCTGCTACGAGGCGGTGTGCTGCCTGTCGGAACGTAGCCTGGCCATTGCGCGAGGCCGCGGTAAGAGCCCGTCGACCGAGGAGCCTCTTAGCCTCTTGGACGACATGAACCACTGCTACTCGxx6xxx**CGG**GAACTGGTGCCGGGGGTCCCGCGAGGCACTCAGCTTAGCCAGGT

**A5**TCTATAAGAGTCGGCCGCTGCAGGCGTGCGCGCACTGTTTGCTGCTTTAGGTGTCTCTTTTCCTCCCTCTCTATCTCTACTCTCCAAC**A**xx**AAG**GCGCTGAGCCCGGTGCGCGGCTGCTACGAGGCGGTGTGCTGCCTGTCGGAACGTAGCCTGGCCATTGCGCGAGGCCGCGGTAAGAGCCCGTCGACCGAGGAGCCTCTTAGCCTCTTGGACGACATGAACCACTGCTACTCGCGCCTG**CGG**GAACTGGTGCCGGGGGTCCCGCGAGGCACTCAGCTTAGCCAGGT

**A6**TCTATAAGAGTCGGCCGCTGCAGGCGTGCGCGCACTGTTTGCTGCTTTAGGTGTCTCTTTTCCTCCCTCTCTATCTCTACTCTCCAAC**A**xx**AAG**GCGCTGAGCCCGGTGCGCGGCTGCTACGAGGCGGTGTGCTGCCTGTCGGAACGTAGCCTGGCCATTGCGCGAGGCCGCGGTAAGAGCCCGTCGACCGAGTAGCCTCTTAGCCTCTTGGACGACATGAACCACTGCTACTCGxx6xxx**CGG**GAACTGGTGCCGGGAGTCCCGCGAGGCACTCAGCTTAGCCAGGT

**#19 ID4 [WT] [-53*; 0] [-190fs] [-9inf; 0]**

wtCGCTCTACCGCTTGTCGCGGTCCTCTCGCGCAGGAAGCGCGCG**ATG**AAGG**CGG**TGAGCCCGGTGCGCCCCTCGGGCCGCAAGGCGCCGTCGGGCTGCGGCGGCGGGGAGCTGGCGCTACGCTGCCTGGCGGAGCACGGCCACAGCCTGGGTGGCTCGGCAGCCGCCGCCGCCGCTGCGGCGGCCGCGCGCTGCAAGGCGGCCGAGGCGGCGGCCGATGAGCCGGCGCTGTG**CCT**GCAGTGCGATATGAACGACTGCTACAGTCGCCTGCGGAGGCTCGTGCCTACCAT

**A1**CGCTCTACCGCTTGTCGCGGTCCTCTCGCGCAGGAAGCGCGCG**ATG**AAGG**CGG**TGAGCCCGGTGCGCCCCTCGGGCCGCAAGGCGCCGTCGGGCAGCGGCGGCGGGGAGCTGGCGCTACGCTGCCTGGCGGAGCACGGCCACAGCCTGGGTGGCTCGGCAGCCGCCGCCGCCGCTGCGGCGGCCGCGCGCTGCAAGGCGGCCGAGGCGGCGGCCGATGAGCCGGCGCTGTG**CCT**GCAGTGCGATATGAACGACTGCTACAGTCGCCTGCGGAGGCTCGTGCCTACCAT

**A2**CGCTCTACCGCTTGTCGCGGTCCTCTCGCGCAGCGTCGGxxxxxxxxxxxxxxxxxxxxx53xxxxxxxxxxxxxxxxxxxxxxxxxxxxxxGCTGCGGCGGCGGGGAGCTGGCGCTACGCTGCCTGGCGGAGCACGGCCACAGCCTGGGTGGCTCGGCAGCCGCCGCCGCCGCTGCGGCGGGCGCGCGCTGCAAGGCGGCCGAGGCGGCGGCCGATGAGCAGGCGCTGTG**CCT**GCAGTGCGATATGAACGACTGCTACAGTCGCCTGCGGAGGCTCGTGCCTACCAT

**A3**CGCTCTACCGCTTGTCGCGGTCCTCTCGCGCAGGAAGCGCGCG**ATG**xxxxxxxxxxxxxxxxxxxxxxxx190xxxxxxxxxxxxxxxxxxxxxxxxxxxxxxxxxxxxxxxxxxxxxxxxxxxxxxxxxxxxxxxxxxxxxxxxxxxxxxxxxxxxxxxxxxxxxxxxxxxxxxxxxxxxxxxxxxxxxxxxxxxxxxxxxxxxxxxxxxxxxxxxxxxxxxxxxxxxxxxxxxxAGTGCGATATGAACGACTGCTACAGTCGCCTGCGGAGGCTCGTGCCTACCAT

**A4**CGCTCTACCGCTTGTCGCGGTCCTCTCGCGCAGGAAGCGCGCG**ATG**Axxxx9xxxxGCCCGGTGCGCCCCTCGGGCCGCAAGGCGCCGTCGGGCTGCGGCGGCGGGGAGCTGGCGCTACGCTGCCTGGCGGAGCACGGCCACAGCCTGGGTGGCTCGGCAGCCGCCGCCGCCGCTGCGGCGGCCGCGCGCTGCAAGGCGGCCGAGGCGGCGGCCGATGAGCCGGCGCTGTG**CCT**GCAGTGCGATATGAACGACTGCTACAGTCGCCTGCGGAGGCTCGTGCCTACCAT

**-------------------------------------------------------------------------------------------------------------------------------------------------**

**#22 ID1 [+1*; -20] [-192*]**

wtTGTTCTCAGCCTCCTCCGCTCCCCTCCGCCTGTTCTCAGGATC**ATG**A**AGG**TCGCCAGTGGCAGTGCCGCAGCCGCTGCAGGCCCTAGCTGTTCGCTGAAGGCGGGCAGGACAGCGGGCGAGGTGGTACTTGGTCTGTCGGAGCAAAGCGTGGCCATCTCGCGCTGCGCTGGGACGCGCCTGCCCGCCTTGCTGGACGAGCAGCAGGTGAACGTCCTGCTCTACGACATGAA**CGG**CTGCTACTCACGCCTCAAGGAGCTGGTGCCCACCCTGCCCCAGAACCGCAAAGT

**↓T**

**A1**TGTTCTCAGCCTCCTCCGCTCCCCTCCGCCTGTTCTCAGGATC**ATG**A**AGG**TCGCCAGTGGCAGTGCCGCAGCCGCTGCAGGCCCTAGCTGTTCGCTGAAGGCGGGCAGGACAGCGGGCGAGGTGGTACTTGGTCTGTCGGAGCAAAGCGTGGCCATCTCGCGCTGCGCTGGGACGCGCCTGCCCGCCTTGCTGGACGAGCAGCAGGTGAACGxxAxxxxxxxxx20xxxxxxx**G**CTGCTACTCACGCCTCAAGGAGCTGGTGCCCACCCTGCCCCAGAACCGCAAAGT

**A2**TGTTCTCAGCCTCCTCCGCTCCCCTCCGCCTGTTCxxxxxxxxxxxxxxxxxxxxxxxxxxxxxxxxxxx192xxxxxxxxxxxxxxxxxxxxxxxxxxxxxxxxxxxxxxxxxxxxxxxxxxxxxxxxxxxxxxxxxxxxxxxxxxxxxxxxxxxxxxxxxxxxxxxxxxxxxxxxxxxxxxxxxxxxxxxxxxxxxxxxxxxxxxxxxxxxxxxxxxxxxxxxxxTGAA**CGG**CTGCTACTCACGCCTCAAGGAGCTGGTGCCCACCCTGCCCCAGAACCGCAAAGT

**#22 ID2 [+1fs STOP; -16bif] [+1fs STOP; -16bif] [+112 STOP; -16bif] [-10fs STOP; -407sd] [+1fs STOP; -407sd]**

wtCTCAGCCCCCTGTGGCGGCTCCCTCCCGGTCTTCCTCCTACGAGCAGC**ATG**AAAGCCTTCAGTCCGGTG**AGG**TCCGTTAGGAAAAACAGCCTGTCGGACCACAGCTTGGGCATCTCCCGGAGCAAAACCCCGGTGGACGACCCGATGAGTCTGCTCTACAACATGAACGACTGCTACTCCAAGCTCAAGGAAC**TGG**TGCCCAGCATCCCCCAGAACAAGAAGGTGACCAAGATGGAAATCCTGCAGCACGTCATCGATTACATCTTGGACCTGCAGATCGCCCTGGAC

**↓G**

**A1**CTCAGCCCCCTGTGGCGGCTCCCTCCCGGTCTTCCTCCTACGAGCAGC**ATG**AAAGCCTTCAGTCCGGTG**AGG**TCCGTTAGGAAAAACAGCCTGTCGGACCACAGCTTGGGCATCTCCCGGAGCAAAACCCCGGTGGACGACCCGATGAGTCTGCTCTACAACATGAACGACTGCTxxxxxxx16xxxxxxxAC**TGG**TGCCCAGCATCCCCCAGAACAAGAAGGTGACCAAGATGGAAATCCTGCAGCACGTCATCGATTACATCTTGGACCTGCAGATCGCCCTGGAC

**↓A**

**A2**CTCAGCCCCCTGTGGCGGCTCCCTCCCGGTCTTCCTCCTACGAGCAGC**ATG**AAAGCCTTCAGTCCGGTG**AGG**TCCGTTAGGAAAAACAGCCTGTCGGACCACAGCTTGGGCATCTCCCGGAGCAAAACCCCGGTGGACGACCCGATGAGTCTGCTCTACAACATGAACGACTGCTxxxxxxx16xxxxxxxAC**TGG**TGCCCAGCATCCCCCAGAACAAGAAGGTGACCAAGATGGAAATCCTGCAGCACGTCATCGATTACATCTTGGACCTGCAGATCGCCCTGGAC

**↓G**

**A3**CTCAGCCCCCTGTGGCGGCTCCCTCCCGGTCTTCCTCCTACGAGCAGC**ATG**AAAGCCTTCAGTCCGGTG**AGG**TCCGTTAGGAAAAACAGCCTGTCGGACCACAGCTTGGGCATCTCCCGGAGCAAAACCCCGGTGGACGACCCGATGAGTCTGCTCTACAACATGAACGACTGCTxxxxxxx16xxxxxxxAC**TGG**TGCCCAGCATCCCCCAGAACAAGAAGGTGACCAAGATGGAAATCCTGCAGCACGTCATCGATTACATCTTGGACCTGCAGATCGCCCTGGAC

**↑**

**ACTCGGTGCCACTTTTTCAAGTTGATAACGGACTAGCCTTATTTTAACTTGCTATTTCTAGCTCTAAAACTCATGATCCTGAGAACAGGCCTATAGTGAGTCGTATTACGCG**

**A4**CTCAGCCCCCTGTGGCGGCTCCCTCCCGGTCTTCCTCCTACGAGCAGC**ATG**AAAGCCTTCAGTCCGxxxx10xxxxTTAGGAAAAACAGCCTGTCGGACCACAGCTTGGGCATCTCCCGGAGCAAAACCCCGGTGGACGACCCGATGAGTCTGCTCTACAACATGAACxxxxxxxxxxxxxxxxxxxxxxxxxxxxxxxxxxxxxxxxxxxxxxxxxxxxxxxxxxxxxxxxxxxxxxxxxxxxxxxxxxxxxxxxxxxxxxxxxxxxxxxxxxxxxxxxxxxxxxxxxxxxxxxxxxxxxxxxxxxxxxxxxxxxxxxxxxxxxxxxxxxxxxxxxxxxxxxxxxxxxxxxxxxxxxxxxxxxxxxxx407xxxxxxxxxxxxxxxxxxxxxxxxxxxxxxxxxxxxxxxxxxxxxxxxxxxxxxxxxxxxxxxxxxxxxxxxxxxxxxxxxxxxxxxxxxxxxxxxxxxxxxxxxxxxxxxxxxxxxxxxxxxxxxxxxxxxxxxxxxxxxxxxxxxxxxxxxxxxxxxxxxxxxxxxxxxxxxxxxxxxxxxxxxxxxxxxxxxxxxxxxxxagc

**↓G**

**A5**CTCAGCCCCCTGTGGCGGCTCCCTCCCGGTCTTCCTCCTACGAGCAGC**ATG**AAAGCCTTCAGTCCGGTG**AGG**TCCGTTAGGAAAAACAGCCTGTCGGACCACAGCTTGGGCATCTCCCGGAGCAAAACCCCGGTGGACGACCCGATGAGTCTTCTCTACAACATGAACxxxxxxxxxxxxxxxxxxxxxxxxxxxxxxxxxxxxxxxxxxxxxxxxxxxxxxxxxxxxxxxxxxxxxxxxxxxxxxxxxxxxxxxxxxxxxxxxxxxxxxxxxxxxxxxxxxxxxxxxxxxxxxxxxxxxxxxxxxxxxxxxxxxxxxxxxxxxxxxxxxxxxxxxxxxxxxxxxxxxxxxxxxxxxxxxxxxxxxxxx407xxxxxxxxxxxxxxxxxxxxxxxxxxxxxxxxxxxxxxxxxxxxxxxxxxxxxxxxxxxxxxxxxxxxxxxxxxxxxxxxxxxxxxxxxxxxxxxxxxxxxxxxxxxxxxxxxxxxxxxxxxxxxxxxxxxxxxxxxxxxxxxxxxxxxxxxxxxxxxxxxxxxxxxxxxxxxxxxxxxxxxxxxxxxxxxxxxxxxxxxxxxagc

**#22 ID3 [+9-16*; -6] [-160*]**

wtTCTATAAGAGTCGGCCGCTGCAGGCGTGCGCGCACTGTTTGCTGCTTTAGGTGTCTCTTTTCCTCCCTCTCTATCTCTACTCTCCAAC**ATG**A**AGG**CGCTGAGCCCGGTGCGCGGCTGCTACGAGGCGGTGTGCTGCCTGTCGGAACGTAGCCTGGCCATTGCGCGAGGCCGCGGTAAGAGCCCGTCGACCGAGGAGCCTCTTAGCCTCTTGGACGACATGAACCACTGCTACTCGCGCCTG**CGG**GAACTGGTGCCGGGAGTCCCGCGAGGCACTCAGCTTAGCCAGGT

**↓TCTCTCTCT**

**A1**TCTATAAGAGTCGGCCGCTGCAGGCGTGCGCGCACTGTTTGCTGCTTTAGGTGTCTCTTTTCCTCCCTCTCTATCTCTACTCTCCAACxxxxxxxx9x16xxxxCGGTGCGCGGCTGCTACGAGGCGGTGTGCTGCCTGTCGGAACGTAGCCTGGCCATTGCGCGAGGCCGCGGTAAGAGCCCGTCGACCGAGGAGCCTCTTAGCCTCTTGGACGACATGAACCACTGCTACTCGxx6xxx**CGG**GAACTGGTGCCGGGAGTCCCGCGAGGCACTCAGCTTAGCCAGGT

**A2**TCTATAAGAGTCGGCCGCTGCAGGCGTGCGCGCACTGTTTGCTGCTTTAGGTGTCTCTTTTCCTCCCTCTCTATCTCTACTCTCCAACxxxxxxxxxxxx160xxxxxxxxxxxxxxxxxxxxxxxxxxxxxxxxxxxxxxxxxxxxxxxxxxxxxxxxxxxxxxxxxxxxxxxxxxxxxxxxxxxxxxxxxxxxxxxxxxxxxxxxxxxxxxxxxxxxxxxxxxxxxxxxxxxxxxxxxxxxxxxxxTGGTGCCGGGAGTCCCGCGAGGCACTCAGCTTAGCCAGGT

**#22 ID4 [+190inv-190 STOP] [-172fs; 0] [-189inf]**

wtCGCTCTACCGCTTGTCGCGGTCCTCTCGCGCAGGAAGCGCGCG**ATG**AAGG**CGG**TGAGCCCGGTGCGCCCCTCGGGCCGCAAGGCGCCGTCGGGCTGCGGCGGCGGGGAGCTGGCGCTACGCTGCCTGGCGGAGCACGGCCACAGCCTGGGTGGCTCGGCAGCCGCCGCCGCCGCTGCGGCGGCCGCGCGCTGCAAGGCGGCCGAGGCGGCGGCCGATGAGCCGGCGCTGTG**CCT**GCAGTGCGATATGAACGACTGCTACAGTCGCCTGCGGAGGCTCGTGCCTACCAT

**A1**CGCTCTACCGCTTGTCGCGGTCCTCTCGCGCAGGAAGCGCGCG**ATG**Axxxxxxxxxxxxxxxxxxxxxxxxxxxxxxxxx190xxxxxxxxxxxxxxxxxxxxxxxxxxxxxxxxxxxxxxxxxxxxxxxxxxxxxxxxxxxxxxxxxxxxxxxxxxxxxxxxxxxxxxxxxxxxxxxxxxxxxxxxxxxxxxxxxxxxxxxxxxxxxxxxxxxxxxxxxxxxxxxxxxxxxxxxxxGTGCGATATGAACGACTGCTACAGTCGCCTGCGGAGGCTCGTGCCTACCAT

**↑**

**TGCAGGCACAGCGCCGGCTCATCGGCCGCCGCCTCGTCCGCCTTGCAGCGCGCGGCCGCCGCAGCGGCGGCGGCGGCTGCCGAGCCACCCAGGCTGTGGCCGTGCTCCGCCAGGCAGCGTAGCGCCAGCTCCCCGCCGCCGCAGCCCGACGGCGCCTTGCGGCCCGAGGGGCGCACCGGGCTCACCGCCT**

**A2**CGCTCTACCGCTTGTCGCGGTCCTCTCGCGCAGAAAGCGCGCG**ATG**Axxxxxxxxxxxxxxxxxxx172xxxxxxxxxxxxxxxxxxxxxxxxxxxxxxxxxxxxxxxxxxxxxxxxxxxxxxxxxxxxxxxxxxxxxxxxxxxxxxxxxxxxxxxxxxxxxxxxxxxxxxxxxxxxxxxxxxxxxxxxxxxxxxxxxxxxxxxxxxxxxxxxxxxxxxGCCGGCGCTGTG**CCT**GCA.TCATCGCGCGCTTCATGCGCGAGAGGACCGCGA

**A3**CGCTCTACCGCTTGTCGCGGTCCTCTCGCGCAGGAAGCGCGCG**ATG**Axxxxxxxxxxxxxxxxxxxxxx189xxxxxxxxxxxxxxxxxxxxxxxxxxxxxxxxxxxxxxxxxxxxxxxxxxxxxxxxxxxxxxxxxxxxxxxxxxxxxxxxxxxxxxxxxxxxxxxxxxxxxxxxxxxxxxxxxxxxxxxxxxxxxxxxxxxxxxxxxxxxxxxxxxxxxxxxxxxxxxxxxxxxTGTGCGATATGAACGACTGCTACAGTCGCCTGCGGAGGCTCGTGCCTACCAT

**-------------------------------------------------------------------------------------------------------------------------------------------------**

**#23 ID1 (-194*] [-6*; +1]**

wtTGTTCTCAGCCTCCTCCGCTCCCCTCCGCCTGTTCTCAGGATC**ATG**A**AGG**TCGCCAGTGGCAGTGCCGCAGCCGCTGCAGGCCCTAGCTGTTCGCTGAAGGCGGGCAGGACAGCGGGCGAGGTGGTACTTGGTCTGTCGGAGCAAAGCGTGGCCATCTCGCGCTGCGCTGGGACGCGCCTGCCCGCCTTGCTGGACGAGCAGCAGGTGAACGTCCTGCTCTACGACATGAA**CGG**CTGCTACTCACGCCTCAAGGAGCTGGTGCCCACCCTGCCCCAGAACCGCAAAGT

**A1**TGTTCTCAGCCTCCTCCGCTCCCCTCCGCCTGTTCTCAGGATCxxxxxxxxxxxxxxxxxxxxxxxxxxxxxxxxxxxxxx194xxxxxxxxxxxxxxxxxxxxxxxxxxxxxxxxxxxxxxxxxxxxxxxxxxxxxxxxxxxxxxxxxxxxxxxxxxxxxxxxxxxxxxxxxxxxxxxxxxxxxxxxxxxxxxxxxxxxxxxxxxxxxxxxxxxxxxxxxxxxxxxxxxxxxxxxGxTACTCACGCCTCAAGGAGCTGGTGCCCACCCTGCCCCAGAACCGCAAAGT

**A2**TGTTCTCAGCCTCCTCCGCTCCCCTCCGCCTGTTCTCAGGAxxx6xx**AGG**TCGCCAGTGGCAGTGCCGCAGCCGCTGCAGGCCCTAGCTGTTCGCTGAAGGCGGGCAGGACAGCGGGCGAGGTGGTACTTGGTCTGTCGGAGCAAAGCGTGGCCATCTCGCGCTGCGCTGGGACGCGCCTGCCCGCCTTGCTGGACGAGCAGCAGGTGAACGTCCTGCTCTACGACATGAA**CGG**CTGCTACTCACGCCTCAAGGAGCTGGTGCCCACCCTGCCCCAGAACCGCAAAGT

**T↑**

**#23 ID2 [+4fs; -53] [-10fs; -271] [+3-1fs STOP; -20bif] [-144inf]**

wtCTCAGCCCCCTGTGGCGGCTCCCTCCCGGTCTTCCTCCTACGAGCAGC**ATG**AAAGCCTTCAGTCCGGTG**AGG**TCCGTTAGGAAAAACAGCCTGTCGGACCACAGCTTGGGCATCTCCCGGAGCAAAACCCCGGTGGACGACCCGATGAGTCTGCTCTACAACATGAACGACTGCTACTCCAAGCTCAAGGAAC**TGG**TGCCCAGCATCCCCCAGAACAAGAAGGTGACCAAGATGGAAATCCTGCAGCACGTCATCGATTACATCTTGGACCTGCAGATCGCCCTGGAC

**↓TTAG**

**A1**CTCAGCCCCCTGTGGCGGCTCCCTCCCGGTCTTCCTCCTACGAGCAGC**ATG**AAAGCCTTCAGTCCGGTG**AGG**TCCGTTAGGAAAAACAGCCTGTCGGACCACAGCTTGGGCATCTCCCGGAGCAAAACCCCGGTxxxxxxxxxxxxxxxxxxxxxxxxx53xxxxxxxxxxxxxxxxxxxxxxxxxxAGGAAC**TGG**TGCCCAGCATCCCCCAGAACAAGAAGGTGACCAAGATGGAAATCCTGCAGCACGTCATCGATTACATCTTGGACCTGCAGATCGCCCTGTAG

**A2**CTCAGCCCCCTGTGGCGGCTCCCTCCCGGTCTTCCTCCTACGAGCAGC**ATG**AAAGCCTTCAxxxx10xxxx**G**TCCGTTAGGAAAAACAGCCTGTCGGACCACAGCTTGGGCATCTCCCGGAGCAAAACCCCGGTGGACGACCCGATGAGTCTGCTCTACAACATGAACGACTGCTACTCCAAGCTCxxxxxxxxxxxxxxxxxxxxxxxxxxxxxxxxxxxxxxxxxxxxxxxxxxxxxxxxxxxxxxxxxxxxxxxxxxxxxxxxxxxxxxxxxxxxxxxxxxxxxxxxxxxxxxxxxxxxxxxxxxxxxxxxxxxxxxxxxxxxxxxxxxxxxxxxxxxxxxxxxxxxxxxxx271xxxxxxxxxxxxxxxxxxxxxxxxxxxxxxxxxxxxxxxxxxxxxxxxxxxxxxxxxxxxxxxxxxxxxxxxxxxxxxxxxxxxxxxxxxxxxxxxxxxTTAAAGCCCATCGTAGAGACAGGTTCATTAACTTTATTTTTGAGGAAACTGTATATTGAGCGTCATGTGAAATCGCTACTTATAAGTTCTGTGTGGGTTGCATCTGGATCTGCGCTGTAG

**↓AAA**

**A3**CTCAGCCCCCTGTGGCGGCTCCCTCCCGGTCTTCCTCCTACGAGCAGC**ATG**AAAGCCTTCAGTCxGGTG**AGG**TCCGTTAGGAAAAACAGCCTGTCGGACCACAGCTTGGGCATCTCCCGGAGCAAAACCCCGGTGGACGACCCGATGAGTCTGCTCTACAACATGAACGACTGCTACTCCAAGCxxxxxxxxxx20xxxxxxxxATCCCCCAGAACAAGAAGG

**A4**CTCAGCCCCCTGTGGCGGCTCCCTCCCGGTCTTCCACCTACGAGCAGC**ATG**AAAxxxxxxxxxxxxxxxxxxxxxxxxxxxxxxxx144xxxxxxxxxxxxxxxxxxxxxxxxxxxxxxxxxxxxxxxxxxxxxxxxxxxxxxxxxxxxxxxxxxxxxxxxxxxxxxxxxxxxxxxxxxxxxxxxxxxxxxxxxxxxxGCCCAGCATCCCCCAGAACAAGAAGGTGACCAAGATGGAAATCCTGCAGCACGTCATCGATACATCTTGGACCTGCAGATCGCCCTGGAC

**#23 ID3 [-6*; +1-6] [-148*] [-160*]**

wtTCTATAAGAGTCGGCCGCTGCAGGCGTGCGCGCACTGTTTGCTGCTTTAGGTGTCTCTTTTCCTCCCTCTCTATCTCTACTCTCCAAC**ATG**A**AGG**CGCTGAGCCCGGTGCGCGGCTGCTACGAGGCGGTGTGCTGCCTGTCGGAACGTAGCCTGGCCATTGCGCGAGGCCGCGGTAAGAGCCCGTCGACCGAGGAGCCTCTTAGCCTCTTGGACGACATGAACCACTGCTACTCGCGCCTG**CGG**GAACTGGTGCCGGGAGTCCCGCGAGGCACTCAGCTTAGCCAGGT

**A1**TCTATAAGAGTCGGCCGCTGCAGGCGTGCGCGCACTGTTTGCTGCTTTAGGTGTCTCTTTTCCTCCCTCTCTATCTCTACTCTCCAAxx6xxx**GG**CGCTGAGCCCGGTGCGCGGCTGCTACGAGGCGGTGTGCTGCCTGTCGGAACGTAGCCTGGCCATTGCGCGAGGCCGCGGTAAGAGCCCGTCGACCGAGGAGCCTCTTAGCCTCTTGGACGACATGAA**CCA**CTGCTACTCGCGxx1x6xGGAACTGGTGCCGGGAGTCCCGCGAGGCACTCAGCTTAGCCAGGT

**↑A**

**A2**TCTATAAGAGTCGGCCGCTGCAGGCGTGCGCGCACTGTTTGCTGCTTTAGGTGTCTCTTTTCCTCCCTCTCTATCTCTACTCTCCAAC**A**xxAxxxxxxxxxx148xxxxxxxxxxxxxxxxxxxxxxxxxxxxxxxxxxxxxxxxxxxxxxxxxxxxxxxxxxxxxxxxxxxxxxxxxxxxxxxxxxxxxxxxxxxxxxxxxxxxxxxxxxxxxxxxxxxxxxxxxxxxxxxxxxxxxCTGCGGGAACTGGTGCCGGGAGTCCCGCGAGGCACTCAGCTTAGCCAGGT

**A3**TCTATAAGAGTCGGCCGCTGCAGGCGTGCGCGCACTGTTTGCTGCTTTAGGTGTCTCTTTTCCTCCCTCTCTATCTCTACTCTCCAACxxxxxxxxxxxxxxxx160xxxxxxxxxxxxxxxxxxxxxxxxxxxxxxxxxxxxxxxxxxxxxxxxxxxxxxxxxxxxxxxxxxxxxxxxxxxxxxxxxxxxxxxxxxxxxxxxxxxxxxxxxxxxxxxxxxxxxxxxxxxxxxxxxxxxxxxxxxxxxTGGTGCCGGGAGTCCCGCGAGGCACTCAGCTTAGCCAGGT

**#23 ID4 [211*] [+100-13*; 0]**

wtCGCTCTACCGCTTGTCGCGGTCCTCTCGCGCAGGAAGCGCGCG**ATG**AAGG**CGG**TGAGCCCGGTGCGCCCCTCGGGCCGCAAGGCGCCGTCGGGCTGCGGCGGCGGGGAGCTGGCGCTACGCTGCCTGGCGGAGCACGGCCACAGCCTGGGTGGCTCGGCAGCCGCCGCCGCCGCTGCGGCGGCCGCGCGCTGCAAGGCGGCCGAGGCGGCGGCCGATGAGCCGGCGCTGTG**CCT**GCAGTGCGATATGAACGACTGCTACAGTCGCCTGCGGAGGCTCGTGCCTACCAT

**A1**CGCTCTACCGCTTGTCGCGGTCCTCTCxxxxxxxxxxxxxxxxxxxxxxxxxxxxxxxxxxxxxxxx211xxxxxxxxxxxxxxxxxxxxxxxxxxxxxxxxxxxxxxxxxxxxxxxxxxxxxxxxxxxxxxxxxxxxxxxxxxxxxxxxxxxxxxxxxxxxxxxxxxxxxxxxxxxxxxxxxxxxxxxxxxxxxxxxxxxxxxxxxxxxxxxxxxxxxxxxxxxxxxxxxxxxxxxxTGCGATATGAACGACTGCTACAGTCGCCTGCGGAGGCTCGTGCCTACCAT

**↓AGTCGTTCATATCGCACTGCGTTTTAGAGCTAGAAATAGCAAGTTAAAATAAGGCTAGTCCGTTATCAACTTGAAAAAGTTGCACCGAGTCGGTGCTTTT**

**A2**CGCTCTACCGCTTGTCGCGGTCCTCTCGCGCAGGxxxxx13xxxxxxAGG**CGG**TGAGCCCGGTGCGCCCCTCGGGCCGCAAGGCGCCGTCGGGCTGCGGCGGCGGGGAGCTGGCGCTACGCTGCCTGGCGGAGCACGGCCACAGCCTGGGTGGCTCGGCAGCCGCCGCCGCCGCTGCGGCGGCCGCGCGCTGCAAGGCGGCCGAGGCGGCGGCCGATGAGCCGGCGCTGTG**CCT**GCAGTGCGATATGAACGACTGCTACAGTCGCCTGCGGAGGCTCGTGCCTACCAT
